# Bioinspired Liquid‐Free Ion‐Conductive Elastomers with Ultrahigh Mechanical Strength and Excellent Ionic Conductivity for Multifunctional Flexible Sensing Applications

**DOI:** 10.1002/advs.202503510

**Published:** 2025-04-28

**Authors:** Zequan Li, Jingjing Tang, Xuwei Wang, Fuqi Wang, Fangyan Ou, Wenyu Pan, Changsheng Wang, Ting Xie, Chuang Ning, Xiwei Xu, Jiamin Liu, Qihua Liang, Wei Gao, Shuangliang Zhao

**Affiliations:** ^1^ School of Resources Environment and Materials Guangxi University Nanning 530004 China; ^2^ Guangxi Engineering and Technology Research Center for High‐Quality Structural Panels from Biomass Wastes Guangxi University Nanning 530004 China; ^3^ State Key Laboratory of Featured Metal Materials and Life‐cycle Safety for Composite Structures Guangxi University Nanning 530004 China; ^4^ Key Laboratory of Disaster Prevention and Structural Safety of Ministry of Education Guangxi University Nanning 530004 China; ^5^ Guangxi Key Laboratory of Disaster Prevention and Engineering Safety Guangxi University Nanning 530004 China; ^6^ College of Chemistry and Chemical Engineering Guangxi University Nanning 530004 China

**Keywords:** human‐machine interactions, ion‐conductivity, liquid‐free ion‐conductive elastomers, mechanical strength, recovery of LiTFSI

## Abstract

Liquid‐free ion‐conductive elastomers with excellent mechanical and electrical conductivity are widely used in flexible sensors, wearable devices, soft touch screens, and supercapacitors. However, the inherent contradiction between mechanical and electrical properties often limits the development of liquid‐free ion‐conductive elastomers. Therefore, the preparation of liquid‐free ion‐conductive elastomers with both high mechanical properties and high ionic conductivity remains a major challenge. In this study, a polyurethane elastomer with multiple crosslinking and a microphase‐separated structures were designed, inspired by the “brick wall” structure of the pearl layer. A polyurethane‐based liquid‐free ion‐conductive elastomer with excellent mechanical strength and outstanding ion‐conducting properties was prepared by introducing lithium bis(trifluoromethane)sulfoximide (LiTFSI) into the polyurethane elastomer. FLICE‐110% liquid‐free ion‐conductive elastomer had excellent mechanical strength (5.46 MPa), exceptional elongation at break (1213%), excellent ionic conductivity (3.29 × 10^−4^ S cm^−1^), and excellent fracture energy (6.25 kJ m^−2^). Flexible sensors prepared based on FLICE‐110% liquid‐free ion‐conductive elastomer have realized applications in wearable devices, multi‐channel strain sensors, and remote‐controlled robots. In addition, we successfully recovered LiTFSI from FLICE‐110% liquid‐free ion‐conductive elastomer. The development of these liquid‐free ion‐conductive elastomers will be expected to show a wide range of applications in flexible sensors, ionic skin, soft robotics, and human‐machine interactions.

## Graphical abstract

⁻Inspired by the “brick wall” structure of pearl layer, FLICE‐110% liquid‐free ionic conductive elastomer had high mechanical strength (5.46 MPa), excellent ionic conductivity (3.29 × 10^−4^ S cm^−1^) and resilience. It can be used in smart music boxes, airplane wings and control manipulators. In addition, a simple method of recovering LiTFSI from FLICE‐110% liquid‐free ion‐conductive elastomer has been adopted.

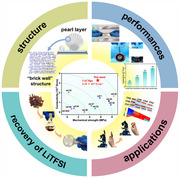

## 1 Introduction

In recent years, with the rapid development of flexible electronic devices, stretchable flexible conductors have been widely used in flexible electronic screens,^[^ ^1^ ^]^ medical monitoring sensors,^[^ ^2^, ^3^ ^]^ soft robots,^[^ ^4^, ^5^ ^]^ and soft‐touch panels.^[^ ^6^, ^7^ ^]^ At present, flexible conductors are mainly divided into flexible electronic conductors and flexible ionic conductors. The former is usually prepared by adding electronically conductive fillers (e.g., carbon nanotubes,^[^ ^8^ ^]^ graphene,^[^ ^9^, ^10^ ^]^ liquid metals^[^ ^11^ ^]^) to polymer substrates. However, these electronically conductive fillers are less compatible with the substrate, and the conductive component tends to separate from the substrate when subjected to large deformations, resulting in performance degradation.^[^ ^12^, ^13^, ^14^ ^]^ In contrast, there is good compatibility between the polymer substrate and the conductive components of certain flexible ionic conductors.^[^ ^15^, ^16^ ^]^ Currently, the research on flexible ionic conductors mainly focuses on hydrogels and ionogels.^[^ ^17^, ^18^ ^]^ Due to the presence of liquid components, ions migrate faster in such substrate, which makes them widely used in flexible electronic devices.^[^ ^19^ ^]^ However, hydrogels are susceptible to water loss during use, leading to unstable performance and limiting their application. Although ionogels effectively inhibit liquid evaporation, their drawback of easy leakage and the toxicity have also seriously affected their application in wearable devices. Therefore, the creation of liquid‐free ionic conductor elastomers with both service stability and biosafety will bring new opportunities for the development of flexible electronic devices.

The current development of liquid‐free ionic conductor elastomers faces the challenge of balancing mechanical properties with ionic conductivity. This is because exceptional mechanical properties require weak chain segment motility of the polymer, and weak chain segment motility leads to a decrease in conductivity. Conversely, the mechanical strength decreases. Therefore, designing liquid‐free ion‐conductive elastomers with high mechanical properties and high ionic conductivity is a great challenge.^[^ ^20^, ^21^, ^22^ ^]^ In recent years, the development of polyurethane‐based liquid‐free ion‐conductive elastomers has offered hope for solving this challenge. Ding et al. designed a novel polyurethane‐based ion‐conductive elastomer based on a phase‐locking strategy with a mechanical strength of 5.0 MPa and an ionic conductivity of 3.77 × 10^−5^ S cm^−1^.^[^ ^19^ ^]^ Wu et al. fabricated a liquid‐free ion‐conductive elastomer with tensile strength of 1.2 MPa and room‐temperature ionic conductivity of 4.02 × 10^−5^ S cm^−1^ by combining a polyurethane elastomer and LiTFSI.^[^ ^23^ ^]^ Yang et al., the polyurethane‐based liquid‐free ion‐conductive elastomer with exhibited excellent mechanical properties of 3.06 MPa and an ionic conductivity of 2.86 × 10^−6^ S cm^−1^.^[^ ^24^ ^]^ Although polyurethane‐based liquid‐free ion‐conductive elastomers have been well developed, the preparation of ion‐conductive elastomers with both high mechanical properties and high ion conductivity remains a long‐term challenge. In addition, ion‐conductive elastomers with excellent fatigue and tear resistance can resist stress damage during use, thereby extending the service life of the elastomer and reducing the frequency of maintenance and replacement costs in actual use, thus promoting the widespread use of flexible elastomers. It is worth noting that the high cost of LiTFSI and the difficulty of decomposition in nature also limit its development. Therefore, the development of a polyurethane‐based liquid‐free ion‐conductive elastomer with excellent mechanical properties, outstanding ion‐conductivity, good fatigue and tear resistance, and recyclability remains an urgent problem.

Normally, the introduction of LiTFSI will destroy the microphase‐separated structures of polyurethane, resulting in a significant decrease in the mechanical properties of the material. Therefore, it is necessary to construct a “hard‐phase enrichment zone” to maintain the microphase‐separated structures of polyurethane‐based liquid‐free ion‐conductive elastomers in the presence of high concentrations of LiTFSI to realize the synthesis of high‐performance liquid‐free ion‐conductive elastomers. In nature, the pearl layer consists of aragonite crystals (calcium carbonate) interspersed with an organic matrix, showing a regular and orderly “brick wall” structure, with more aragonite crystals giving it high hardness and stiffness. The organic matrix is relatively flexible but less strong. When the two are arranged in a “brick wall” structure, the hard and brittle aragonite crystals are distributed like “bricks” in the soft and tough organic matrix, which plays a supporting role. The matrix acts as a cushion, absorbing and dispersing external shocks and preventing crystal breakage. This structure gives the pearl layer excellent mechanical properties, including excellent strength and toughness.^[^ ^25^, ^26^, ^27^ ^]^ Similar to the “brick wall” structure of the pearl layer, the “hard phase‐enriched zone” of the polyurethane elastomer is equiv. to the aragonite of the pearl layer, which improves mechanical properties. The introduction of LiTFSI provides electrical conductivity to the polyurethane elastomer, and this operation is expected to achieve a balance between mechanical properties and electrical conductivity of the liquid‐free ionic conductors.

In this work, inspired by the pearl‐layer structure, a strategy combining multiple cross‐linking and micro‐phase‐separated structures was proposed to achieve efficient aggregation of hard phases in the material and successfully prepare polyurethane‐based elastomers with excellent mechanical properties. By introducing LiTFSI into hard phase‐enriched polyurethane elastomer, a polyurethane‐based liquid‐free ion‐conductive elastomer, FLICE‐110%, was obtained, which combined both high mechanical properties and high electrical conductivity. FLICE‐110% liquid‐free ion‐conductive elastomer exhibited excellent mechanical properties, with mechanical properties of 5.46 MPa, elongation at break of 1 213%, and ionic conductivity at room temperature of 3.29 × 10^−4^ S cm^−1^. In addition, the synergistic effect of multi‐level hydrogen bonding and dynamic imine bonding gave the elastomers excellent energy dissipation capability, resulting in excellent fracture energy (6.25 kJ m^−2^) and resilience. Based on the excellent overall performance of FLICE‐110% liquid‐free ion‐conductive elastomer, applications in wearable devices, multi‐channel strain sensors, and remote‐controlled robots were realized. The development of this material will be expected to provide new insights for further developments in the field of human‐computer interaction.

## 2 Results and Discussion

### 2.1 Molecular Structure Design and Preparation of FLICE‐x% Elastomers

**Figure 1:**
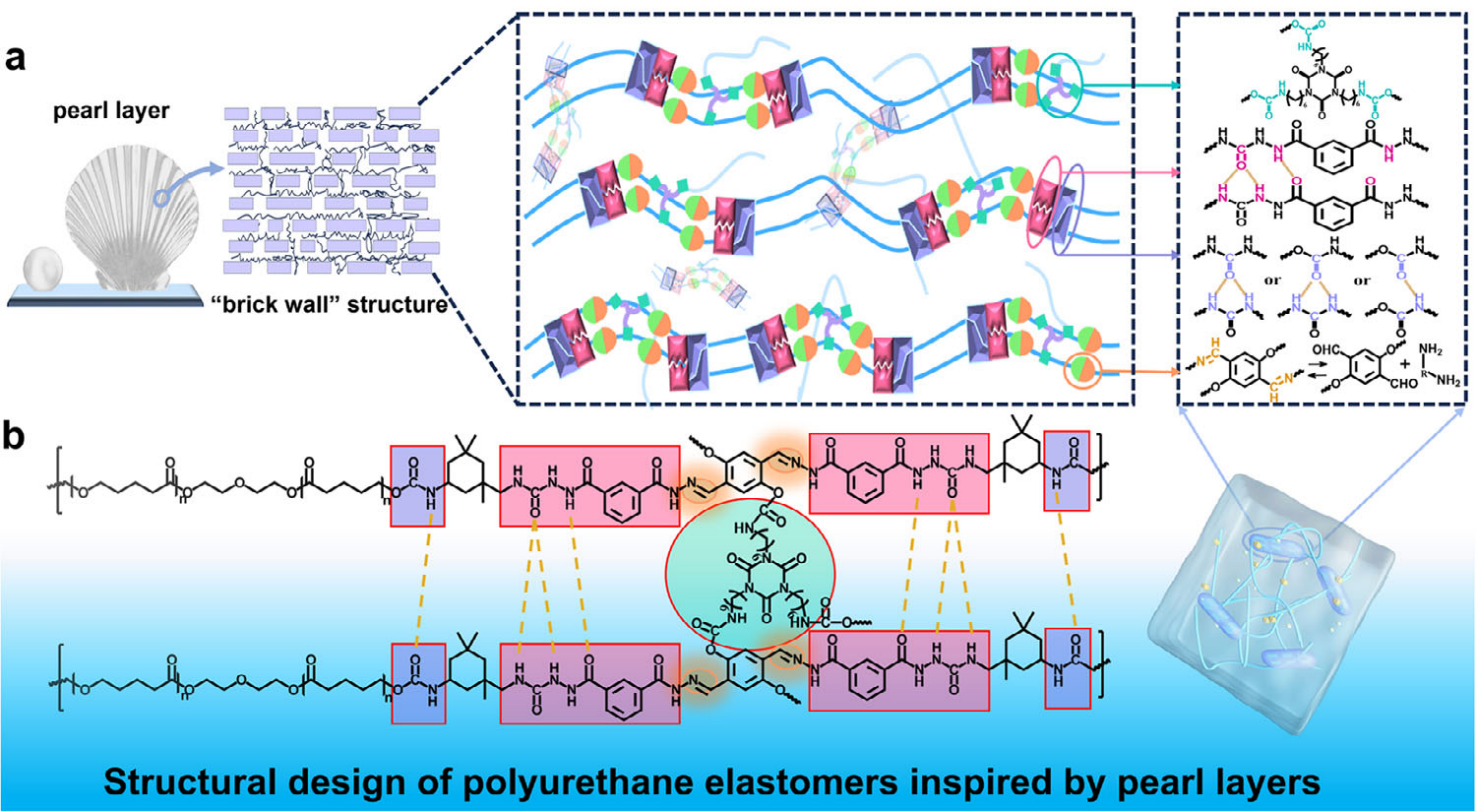
Structural design of polyurethane elastomers inspired by pearl layers. a) Schematic diagram of a structure inspired by a pearl layer “brick‐wall” type structure. b) Structural formula of the prepared FLICE‐x% polyurethane elastomers.

Inspired by the pearl‐layer “brick wall” structure, polyurethane elastomers with high mechanical properties were prepared by combining the strategy of multiple cross‐linking and microphase‐separated structures. By introducing LiTFSI into polyurethane elastomer, a balance between the mechanical properties and ionic conductivity of the target liquid‐free ion‐conductive elastomers was achieved. The schematic of the process inspired by the pearl layer and the structural formula of the prepared elastomer are shown in **Figure** **1**a,b. Polycaprolactone diol (PCL‐2OH) was chosen as the soft chain segment, which was similar to the soft organic matrix of the pearl layer. PCL‐2OH contained a large number of loosely coordinated ether‐oxygen bonds,^[^ ^28^ ^]^ which were able to form lithium‐oxygen coordination bonds with LiTFSI and promote rapid Li^+^ transport in the polymer network, thus improving ionic conductivity of elastomers.^[^ ^23^ ^]^ PCL‐2OH was capped using isoflurane diisocyanate (IPDI), and IPDI was used as a capping agent because its asymmetric structure could disrupt the crystallization of the polymer, transforming the polymer into an amorphous structure and laying the groundwork for the migration of ions at a later stage. In addition, isophthalic dihydrazide (ID) and 2,5 dihydroxyterephthalaldehyde (DHTA) were selected as chain extenders. The hydrazide group on ID reacted with isocyanate to form an acylsemicarbazide (ASCZ), which provided multiple hydrogen bonding sites and contributed to the formation of multilayered hydrogen bonding, thus enhancing the mechanical properties of elastomers. At the same time, the hydrazide group reacted with DHTA to form a dynamic imine bond. And hexamethylene diisocyanate trimer (tri‐HDI) was chosen as the cross‐linking agent. The reaction of tri‐HDI with DHTA gave the target polymers a highly crosslinking network structure, which increased the structure stability of the polymer. The multiple interaction and the cross‐linking sites acted as hard segments of the elastomers, similar to aragonite crystals in the pearl layer, providing excellent mechanical properties of the elastomers. The specific synthesis process of this polyurethane‐based elastomers is shown in Figure S1 (Supporting Information). On this basis, liquid‐free ion‐conductive elastomers, named FLICE‐x%, were synthesized by doping LiTFSI with different mass ratios in the polyurethane matrix. Where x% denotes the mass ratio of LiTFSI to the polymer (i.e., the mass of LiTFSI is x% of the mass of the polymer), and the experimentally designed ratios were 0%, 70%, 90%, 110%, and 130%, which were named FLICE‐0%, FLICE‐70%, FLICE‐90%, FLICE‐110%, and FLICE‐130%, respectively. E.g., FLICE‐70%: LiTFSI mass is 70% of the polymer (0.7:1 mass ratio).

### 2.2 Structural Characterization and Reinforcement Mechanisms of FLICE‐x% Elastomers

**Figure 2:**
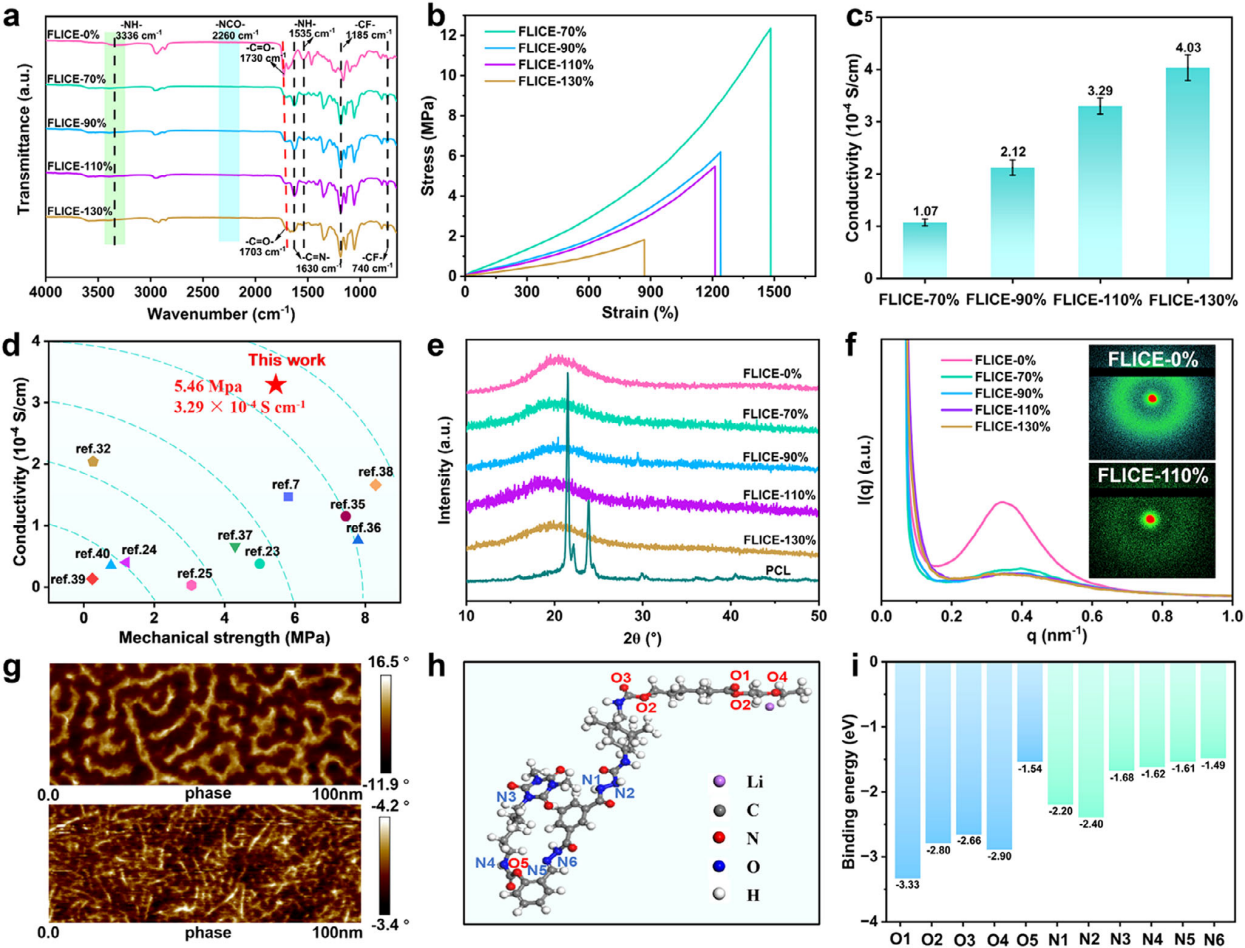
Structural characterization and properties of FLICE‐x% polyurethane elastomers. a) ATR‐FTIR spectra of FLICE‐x%. b) Stress‐strain curves of FLICE‐x%. c) Ionic conductivity of FLICE‐x%. d) Comparison of ionic conductivity and mechanical strength of FLICE‐110% with recently reported ionic conductor materials. e) Wide angle XRD spectrum of FLICE‐x%. f) SAXS spectrum of FLICE‐x%, inset: 2D‐SAXS images of FLICE‐0% and FLICE‐110% samples. g) AFM phase of FLICE‐0% and FLICE‐110% samples. h) Snapshots of FLICE‐x% liquid‐free ion‐conductive elastomers structures for MD simulations. i) Adsorption binding energy of lithium ions at N and O sites of different configurations in the molecular chain.

The successful synthesis of FLICE‐x% elastomers was confirmed by attenuated total reflectance fourier transform infrared spectroscopy (ATR‐FTIR) (**Figure** **2**a). The ‐NH‐ stretching vibration at 3 336 cm^−1^ and the ‐NH‐ bending vibration peak at 1 535 cm^−1^ were attributed to the ‐NH‐ group in the carbamate.^[^ ^23^, ^29^ ^]^ In addition, the disappearance of the ‐NCO‐ peak at 2 260 cm^−1^ indicated that the IPDI had fully reacted.^[^ ^30^ ^]^ The ‐C = N‐ stretching vibration at 1 630 cm^−1^ corresponded to the dynamic imine bonding generated by the dehydration reaction of the aldehyde group and the amino group.^[^ ^31^ ^]^ Compared with the FLICE‐0% elastomers, the LiTFSI doped liquid‐free ion‐conductive elastomers both showed peaks at 1 185 and 740 cm^−1^, which were not found in the FLICE‐0% elastomers, and these two peaks were attributed to ‐C‐F‐ bonding, indicating that LiTFSI was successfully doped into polyurethane elastomers.^[^ ^23^ ^]^ In addition, the stretching vibration peak at 1 730 cm^−1^ was attributed to the ‐C = O in the urethane group and ASCZ group, and the stretching vibration peak at 1 730 cm^−1^ tended to move to a lower wavelength as the content of LiTFSI increased, and shifted from 1 730 to 1 703 cm^−1^. This was attributed to the coordination between the ‐C = O group and Li^+^ in the polyurethane, which also indicated that LiTFSI was successfully incorporated into polyurethane elastomers.^[^ ^23^ ^]^ The energy dispersive X‐ray spectrogram showed the uniform distribution of LiTFSI in the polyurethane network (Figure S2, Supporting Information). Taking FLICE‐110% liquid‐free ion‐conductive elastomer as an example, the elements C, N, O, as well as F and S in FLICE‐110% liquid‐free ion‐conductive elastomer were uniformly distributed in the elastomer, which contributed to the formation of homogeneous and continuous ion‐conductive channels in the elastomer.^[^ ^32^, ^33^ ^]^

To investigate the mechanical and conductive properties of the synthesized FLICE‐x% elastomers, the mechanical and conductive properties were evaluated by uniaxial tensile and electrochemical experiments. As shown in Figure 2b, the uniaxial tensile experiments were carried out with a strain rate of 50 mm min^−1^ at room temperature, and the results showed that all the samples did not yield during the tensile process, and FLICE‐x% elastomers exhibited a typical elastic behavior. The fracture strength of FLICE‐x% elastomers gradually decreased with increasing LiTFSI content, while the conductivity increased accordingly (Figure 2c). In particular, the FLICE‐0% elastomer had a breaking strength of 74.30 MPa, an elongation of 789%, and 0.14 g of FLICE‐0% elastomer was able to pull up a weight of more than ≈35 000 times its weight (Figure S3, Supporting Information). When the LiTFSI content was increased from 70% to 130%, the fracture strength of the liquid‐free ion‐conductive elastomers gradually decreased from 12.35 to 1.81 MPa. The ionic conductivity, on the other hand, increased from 1.07 × 10^−4^ S cm^−1^ to 4.03 × 10^−4^ S cm^−1^, and the impedance spectrum was shown in Figure S4 (Supporting Information). Compared with previous studies, FLICE‐110% liquid‐free ion‐conductive elastomer had a breaking strength of 5.46 MPa and a conductivity of 3.29 × 10^−4^ S cm^−1^, which showed a good overall performance advantage in terms of ionic conductivity and breaking strength (Figure 2d).^[^ ^7^, ^19^, ^23^, ^24^, ^31^, ^34^, ^35^, ^36^, ^37^, ^38^, ^39^ ^]^ And 0.095 g of FLICE‐110% liquid‐free ionic conductive elastomer can still easily pull up a 1 kg weight, 10 000 times its own weight, as shown in Figure S5 (Supporting Information). In order to investigate the reasons for the changes in the properties of these elastomers, the microstructure of the elastomers was investigated by wide angle X‐ray diffraction (XRD) analyses (Figure 2e). PCL‐2OH showed sharp crystalline peaks at 2*θ* = 22 and 24°. However, the carefully prepared polyurethanes as well as polyurethane‐based liquid‐free ion‐conductive elastomers showed only a typical amorphous peak at 2*θ* = 20°, indicating that the synthesized samples were amorphous polymers. This was due to the asymmetric structure of IPDI hindered the crystallization of the polymer chain segments and the amorphous structure facilitated the rapid migration of ions between the polymer chains.^[^ ^23^ ^]^ The structural changes induced by the increase of LiTFSI content were further investigated by small‐angle X‐ray scattering (SAXS) tests, as shown in Figure 2f. Both FLICE‐0% and FLICE‐110% showed scattering circles, indicating that both FLICE‐0% and FLICE‐110% had microphase‐separated structures. With the increase of LiTFSI content, the scattering rings of the elastomers gradually weakened, which was mainly because the large TFSI^−^ group of LiTFSI interfered with the stacking of the molecular chains and hindered the aggregation of the hard‐phase domain structure. When the LiTFSI content was 130%, the FLICE‐130% elastomer still had microphase‐separated structures, which provided for the maintenance of high mechanical strength. The high LiTFSI concentration and microphase‐separated structures provided FLICE‐x% liquid‐free ion‐conductive elastomers with high ionic conductivity and excellent fracture strength.^[^ ^40^, ^41^ ^]^ FLICE‐0% and FLICE‐110% liquid‐free ion‐conductive elastomers were used as examples to further verify the phase separation enhancement mechanism by atomic force microscopy (AFM). In the image of Figure 2g, dark and bright regions coexisted, with dark regions representing soft domains and bright regions representing hard domains. It was noteworthy that the AFM phase images of FLICE‐110% liquid‐free ion‐conductive elastomer had a microphase‐separated structure despite the LiTFSI content of 110%, which was consistent with the SAXS results. The hard domain structure was partially preserved, thus ensuring that FLICE‐110% liquid‐free ion‐conductive elastomer contained high mechanical properties. At the same time, the partial destruction of the hard domains enhanced the activity of the molecular chains and promoted the migration of Li^+^, which leaded to an increase in the ionic conductivity.^[^ ^42^ ^]^

The movement and stability of the chain segments in FLICE‐x% were further explored by dynamic thermo‐mechanical analysis (DMA) tests. The energy storage modulus of the FLICE‐x% elastomers was larger than the loss modulus in the range of − 70–100 °C, reflecting that the FLICE‐x% elastomers exhibited good elastic behavior over a wide temperature range, with the potential to work in an increasingly wide temperature window (Figure S6a–e, Supporting Information). In addition, the glass transition temperature (*T*_g_) of FLICE‐x% elastomers gradually increased with the increase of LiTFSI content (Figure S6f, Supporting Information), which indicated the formation of ligand interactions between Li^+^ and the carbonyl and ether oxygens in PCL‐2OH, thus restricting the movement of the soft chain segments. At the same time, the loosely coordinating O‐Li^+^ interaction and lower activation energy for ion transport can contribute to higher ion conductivity, and the migration mechanism was shown in Figure S7 (Supporting Information).^[^ ^19^ ^]^ Under external stimulation, multiple multilevel hydrogen bonds were continuously and repeatedly broken and reorganized, providing more flexible chain segments for Li^+^ migration and facilitating the increase of ionic conductivity. Subsequently, theoretical calculations further explained the high electrical conductivity. Snapshots of all‐atom molecular dynamics (MD) simulations of the structure of FLICE‐110% liquid‐free ion‐conductive elastomer were shown in Figure 2h and Figure S8 (Supporting Information). The corresponding data in Figure 2i showed that both O and N atoms adsorbed lithium ions excellently, and ion transport can occur between the N and O sites, which promoted the migration of ions and thus increased ionic conductivity.^[^ ^31^ ^]^ In addition, the liquid‐free ion‐conductive elastomers exhibited excellent thermal stability due to the outstanding thermal stability of LiTFSI and the stable 3D network structure in the polymer. The thermogravimetric analysis results (Figure S9, Supporting Information) showed that the decomposition temperatures of FLICE‐x% elastomers were all higher than 120 °C at a mass loss of 5%, confirming that FLICE‐x% elastomers had a wide working temperature range.

### 2.3 FLICE‐110% Liquid‐Free Ion‐Conductive Elastomer with Excellent Resilience, Tar Resistance, Impact Resistance, Biocompatibility and Antimicrobial Properties

**Figure 3:**
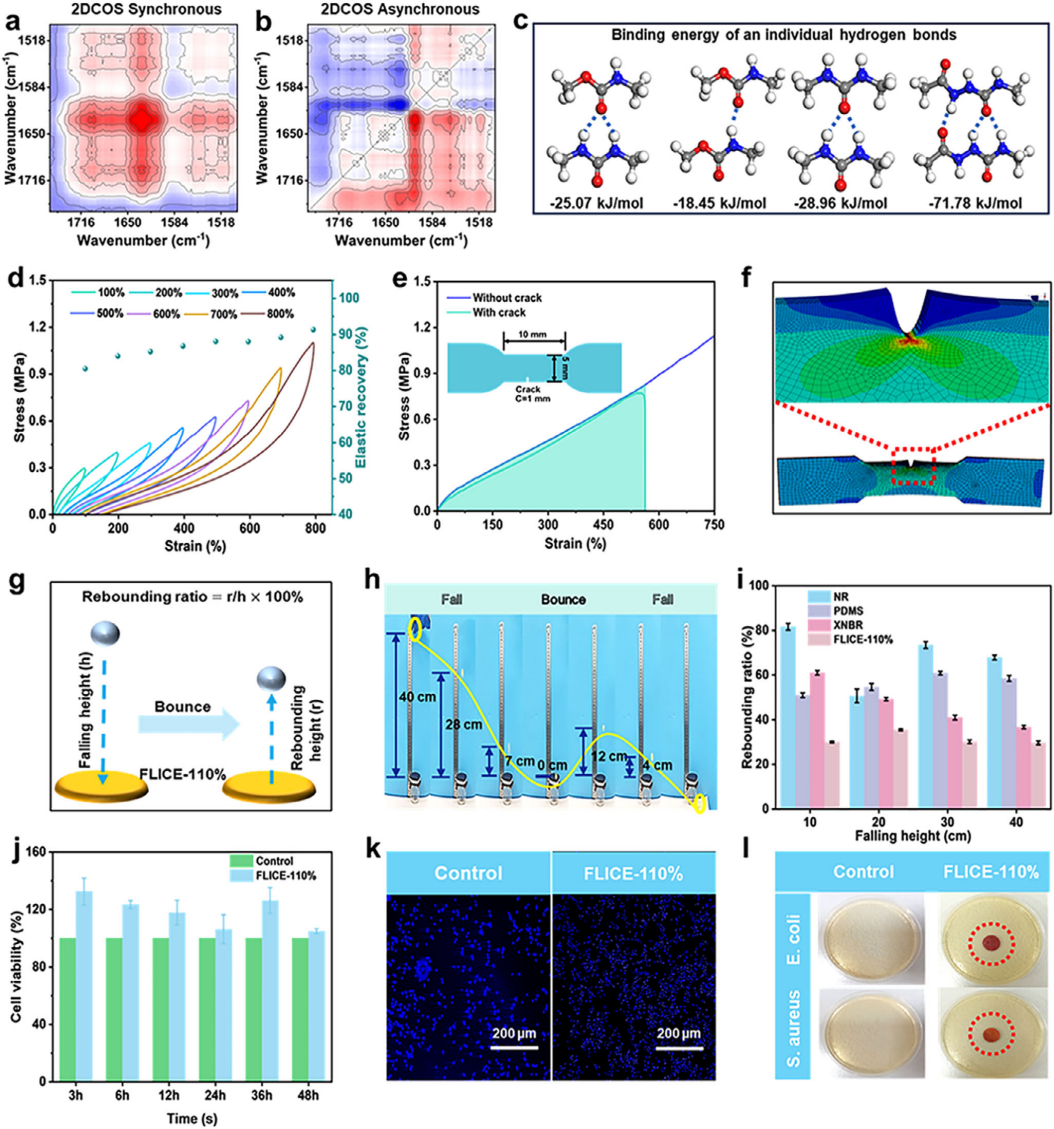
FLICE‐110% liquid‐free ion‐conductive elastomer with excellent resilience, tear resistance, impact resistance, biocompatibility and antibacterial properties. a, b) Synchronous and asynchronous spectra of 2DCOS generated by the temperature‐variable IR spectra tests. c) Bonding energy of four independent hydrogen bonds. d) Continuous cycle tensile curves and ER rates of FLICE‐110% at different strains (100%–800%). e) Stress‐strain curves for FLICE‐110% elastomer samples without and with notches. f) Finite element simulation of the tear resistance test. g) Schematic diagram of the safety cushion drop ball experiment based on FLICE‐110% elastomer. h) Demonstration of the actual course of the experiment with a glass ball falling on a FLICE‐110% elastomer. i) Rebound rate of a glass bead falling on NR, PDMS, XNBR, and FLICE‐110% elastomer. j) Cell viability CCK‐8 assay of L929 cells cultured with extracts of FLICE‐110% samples for different culture times. Cells cultured with a complete growth medium were used as control. k) Live/dead staining of L929 cells cultured with blank control and FLICE‐110% extract for 48 h. Scale bar: 200 µm. (l) The antibacterial effect of FLICE‐110% film sample against E. coli and S. aureus.

FLICE‐110% liquid‐free ion‐conductive elastomer, constructed from multiple interacting exhibited not only excellent fracture strength and ionic conductivity, but also remarkable resilience, fatigue resistance, and reparability. In order to investigate the energy dissipation capability of FLICE‐110% liquid‐free ion‐conducting elastomers, temperature‐variable IR spectra tests and density‐functional theory (DFT) calculations were performed. The results of the 2D‐COS synchronous and asynchronous FTIR spectroscopy and the variable‐temperature infrared tests were displayed in **Figures** **3**a,b and S10 (Supporting Information). The stretching vibrational bands of ‐NH‐ near 1 545 cm^−1^ were blue‐shifted and decreased in intensity as the temperature increased from 35 °C to 130 °C. Meanwhile, the stretching vibration band of ‐C = O appearing near 1 703 cm^−1^ showed a similar trend, which suggested that the breakage of hydrogen bonds and their re‐formation triggered different types of hydrogen bonding changes, and the existence of multiple types of hydrogen bonding provided an important guarantee of energy dissipation in elastomers. In addition to the variation in the types of hydrogen bonds, DFT also revealed different binding energy between the four types of hydrogen bonds (Figure 3c), suggesting the presence of hydrogen bonds with different strengths. The weaker hydrogen bonds contributed to rapid reconfiguration and dissipation of energy upon rupture, while stronger bonds contributed to the construction of a strong molecular network, which provided the basis for excellent resilience and fatigue resistance.^[^ ^43^ ^]^ In order to further strengthen the correlation between the microscopic behavior and macroscopic properties of hydrogen bonding, systematic elucidation of the mechanism of hydrogen bonding in material systems was being pursued. The hydrogen bond formation mechanisms of both urethane and acylurea in this research work originate from electrostatic interactions of polar groups in their molecular structures, but the exact mechanisms vary depending on the structural differences. In urethane (R‐O‐C(=O)‐NH‐R′), the ‐NH‐ of the urea group acts as a hydrogen bond donor to form a hydrogen bond with the ‐C = O of an adjacent molecular chain. Among them, ‐NH‐C = O has a higher hydrogen bond strength (bond energy ≈18–26 kJ mol^−1^), a shorter bond length, and is directional (X‐H…O bond angle close to 180°). These hydrogen bonds form a 1D linear or laminar arrangement along the main chain, imparting rigidity and crystallinity to the material. Acylaminoureas (R‐C(=O)‐NH‐C(=O)‐NH‐R′) can form a 3D crosslinked hydrogen‐bonding network due to the presence of a dual NH hydrogen‐bond donor and a dual C = O hydrogen‐bond acceptor. Each ‐NH‐ can form a hydrogen bond with the neighboring ‐C = O (bond energy ≈27–72 kJ mol^−1^), and the synergistic effect of the double‐hydrogen bond significantly improves the stability of the bond.^[^ ^44^, ^45^ ^]^ This high‐density hydrogen bonding network not only enhances the mechanical strength of the material, but also confers self‐healing capabilities through dynamic hydrogen bonding breakage‐reorganization. E.g., during stretching, some of the hydrogen bonds preferentially break to dissipate energy, while the unbroken hydrogen bonds maintain the network integrity, allowing the material to recover its properties through hydrogen bond reorganization at room temperature. The synergistic design of strong and weak hydrogen bonds builds a multiple hydrogen bonding system. The strong hydrogen bonds of acyl‐ureas act as rigid cross‐linking points to provide structural stability, while the weak hydrogen bonds of carbamates act as dynamic units to enhance energy dissipation and self‐healing efficiency.^[^ ^42^, ^46^, ^47^, ^48^, ^49^ ^]^ The multiple hydrogen bonding synergies give FLICE‐110% liquid‐free ion‐conductive elastomer excellent resilience, tear resistance, impact resistance, and reparability. In order to further investigate its energy dissipation capability, cyclic tensile experiments were conducted on FLICE‐110% liquid‐free ion‐conductive elastomer under different strains, as shown in Figure 3d. The hysteresis loop area gradually increased with increasing strain, which indicated that the dissociation and reorganization of multi‐level hydrogen and dynamic imine bonds and lithium‐oxygen bonds effectively achieved energy dissipation during high strain stretching. Meanwhile, the elastic recovery (ER) also increased with the increase of strain up to 91.31%. In addition, Figure S11 (Supporting Information) showed that the rectangular sample (30 mm × 6 mm × 0.5 mm) of FLICE‐110% liquid‐free ion‐conductive elastomer, after being stretched to 3 times the original length and kept in the stretched state for 10 s, was able to recover to the original length after being placed at room temperature for 10 min, confirming its good resilience. We evaluated the fatigue resistance of FLICE‐110% by successive loading‐unloading cycles (Figures S12, S13, Supporting Information). The results showed that the hysteresis loop area was the largest in the initial cycle and then decreased gradually, while the hysteresis loop area was similar from the second to the fifth cycle. The dissipated energy of initial cycle was as high as 0.155 MJ m^−3^, which was due to that a large amount of energy was dissipated by the dynamic bond opening in the first cycle. After several cycles, the dynamic bonds in the network were gradually reorganized. When the FLICE‐110% liquid‐free ion‐conductive elastomer was rested for 15 min on the hot bench and then stretched again, the hysteresis loop was almost restored to the initial state, and the dissipated energy was close to the initial state, reaching 0.126 MJ m^−3^. This was due to the rapid reorganization of the dynamic bonds under the resting and heating conditions, which showed good resilience.

Excellent energy dissipation capability offered FLICE‐110% liquid‐free ion‐conductive elastomer excellent resilience and also increased its tear resistance. The excellent tear resistance can effectively mitigate microcracks that may occur during repeated use, thereby extending service life. To investigate the tear resistance of FLICE‐110% liquid‐free ion‐conductive elastomer, its fracture energy through uniaxial tensile tests was evaluated. As shown in Figure 3e, the sample was cut into a dumbbell shape of 10 mm × 5 mm × 0.6 mm, and a 1 mm notch was cut in the middle of the sample. By testing its tensile properties, the results showed that the sample had a fracture energy of 6.25 kJ m^−2^ and can withstand a weight of two thousand times its self‐weight without tearing in the presence of a notch (Figure S14, Supporting Information), indicating good fracture toughness and notch insensitivity. In order to investigate the crack propagation resistance in FLICE‐110% liquid‐free ion‐conductive elastomer, the force distribution of the samples during stretching was analyzed by using finite element simulations (Figure 3f). The results showed that as the tensile strain increased, the dynamically hard structural domains in the polymer network achieved crack bridging through molecular chain entanglement and sliding. This effectively transferred the stress concentrated at the crack tip to the entire polymer network, thereby preventing lateral extension of the crack tip. In addition, the sliding of molecular chains and the breaking of hydrogen bonds consumed a large amount of energy.^[^ ^30^, ^50^ ^]^ FLICE‐110% liquid‐free ion‐conductive elastomer exhibited excellent tear resistance due to a combination of multiple interacting and microphase‐separated structures. In addition, Figure S15 (Supporting Information) demonstrates the outstanding puncture resistance of FLICE‐110% liquid‐free ion‐conductive elastomer. To more accurately evaluate the puncture resistance of FLICE‐110%, we performed needle impact testing using a steel needle to puncture a 677 µm thick film to evaluate the puncture resistance of the elastomer (Figure S16, Supporting Information). From the force‐displacement curve in Figure S16 (Supporting Information), it can be seen that when the film is completely punctured at a maximum displacement of 17.325 mm, the FLICE‐110% liquid‐free ion‐conductive elastomer can withstand a puncture force of 3.845 N, with a puncture resistance of 5.680 N mm^−1^. In comparison, polydimethylsiloxane (PDMS) has a puncture resistance of 1.611 N mm^−1^. Experimental data indicate that the FLICE‐110% liquid‐free ion‐conductive elastomer exhibits excellent puncture resistance, with a puncture strength 3.5 times higher than that of PDMS. The excellent energy dissipation capability of FLICE‐110% liquid‐free ion‐conductive elastomer was expected to enable the application in safety cushions (Figure 3g,h). The FLICE‐110% liquid‐free ion‐conductive elastomer was used as a safety cushion to test the rebound height (r) of a 4.8 g glass ball at different heights (h), defining r/h as the rebound rate. Natural rubber (NR) with covalently cross‐linked network, polydimethylsiloxane (PDMS) with van der Waals' forces, and carboxy nitrile butadiene rubber (XNBR) with hydrogen bonding were selected as the comparative materials to evaluate the cushioning properties of FLICE‐110% liquid‐free ion‐conductive elastomer. The test results showed that at the same landing height, the rebound height of FLICE‐110% liquid‐free ion‐conductive elastomer was significantly lower than that of the other three materials, and its cushion ratio was also the smallest (Figure 3i). E.g., at a landing height of 40 cm, the rebound ratios of glass ball on NR, PDMS, and XNBR cushions were 67.5%, 57.5%, and 37.5%, respectively, while the rebound ratio on FLICE‐110% liquid‐free ion‐conductive elastomer was only 30%. This was due to the excellent energy dissipation ability of FLICE‐110% liquid‐free ion‐conductive elastomer. When the elastomer was impacted by a glass ball, the breakage and reorganization of multiple dynamic bonds in the network of FLICE‐110% liquid‐free ion‐conductive elastomer effectively absorbed the impact force and demonstrated excellent impact resistance.^[^ ^51^ ^]^ Therefore, FLICE‐110% liquid‐free ion‐conductive elastomer provided a new idea for the preparation of high‐performance damping protection devices as ideal safety cushioning materials. Based on the fact that FLICE‐110% liquid‐free ion‐conductive elastomers are rich in multilevel hydrogen bonding and dynamic imine bonding, this lays the foundation for FLICE‐110% liquid‐free ion‐conductive elastomers to have excellent self‐healing capabilities. In order to systematically analyze the self‐healing behavior, self‐healing experiments were conducted on FLICE‐110% liquid‐free ion‐conductive elastomers by making a scratch with a scalpel, placing a drop of solution on the scratch, and then placing it on a hot bench at 130 °C. The results of the self‐healing experiments are shown in Figure S17 (Supporting Information). Microscopic observation of the healing results showed that the scratch almost completely disappeared after 48 h of heating at 130 °C. Next, the recovery of mechanical properties and ionic conductivity of FLICE‐110% liquid‐free ionic conductive elastomers were tested, and the experimental results are shown in Figures S18, S19 (Supporting Information). After 32 h of self‐healing, the tensile strength of the material can be recovered to 64% of the initial state, and its recovery rate is further increased to 80% after 48 h, indicating that the material has a high self‐healing efficiency. In addition, we conducted ionic conductivity tests on the self‐healed FLICE‐110%, and the results indicated that the conductivity of the healed FLICE‐110% liquid‐free ionic conductive elastomers is not significantly different from the original one, and the recovery efficiency reaches 90%. Therefore, FLICE‐110% has a broad application prospect in the fields of flexible sensors and wearable electronic devices.

Since wearable devices are commonly used for human health monitoring and need to be in contact with human skin for a long period, elastomers are required to have excellent biocompatibility. The CCK‐8 test method further evaluated the biocompatibility of FLICE‐110% liquid‐free ion‐conductive elastomer by measuring the viability of mouse fibroblasts (L929). The results showed that after 48 h, the cell viability in the medium containing FLICE‐110% liquid‐free ionic conductive elastomer extract was still above 90% (Figure 3j). In general, cell survival above 80% is considered to be well biocompatible. Therefore, the cell survival of FLICE‐110% liquid‐free ion‐conducting elastomers is higher than 100%, indicating that L929 cells have a good ability to grow. We believe that this phenomenon shows that FLICE‐110% liquid‐free ion‐conductive elastomer provides favorable attachment conditions for the cells at the initial stage, which promotes the activity and proliferation of the cells, thus appearing at a higher survival rate.^[^ ^52^, ^53^, ^54^ ^]^ Meanwhile, Figure 3k shows the live/dead staining of L929 cells cultured in blank control and FLICE‐110% liquid‐free ion‐conductive elastomer extract for 48 h. There was no significant difference between the two, which demonstrated its good biocompatibility. When conducting human health monitoring, we sometimes encounter human sweating. To evaluate the stability of FLICE‐110% liquid‐free ionic conductive elastomer in sweat environment, we simulated human sweat environment on a pig skin model and observed it for a long period of time. The experimental results showed that the material did not cause any damage or adverse reaction to the pig skin tissue during the long testing process, and no inflammation or skin barrier disruption was observed. This indicates that FLICE‐110% liquid‐free ionic conductive elastomer has good biosafety, and the test results are shown in Figure S20 (Supporting Information). To evaluate the feasibility of FLICE‐110% liquid‐free ion‐conductive elastomer for wearable applications, we examined its mass change, mechanical properties, and conductivity after exposure to a simulated sweat environment for 1, 6, and 12 h (Figures S21–S23, Supporting Information). The data show that when in a sweat environment, there is a slight elevation in its mass due to the presence of moisture, resulting in a slight increase in conductivity and a slight decrease in mechanical properties. When the sensors were exposed to artificial sweat, their performance remained stable with no significant degradation in sensing performance compared to dry conditions (Figure S24, Supporting Information). In summary, these slight variations do not affect its use in wearable devices, i.e., FLICE‐110% liquid‐free ion‐conductive elastomer has good feasibility as a wearable device. In addition, after drying them, it was found that there was no significant loss of quality even after prolonged exposure to sweat (Figure S25, Supporting Information). This indicates that the amount of LiTFSI precipitated when the human body comes into contact with sweat is very small, and relevant studies have shown that a light concentration of LiTFSI is not harmful to the human body.^[^ ^55^ ^]^ In addition to this, even though there is a very small risk, in order to reduce the risk even further, FLICE‐110% liquid‐free ion‐conductive elastomer was encapsulated using polydimethylsiloxane (PDMS). Sensing tests were conducted on the FLICE‐110% liquid‐free ion‐conductive elastomer before and after encapsulation, and the results showed no significant difference in the sensing signals, indicating that the encapsulation did not affect its sensing and monitoring performance (Figure S26, Supporting Information). This further validates the feasibility of FLICE‐110% liquid‐free ion‐conductive elastomer as a wearable device. These research results provide important support for the further optimization and practical application of FLICE‐110% and also lay a solid foundation for its promotion in the fields of smart medicine and sports monitoring. In addition, FLICE‐110% liquid‐free ionic conductive elastomer was placed in Staphylococcus aureus (S. aureus) and Escherichia coli (E. coli) Petri dishes for 24 h. Obvious inhibitory circles were observed around it, indicating that the ionic conductive elastomers could effectively prevent bacterial growth and had an excellent antibacterial effect (Figure 3l). The antibacterial mechanism of ion‐conductive elastomers can effectively prevent the growth of bacteria. The antibacterial mechanism of ion‐conductive elastomers is that when the bacterial suspension containing Escherichia coli or Staphylococcus aureus is uniformly distributed above the surface of the film, lithium ions can disrupt the integrity of the bacterial cell membrane function, affecting the metabolic process of the bacteria, leading to the death of the surrounding bacteria, and therefore forming an inhibitory bacterial circle with good antibacterial effects.^[^ ^56^, ^57^ ^]^ Furthermore, FLICE‐x% elastomers exhibited good environmental stability. As an example, FLICE‐110% liquid‐free ion‐conductive elastomers (Figure S27, Supporting Information) were exposed to air for 30 consecutive days without significant fluctuations in conductivity, which provided a guarantee for long‐term use in the later stage. The unique defect‐insensitive response, great resilience, excellent biocompatibility, antibacterial properties, and environmental stability made the FLICE‐110% liquid‐free ionic conductive elastomer promising for applications in wearable devices.

### 2.4 Wearable Sensors Prepared Based on FLICE‐110% Liquid‐Free Ion‐Conductive Elastomer

**Figure 4:**
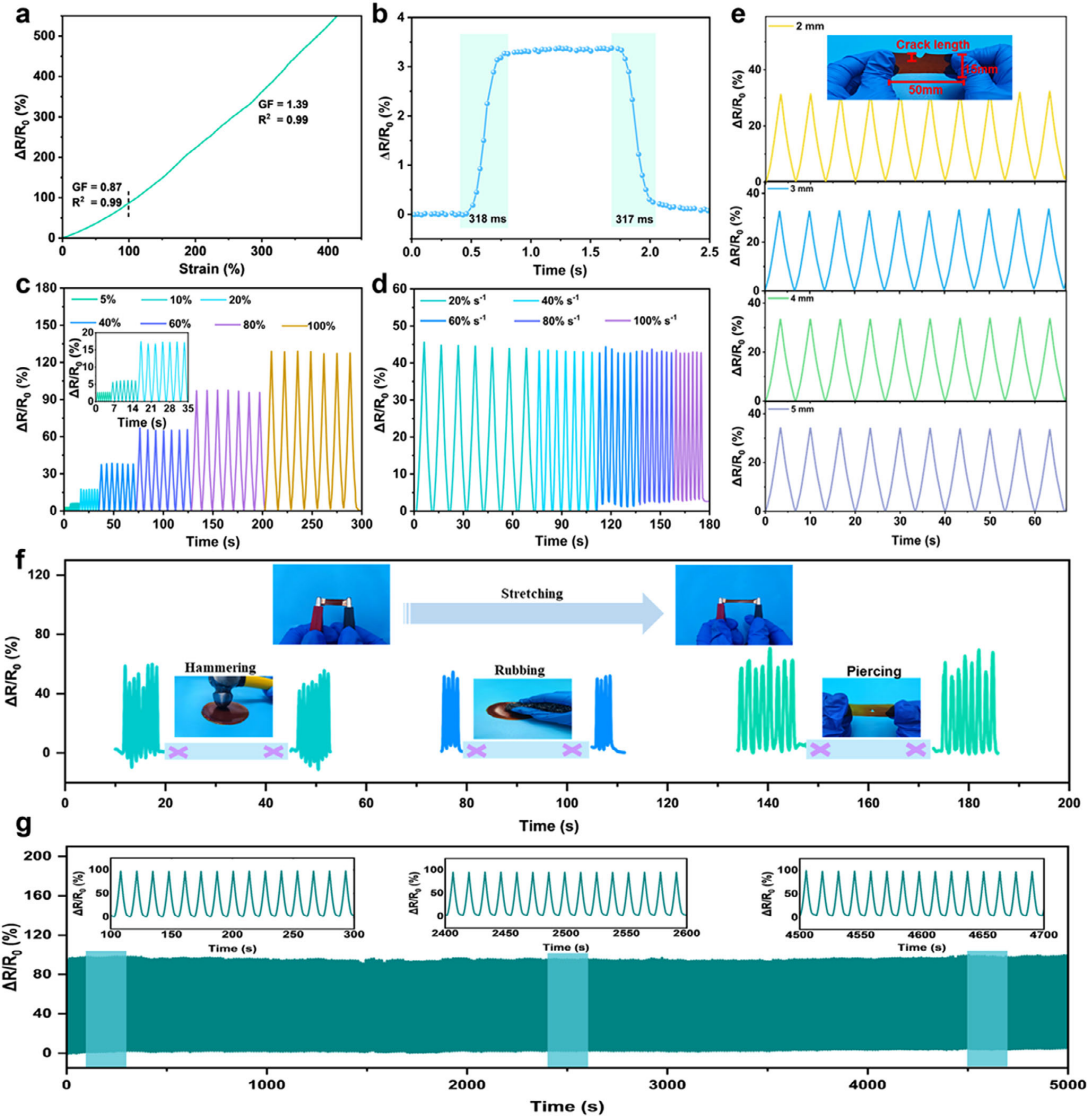
Strain sensing performance based on FLICE‐110% liquid‐free ion‐conductive elastomer. a) Sensitivity test results of FLICE‐110% liquid‐free ion‐conductive elastomer. b) Response time of FLICE‐110% liquid‐free ionic conductive elastomer when stretched at 5% strain. c) FLICE‐110% liquid‐free ionic conductive elastomer relative resistance changes of sensor at various strain (5%–100%) for the same strain rate. Inset: FLICE‐110% liquid‐free ionic conductive elastomer sensor relative resistance changes at 5%–20% strain. d) Relative resistance changes of FLICE‐110% liquid‐free ion‐conductive elastomer at the same strain (100%) but at different strain rates of the sensor. e) FLICE‐110% liquid‐free ion‐conductive elastomer at the same strain (100%) with different notch lengths. f) Relative resistance changes of sensors based on FLICE‐110% liquid‐free ion‐conductive elastomer when stretched before and after hammering, rubbing, and piercing, respectively. g) Relative resistance changes of FLICE‐110% liquid‐free ion‐conductive elastomer sensors repeatedly stretched at 100% strain and cycled for 5 000 s.

FLICE‐110% liquid‐free ionic conductive elastomer laid a solid foundation for the construction of high‐performance flexible sensors by their excellent mechanical properties, electrical conductivity, defect‐insensitive response, biocompatibility, and environmental stability. The FLICE‐110% liquid‐free ion‐conductive elastomer was employed to construct a resistive strain sensor. Sensitivity was recognized as a critical performance indicator for ionic skin, with the gauge factor (GF) serving as a quantitative measure of strain sensor sensitivity. The measured GF value of FLICE‐110% liquid‐free ion‐conductive elastomer was 0.87 in the strain range of 0%–100%, while the GF value was elevated to 1.39 when the strain was increased to 100%–400% (**Figure** **4**a), which indicated that the elastomer had excellent strain sensitivity. In addition, sensors based on FLICE‐110% liquid‐free ion‐conductive elastomer had a very fast response time, with a response time of 318 ms in tension and 317 ms in recovery (Figure 4b), which enabled real‐time monitoring of the human body's movement status. The FLICE‐110% liquid‐free ion‐conductive elastomer can not only monitor large strains (40%–100%), but also identify‌‌ fine strains, and was able to exhibit excellent sensitivity and stability even when the rate of change was 5%–20% for small strains (Figure 4c). To further verify its stability, we tested the relative resistance changes of FLICE‐110% resistive sensors under 100% strain with different strain rates (Figure 4d), and the results showed that the tensile rate had almost no effect on its sensing performance. In addition, we evaluated the strain‐sensing performance of the elastomer in the presence of a notch by cutting different lengths of notch in the FLICE‐110% liquid‐free ion‐conductive elastomer. Figure 4e shows that ΔR/R_0_ remained almost the same at 100% strain, demonstrating that notched strain sensors with different notch lengths all had reliable sensing signal output.^[^ ^58^ ^]^ We note that the strain sensing signals were tested at a tensile strain of 100% in the presence of FLICE‐110% liquid‐free ion‐conductive elastomer carrier notches. As shown in Figure 4e, the tear‐resistant tensile results indicate that FLICE‐110% liquid‐free ion‐conductive elastomer exhibits good notch insensitivity up to a tensile strain of 560%. Consequently, these notches show no significant impact on the material's sensing performance. Second, the notch induces localized strain concentration in the elastomer. However, owing to the material's inherent flexibility and covalent crosslinked network, the strain distributes over a broader region, maintaining a relatively uniform overall strain field. Consequently, despite variations in notch length, the strain distribution remains largely unchanged, leading to consistent signal output in sensing measurements. In addition, the detection of the sensing signals mainly relies on the overall conductive network of the material rather than the microscopic strain changes in the local region. The signal reflects more the global deformation properties of the material, while the change of notch fails to significantly affect the adjustment of the overall conductive path. As a result, FLICE‐110% liquid‐free ion‐conductive elastomers have good strain‐sensing properties in the presence of notches. FLICE‐110% liquid‐free ion‐conductive elastomer is made into sensors to monitor various movements of the human body, as shown in Figure S28 (Supporting Information). The sensors were affixed to finger joints at flexion angles of 30°, 60°, and 90°, respectively, and the results indicated a positive correlation between flexion angle and relative resistance. The degree of deformation significantly affected the change in resistance, so the degree of deformation can be differentiated according to the magnitude of the change in resistance. This phenomenon was attributed to the Poisson effect: when an elastomer was stretched, the ion channels contracted to varying degrees and the resistance increased.^[^ ^23^, ^58^, ^59^ ^]^ When the finger bending angle was kept constant, the electrical signals showed a plateau state and the ∆R/R_0_ values were basically unchanged, which proved that FLICE‐110% liquid‐free ion‐conductive elastomer had a combination of dynamic sensing and static stability, which was important for human motion monitoring. In addition, when the fingers were bent at the same angle but with different bending speeds, the electrical signals indicated that the stretching rate had no effect on their sensing performance (Figure S29, Supporting Information), which was consistent with the results in Figure 4d. In addition to this, different joint parts of the body can be monitored (Figure S30, Supporting Information), such as fingers, wrists, elbows, ankles, knees and shoulders. It was important to note that liquid‐free ion‐conductive elastomer was inevitably exposed to complicated environmental, multiple bending and repeated stretching during actual use. Assessing the durability of elastomer was therefore of paramount importance. Comparison of the relative resistance changes of FLICE‐110% liquid‐free ion‐conductive elastomer before and after experiencing hammering, rubbing, and piercing showed that the sensors had excellent sensing stability (Figure 4f). Such an excellent sensing stability was due to the excellent mechanical properties of FLICE‐110% liquid‐free ion‐conductive elastomer, as well as its outstanding notch insensitivity, which made it more resistant to external stimuli. At the same time, FLICE‐110% liquid‐free ion‐conductive elastomer was repeatedly cyclically stretched up to 350 cycles at a strain of 100% for 5 000 s (Figure 4g). The relative electrical resistance of FLICE‐110% liquid‐free ion‐conductive elastomer remained stable throughout the test without significant change, showing prominent fatigue resistance. These findings suggest that FLICE‐110%, a liquid‐free ion‐conductive elastomer, was poised to achieve significant breakthroughs in the fields of flexible electronics, wearable ionic skins, and human–computer interaction devices, owing to its superior mechanical strength, remarkable durability, high sensitivity, and excellent fatigue resistance.

### 2.5 Multi‐Channel Strain Sensors Based on FLICE‐110% Liquid‐Free Ion‐Conductive Elastomer

**Figure 5:**
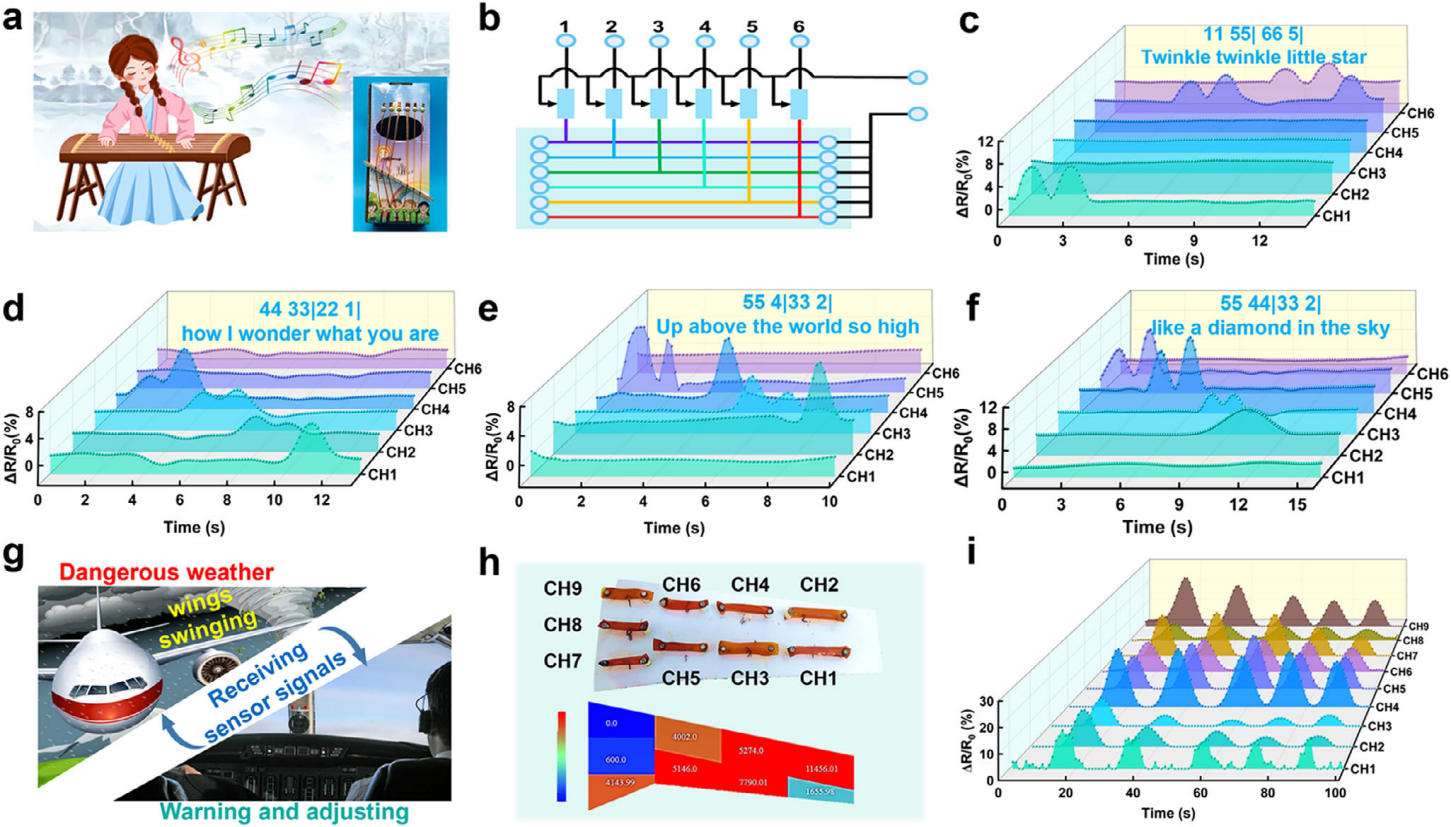
Application of FLICE‐110% liquid‐free ion‐conductive elastomer‐based strain sensors for smart music box and aircraft wing deformation monitoring. a) Schematic diagram of the smart music box based on FLICE‐110% liquid‐free ion‐conductive elastomer sensor. b) Equiv. circuit of the smart music box. c) The electrical signal when playing “Twinkle Twinkle Little Star”. d) The electrical signal when playing “How I wonder what you are”. e) Electrical signals when playing “Up above the World so High”. f) Electrical signals when playing “Like a Diamond in the Sky”. g) Schematic diagram of the user interface for monitoring the deformation of an aircraft wing based on the FLICE‐110% sensor. h) Installation demonstration and virtual simulation of sensing data based on FLICE‐110% liquid‐free ion‐conductive elastomer sensors. i) Relative resistance change monitored by nine strain sensors at different locations of the wing during bending.

Based on the good responsiveness and sensing function of FLICE‐110% liquid‐free ion‐conductive elastomer, we embed FLICE‐110% liquid‐free ion‐conductive elastomer in the Chinese Zither, which can realize the precise sensing of finger touch of players. The schematic diagram of the smart Chinese Zither was shown in **Figure** **5**a, and the device contained six sensors, each corresponding to a different note. The connection principle was shown in Figure 5b, where the six sensors were connected to the LinkZill 01RC to achieve data collection. Figure 5c–f demonstrates the quick response when the Chinese Zither was gently plucked. Finger plucking of different FLICE‐110% liquid‐free ion‐conductive elastomer generated electrical signals to produce different notes. The performance of “Twinkle, twinkle, little star, how I wonder what you are, high above the world, like diamonds in the sky” vividly demonstrated the application of the smart Chinese Zither. Resistive sensors built on FLICE‐110% liquid‐free ion‐conductive elastomer not only exceled in smart music boxes, but also played a vital role in aerospace.

Currently, the aerospace industry plays an important role in people's livelihoods as well as in air transport, and at the same time, higher demands are placed on the safety and lightness of aircraft during flight. Therefore, it was of great practical importance to explore more advanced flexible sensors to accurately locate the position of the wing and monitor deformation to help people react quickly and accurately to potential risks such as gusts of wind and atmospheric turbulence. The high mechanical strength, fast response time, and good tensile properties of FLICE‐110% liquid‐free ion‐conductive elastomers make this strain sensor system promising for application on the wings of low‐flying rescue aircraft, where it can monitor wing deformation in real‐time, thereby effectively sensing changes in airflow and predicting natural weather and emergency events. This will help to better regulate flight direction, make more accurate decisions, and take an important step forward in the rescue business. Tensile strain sensors for low‐altitude rescue aircraft wing monitoring were designed. Sensors were connected to the circuit, and the collected electrical signals were processed by the microcontroller, transmitted to the host computer, and displayed in the user interface, as shown in Figure 5g. Nine sensors were assembled on the wing for the experiment, and the installation results were shown in Figure 5h, and the virtual simulation of each sensor data was performed. A bending test was conducted on the low‐altitude rescue aircraft wing to simulate the effect of airflow bumps on the wing deformation. The strain test results of each sensor under the bending deformation of the wing were shown in Figure 5i and were consistent with the simulation results (Figure 5h). The rate of change of relative resistance showed a periodic variation with the degree of wing bending, indicating that the sensing system had a repeatable response. This will provide a reliable basis for judging the force distribution of each part during flight. However, low‐altitude rescue aircraft will inevitably encounter wet weather while in flight. In order to investigate the performance changes of FLICE‐110% liquid‐free ion‐conductive elastomers in humid environments, a variety of different saturated solutions were prepared to simulate the environments with relative humidity of 11%, 43%, 58%, 75%, and 85%, respectively. To investigate the performance changes of FLICE‐110% liquid‐free ion‐conductive elastomers under different humidity levels (E.g., the saturated LiCl solution has a relative humidity of 11%. The saturated K_2_CO_3_ solution has a relative humidity of 43%. The saturated NaBr solution has a relative humidity of 58%. The saturated NaCl solution has a relative humidity of 75%. The saturated KCl solution has a relative humidity of 85%.), FLICE‐110% liquid‐free ion‐conductive elastomers were left at different humidity levels for 12 h to test the mechanical and electrical conductivity changes. The experimental study showed that the electrical conductivity of FLICE‐110% liquid‐free ion‐conductive elastomer gradually increased with increasing humidity (Figure S31, Supporting Information). This is because humidity changes affect the migration rate of ions within the material, which results in a slight increase in conductivity. We also recognized that if the material is exposed to high humidity for a long period of time, this can lead to a slight swelling and softening, which in turn can lead to a slight decrease in its mechanical properties (Figure S32, Supporting Information). In addition, in order to simulate rainy weather, FLICE‐110% liquid‐free ion‐conductive elastomer was sprayed for different times using a spraying device, and its mass, mechanical properties, and conductivity were tested. The data results show that when washed by rain for 1 h, the mass of FLICE‐110% liquid‐free ion‐conductive elastomers slightly increased at this time because the amount of LiTFSI lost at this time is small, and FLICE‐110% liquid‐free ion‐conductive elastomers are wetted by rainwater. The quality of the FLICE‐110% decreases as the shower time is gradually increased because the loss of LiTFSI increases as the shower time is increased (Figure S33, Supporting Information). The loss of LiTFSI also leads to a gradual decrease in the conductivity of FLICE‐110% liquid‐free ion‐conducting elastomer (Figure S34, Supporting Information), and a gradual decrease in its mechanics due to softening with water (Figure S35, Supporting Information). The FLICE‐110% liquid‐free ion‐conductive elastomers were dried after spraying, and the mass plots of the dried elastomers better show that the mass of the elastomers decreased with the increase of the showering time (Figure S36, Supporting Information), and the electrical conductivity also decreased with the decrease of the showering time (Figure S37, Supporting Information), which is due to the fact that the longer the showering time is, the more the mass of the LiTFSI is lost. The mechanical properties after drying were slightly improved from the original mechanical phase due to the reduction of LiTFSI content (Figure S38, Supporting Information).

The results of these data indicate that prolonged wet conditions and rainfall do lead to a loss of LiTFSI, which affects the change in performance of FLICE‐110% liquid‐free ion‐conductive elastomers, and also has an impact on sensing performance (Figure S39, Supporting Information). We are fully aware of this and are actively taking steps to improve the material's performance. For this purpose, we introduced an encapsulation process to encapsulate the sensors using PDMS and tested their sensing after encapsulation and the sensing signals mounted on the wing of a rescue aircraft. The data showed no significant difference in the sensing signals after encapsulation (Figures S26, S40, Supporting Information), verifying the reliability of the encapsulation for application in different climatic conditions.

In addition, since rescue aircraft may face extreme temperature conditions during flight, such as lower temperatures at higher altitudes and high temperatures due to air friction, we tested the morphological changes and sensing responsiveness of FLICE‐110% at 120 and − 20 °C. In the experiments, we placed FLICE‐110% in high‐temperature and low‐temperature environments and recorded its condition in bending, twisting, and stretching using a thermal infrared display. The results show that the state of FLICE‐110% liquid‐free ion‐conductive elastomer in bending, twisting, and stretching under high and low temperatures is similar to that at room temperature. No obvious changes are observed, and the test results are shown in Figures S41, S42 (Supporting Information). In addition, in order to further investigate its sensing stability at high and low temperatures, we placed FLICE‐110% liquid‐free ion‐conductive elastomer in high‐temperature and low‐temperature environments for 24 h and then conducted sensing tests. The results show that the sensing signal remains stable, indicating that the material still has good sensing ability under high‐temperature and low‐temperature conditions, and the test results are shown in Figures S43, S44 (Supporting Information).

### 2.6 Strain Sensors Based on FLICE‐110% Liquid‐Free Ion‐Conductive Elastomer for Manipulators to Achieve Human‐Computer Interaction

**Figure 6:**
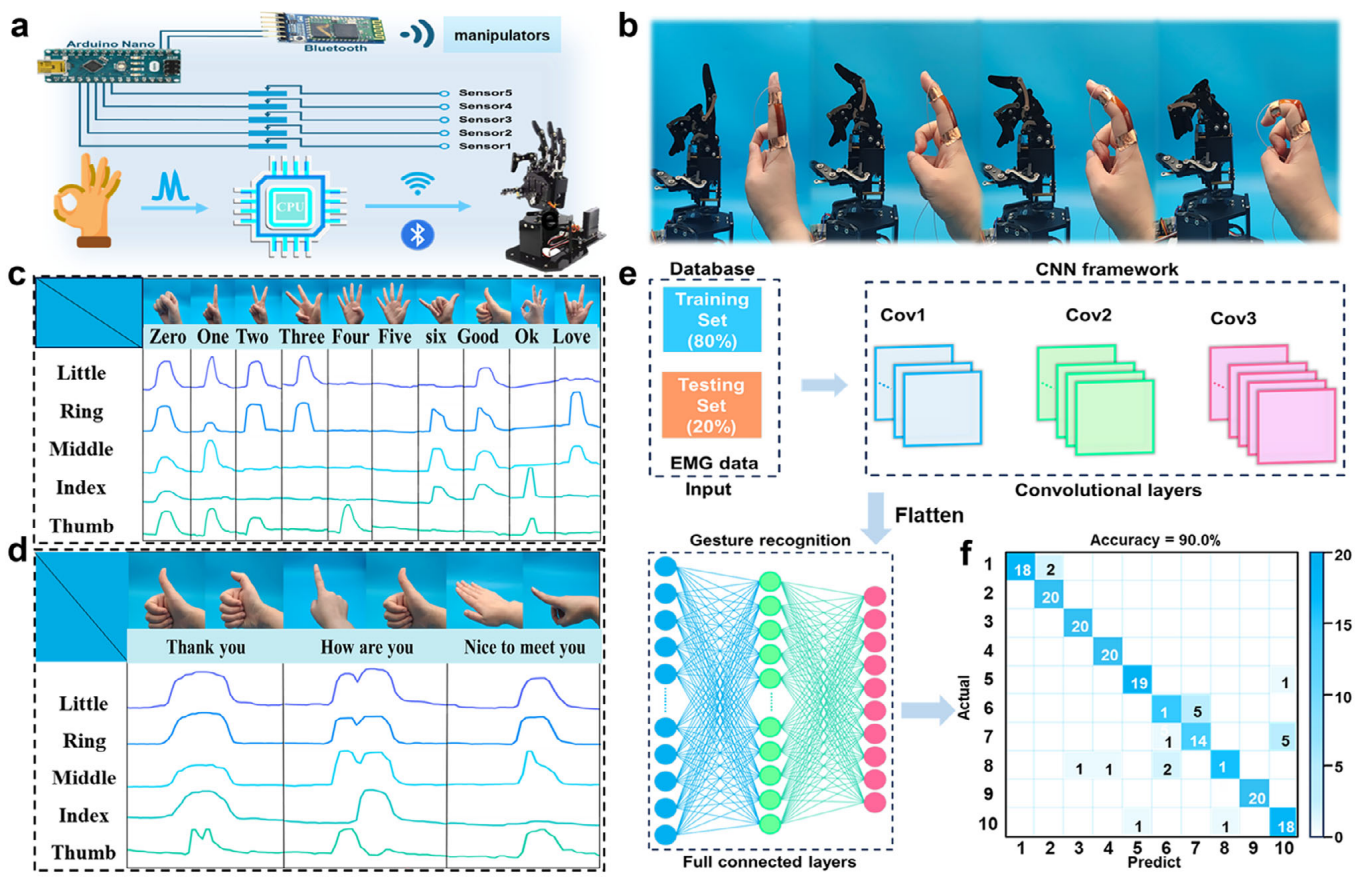
FLICE‐110% liquid‐free ionic conductive elastomer used for remote manipulation of manipulator for human‐computer interaction. a) Circuit diagram of the connection between the microcontroller and Bluetooth used in the system, and schematic diagram of the overall system composition and signal control process. b) Demonstration of the process of FLICE‐110% sensor‐driven manipulator. c) Photographs of ten different gestures and their corresponding electrical signal curves. d) Photographs of three complex sign languages combining multiple gestures and their corresponding signal curves. e) Architecture diagram of the machine learning model. f) Confusion matrix for classification accuracy of ten gesture detections.

Currently, human‐computer interaction plays an important role in modern life, and good design of human‐computer interaction system can greatly improve people's lives. However, limited by traditional sensing materials, the interaction between artificial intelligence and the real world still needs to be improved. FLICE‐110% liquid‐free ion‐conductive elastomer can also play an important role in promoting applications in the field of human‐computer interaction. Compared with traditional rigid materials, FLICE‐110% liquid‐free ion‐conductive elastomer had better fit and the ability to sense small deformations, which provided good prerequisites for human‐computer interaction. Therefore, a wearable prototype device that can remotely drive a manipulator was constructed by using FLICE‐110% liquid‐free ion‐conductive elastomer as a sensing medium in combination with a microcontroller to further verify the sensing reliability of human‐computer interaction devices designed. This system was conducive to the remote control of the manipulator to complete a variety of high‐risk, high‐precision work, such as disarming bombs, rapid ignition, and detonation, to effectively protect personal safety. It also had broad prospects in the field of remote manipulation of surgical systems and intelligent manufacturing, providing new ideas to solve the problems of remote medical care, medical environment, and equipment shortage, as well as improving accuracy and efficiency. The design principle of the system was shown in **Figure** **6**a, which was designed to read signals from five channels that can simultaneously detect the movement changes of five fingers, and also incorporated the Bluetooth module to achieve remote control. The microcontroller read the electrical signals generated by the bending of the fingers and converted them into digital signals. These signals were then wirelessly transmitted to the receiver via the Bluetooth module, where the servo motor drove the robot to bend accordingly, thus enabling human‐computer interaction between the hand and the robot. The details of the microcontroller, Bluetooth, and steering controller were shown in Figures S45–S47 (Supporting Information). By attaching the FLICE‐110% liquid‐free ion‐conductive elastomer to the finger, the manipulator was able to follow the bending movement of the finger to provide a synchronized response (Figure 6b; Movie S1, Supporting Information). The signal curves in Figure 6c effectively responded to the motion status of five fingers for each gesture, verifying the excellent responsiveness of FLICE‐110% liquid‐free ion‐conductive elastomer in monitoring the motion of finger joints. In addition, the FLICE‐110% liquid‐free ion‐conductive elastomer not only generated‌‌ signal changes of a single gesture in real‐time, but also had good recognition and responsiveness to the combination of gestures. Figure 6d shows the sign language meaning of the combined gestures and their corresponding signal curves, breaking the communication barrier between sign language and non‐sign language users. Moreover, in order to improve the recognition accuracy of FLICE‐110% liquid‐free ionic conductive elastomer human‐computer interaction devices, the sensing signals generated by different gestures as the feature information were collected for deep learning, the classification and recognition system was constructed and deep learning training was performed (Figure 6e). The results showed that the validation accuracy of the classification reached a satisfactory 90.0%, and the confusion matrix was shown in Figure 6f. In summary, FLICE‐110% liquid‐free ion‐conductive elastomer can be combined with a human‐machine interface (HMI) to achieve remote manipulation, demonstrating the application of liquid‐free ion‐conductive elastomer strain sensors in HMI.

### 2.7 A Simple and Economical Way to Recover LiTFSI from FLICE‐110% Liquid‐Free Ion‐Conductive Elastomer

LiTFSI, as an important conductive component of elastomers, was widely used to improve the ionic conductivity of materials. The recycling of LiTFSI can significantly reduce the production cost of ionic conductive elastomers, especially in large‐scale production, which helped to improve economic efficiency. At the same time, LiTFSI was difficult to decompose in nature and may cause environmental pollution if not recycled. Therefore, the realization of LiTFSI recycling was of great economic and environmental importance.

In this study, the recovery of LiTFSI was achieved by aqueous dissolution. First, the film‐forming FLICE‐110% liquid‐free ion‐conductive elastomer was cut into small pieces and soaked in distilled water for 24 h. The elastomer solid was filtered off and the water was evaporated by rotary evaporation at 110 °C. Then dichloromethane was added to remove the organic matter, rotary evaporation at 40 °C, and repeated 3 times to obtain pure LiTFSI concentrate. Then isopropyl ether was added to remove inorganic substances, and finally dichloromethane was added to precipitate LiTFSI. After the above treatment procedure, the viscous liquid was recovered and the recovered substance was named R‐LiTFSI. In order to verify whether the viscous liquid was LiTFSI, the recovered viscous liquid was tested in comparison with commercial LiTFSI by FTIR, TGA, and NMR tests.^[^ ^60^, ^61^ ^]^ The FTIR spectra and thermogravimetric curves of the recovered substances were analyzed and the results were very similar to those of commercial LiTFSI (Figure S48a,b, Supporting Information). The ^13^C and ^19^F NMR peaks of the recovered substance were identical to the ^13^C and ^19^F NMR peaks of the commercial LiTFSI without any additional signals, indicating that there were no other C‐containing or F‐containing impurities (Figure S48c,d, Supporting Information). And all of the above results indicated that the recovered substance was LiTFSI, and the recovery efficiency was calculated to be 36%. Among them, we used the recovered LiTFSI to prepare FLICE‐110% liquid‐free ion‐conductive elastomer (named R‐FLICE‐110%) to test its mechanical properties and ion‐conductivity. The results showed that R‐LiTFSI still provided ionic conductivity comparable to that of commercial LiTFSI, and the R‐FLICE‐110% liquid‐free ion‐conductive elastomer still maintained mechanical properties comparable to those of FLICE‐110% liquid‐free ion‐conductive elastomer (Figure S48e,f, Supporting Information). In addition, we investigate the interaction mechanism of LiTFSI with polyurethane matrix. To this end, we conducted a more in‐depth analysis of the relevant experimental data and a systematic exploration using nuclear magnetic resonance (NMR) and molecular simulation, with the aim of revealing the binding mode of LiTFSI in polymer systems in a more comprehensive manner. First, the FTIR analysis results show a significant shift at 1 703 cm⁻¹ in the FLICE‐110% sample compared to the FLICE‐0% sample, which does not contain LiTFSI (Figure 2a). This is due to the coordination between Li⁺ and the carbonyl group (C = O) in the soft segments of the polyurethane. To further verify the existence of this coordination interaction, we conducted ^7^Li NMR tests. The results show that no LiTFSI signal was detected in the FLICE‐0% sample; while in the FLICE‐110% sample, the chemical shift of LiTFSI shifted significantly from − 1.4 ppm (for free LiTFSI) to − 0.02 ppm (Figure S49, Supporting Information), indicating that there is indeed an interaction between the LiTFSI and polyurethane.^[^ ^62^, ^63^ ^]^ After confirming the interaction between Li⁺ and polyurethane, we further investigated the impact of this interaction on the polymer network structure. Through all‐atom MD simulations, we compared the changes in the number of hydrogen bonds in FLICE‐0% and FLICE‐110% (Figure S50, Supporting Information).^[^ ^64^, ^65^ ^]^ The simulation results show that after the introduction of LiTFSI, the number of hydrogen bonds in the system significantly decreases. This change may be attributed to the fact that Li⁺ preferentially coordinates with the oxygen‐containing functional groups on the polyurethane chain, disrupting the stability of the original hydrogen bond network. This phenomenon not only indicates the interaction between the LiTFSI and the polyurethane, but also suggests that it is involved in the polymer network, thereby affecting the mechanical and electrical properties of the material. To further understand the distribution of LiTFSI within the material, we used scanning electron microscopy (SEM) to observe the surface morphology of FLICE‐110% (Figure S51, Supporting Information). The results show that the sample surface is smooth and uniform, with no obvious LiTFSI particles or crystalline precipitation observed. This suggests that LiTFSI has good dispersion within the polymer, with no significant phase separation or local enrichment. This result is consistent with the previous hypothesis of coordination interactions: Li⁺ has been stably embedded in the polymer network in a coordinated state rather than existing in a free state. In addition, in order to further evaluate the effect of LiTFSI doping on the long‐term stability of the material, stability tests were performed on samples that had been previously prepared and stored for 90 days. The mechanical property test results (Figure S52, Supporting Information) show that the material maintained good strength and ductility during storage, with no significant degradation observed. At the same time, the room‐temperature ionic conductivity remained relatively stable over time (Figure S53, Supporting Information), indicating that Li⁺ remains in a stable state within the polymer network, and its conductivity is not significantly affected by prolonged storage.

In summary, various characterization methods and simulation analyses together reveal that LiTFSI forms a stable bond with polyurethane primarily through Li⁺— coordination. This interaction not only improves the dispersion of the LiTFSI in the polymer matrix, but also restructures the network, thereby affecting the hydrogen bond network and the overall properties of the material. Although a large amount of evidence supports this view, we still acknowledge the possible presence of a small amount of free LiTFSI that has not participated in coordination within the system. In future research, we will continue to optimize molecular design and formulation structure, further enhancing the integration of the LiTFSI, and conduct mechanism studies on the long‐term stability of the material at the microscopic level, to promote the practical applications of these materials in flexible electronics and bioelectronic devices. We have successfully recovered LiTFSI from FLICE‐110% liquid‐free ion‐conductive elastomer by the innovative aqueous dissolution method and showed the same electrochemical properties as commercial LiTFSI, which provided a low‐cost strategy for the recovery of LiTFSI and was conducive to the wider development of liquid‐free ion‐conductive elastomers.

## 3 Conclusion

In summary, inspired by the “brick wall” structure of the pearl layer, we have designed a liquid‐free ion‐conductive elastomer, FLICE‐x%, which had a crosslinked network and microphase‐separated structure formed by multiple interacting. This structure enabled liquid‐free ion‐conductive elastomers to combine excellent mechanical properties with high ionic conductivity. FLICE‐110% liquid‐free ion‐conductive elastomer exhibited the best combination of properties, with high mechanical strength (5.46 MPa), high elongation (1 213%), and excellent ionic conductivity (3.29 × 10^−4^ S cm^−1^), as well as good resilience, tear‐resistance, and fatigue resistance, with a fracture energy of 6.25 kJ m^−2^. In addition, the FLICE‐110% liquid‐free ionic conductive elastomer exhibited stable sensing characteristics, with a sensitivity factor of 1.39 at strains above 100%, laying the foundation for the development of multifunctional sensors. FLICE‐110% liquid‐free ion‐conductive elastomer can be applied to multi‐channel strain sensors and HMI technology to effectively capture complex deformation information. FLICE‐110% liquid‐free ion‐conductive elastomer demonstrated excellent flexibility and adaptability, contributing to intelligent control and data analysis. Importantly, this paper also innovatively achieved the recycling of LiTFSI, which was significant for environmental and economic development. In summary, the FLICE‐110% free‐liquid ionic conductive elastomer developed in this study will have broad application prospects, including fields such as flexible electronic devices, wearable devices, biomedicine, soft touch screens, soft robots, and human‐computer interaction.

## Conflict of Interest

The authors declare no conflict of interest.

## Supporting information



Supporting Information

Supporting Information Movie 1

## Data Availability

The data that support the findings of this study are available from the corresponding author upon reasonable request.
