# Supplementary material for: Bioinspired Liquid‐Free Ion‐Conductive Elastomers with Ultrahigh Mechanical Strength and Excellent Ionic Conductivity for Multifunctional Flexible Sensing Applications
Source: Adv Sci (Weinh). 2025 Apr 28;12(27):2503510. doi: 10.1002/advs.202503510 (PMC12279199; doi:10.1002/advs.202503510)
Supplement: Supplementary file 1 — Supporting Information [file ADVS-12-2503510-s002.docx]

**Supporting Information**

**Bioinspired Liquid-Free Ion-Conductive Elastomers with Ultrahigh Mechanical Strength and Excellent Ionic Conductivity for Multifunctional Flexible Sensing Applications**

Zequan Li, Jingjing Tang, Xuwei Wang, Fuqi Wang, Fangyan Ou, Wenyu Pan, Changsheng Wang, Ting Xie, Chuang Ning, Xiwei Xu, Jiamin Liu, Qihua Liang, Wei Gao*, Shuangliang Zhao

Zequan Li, Jingjing Tang, Xuwei Wang, Fuqi Wang, Fangyan Ou, Wenyu Pan, Changsheng Wang, Ting Xie, Chuang Ning, Xiwei Xu, Jiamin Liu, Qihua Liang, Wei Gao

School of Resources, Environment and Materials

Guangxi University

Nanning 530004, China

E-mail: galaxy@gxu.edu.cn (Wei Gao)

Zequan Li, Jingjing Tang, Fuqi Wang, Fangyan Ou, Wenyu Pan, Changsheng Wang, Ting Xie, Chuang Ning, Xiwei Xu, Jiamin Liu, Wei Gao

Guangxi Engineering and Technology Research Center for High-Quality Structural Panels from Biomass Wastes

Guangxi University

Nanning 530004, China

Zequan Li, Jingjing Tang, Wenyu Pan, Ting Xie, Chuang Ning, Xiwei Xu, Jiamin Liu, Shuangliang Zhao

State Key Laboratory of Featured Metal Materials and Life-cycle Safety for Composite Structures

Guangxi University

Nanning 530004, China

Wei Gao, Shuangliang Zhao

Key Laboratory of Disaster Prevention and Structural Safety of Ministry of Education Guangxi University

Nanning 530004, China

Wei Gao, Shuangliang Zhao

Guangxi Key Laboratory of Disaster Prevention and Engineering Safety

Guangxi University

Nanning 530004, China

Shuangliang Zhao

College of Chemistry and Chemical Engineering

Guangxi University

Nanning 530004, China

#### Table of Content

[1. Experimental Section 4](#_Toc194961418)

[1.1. Materials 4](#_Toc194961419)

[1.2. Synthesis of FLICE-x% liquid-free ion-conductive elastomers 4](#_Toc194961420)

[1.3. Fabrication of resistive strain sensors based on FLICE-110% liquid-free ion-conductive elastomer 5](#_Toc194961421)

[1.4. Materials characterization 5](#_Toc194961422)

[1.4.1. General characterization 5](#_Toc194961423)

[1.4.2. Mechanical properties tests 6](#_Toc194961424)

[1.4.3. Ionic conductivity tests 7](#_Toc194961425)

[1.4.4. All-atom molecular dynamics 8](#_Toc194961426)

[1.4.5. Sensitivity testing 9](#_Toc194961427)

[1.4.6. Finite element simulation for tear resistance testing 9](#_Toc194961428)

[1.4.7. Sensor device testing 10](#_Toc194961429)

[1.4.8. Antimicrobial test 10](#_Toc194961430)

[1.4.9. In vitro cytotoxicity assays 10](#_Toc194961431)

[1.4.10 Puncture resistance tests 11](#_Toc194961432)

[1.4.11. Healing efficiency test 11](#_Toc194961433)

[2. Statements 11](#_Toc194961434)

[3. Supplementary Figures 12](#_Toc194961435)

[4. Supplementary Movie 64](#_Toc194961436)

[5. References 64](#_Toc194961437)

# 1. Experimental Section

## 1.1. Materials

Poly(caprolactone)diol (PCL-2OH, *M*_n_ = 2000 g·mol^-1^)， Isophorone diisocyanate (IPDI, 99%), Isophthalic dihydrazide (ID，>95%), 2,5-Dihydroxyterephthalaldehyde (DHTA，98%), and dibutyltin dilaurate (DBTDL, 95%) were purchased from Shanghai Aladdin Biochemical Technology Co., Ltd. N,N-Dimethylacetamide (DMAc, 99.8%), lithium bis(trifluoromethane)sulfonimide (LiTFSI, 98.0%) were purchased from Anhui Senrise Technology Co., Ltd. Hexamethylene diisocyanate trimer (tri-HDI) was purchased from Guangdong Yunxing Biotechnology Co. All reagents were used as the original without further purification.

## 1.2. Synthesis of FLICE-x% liquid-free ion-conductive elastomers

The synthesis of FLICE-x% liquid-free ionic conductive elastomers was carried out by a stepwise synthesis method as shown in Figure S1. PCL-2OH (*M*_n_ = 2000 g mol^-1^, 5.0 g, 2.5 mmol) was first added into a continuously stirred round-bottomed three-necked flask and evacuated at 110 °C for 40 min to fully remove the water and oxygen. Then nitrogen was passed through the system after the evacuation was completed and it was cooled down to 80 °C. IPDI (1.11 g, 5 mmol) was diluted with DMAc (15 ml) and DBTDL (0.02 g) was added as a catalyst, which was mixed well and then added into a round-bottomed triplex flask and reacted at 80 ℃ for 4 h to obtain the polyurethane prepolymer. The temperature was again lowered to 40 °C and isophthalic dihydrazide (0.97 g, 5 mmol) dissolved with DMAc (40 ml) was added and the reaction was stirred for 8 h. Subsequently, the temperature was raised to 60 °C and DHTA (0.42 g, 2.5 mmol) dissolved with DMAc (25 ml) was added and the reaction was carried out for 8 h. Finally, the temperature was raised to 80 ℃, and tri-HDI (0.84 g, 1.67 mmol) was added to react for 4 h. The whole reaction process was carried out under nitrogen protection, and finally, an orange-yellow viscous solution of polyurethane was obtained.

This reaction product was doped with different mass fractions of LiTFSI, (mass fractions of 0%, 70%, 90%, 110%, and 130%, respectively). A corresponding mass of LiTFSI was weighed, and completely dissolved in DMAc and mixed with the above polyurethane at 80 °C for 6 h. The finished solution was poured into a petri dish and heated on a hot plate at 90 °C until the solvent was completely evaporated, resulting in FLICE-x% liquid-free ion-conductive elastomer films.

## 1.3. Fabrication of resistive strain sensors based on FLICE-110% liquid-free ion-conductive elastomer

The FLICE-110% liquid-free ion-conductive elastomer was cut to a size of 20.0 mm × 5.0 mm × 0.6 mm. FLICE-110% liquid-free ion-conductive elastomer was labeled with conductive tape at both ends and two copper wires were led from each end for easy connection and testing. For measurements, resistance strain sensors were fixed to different joint parts of the body and steppers, and copper wires were connected to a Keithley multimeter (Keithley 7510) to monitor real-time resistance changes at different strains.

## 1.4. Materials characterization

### 1.4.1. General characterization

The infrared spectra were obtained by Fourier transform infrared equipped with attenuated total reflectance accessory (ATR-FTIR, IRTracer-100) with a measurement range from 4000 to 650 cm^-1^. X-ray diffraction (XRD) patterns were recorded by an X-ray diffractometer(Rigaku D/MAX 2500V)with a scanning speed of 10° min^-1^ and a scanning range of 2θ = 10 to 50°. The temperature-variable IR spectra were measured by Nicolet iS50 equipped with a thermo controller and the measurement range was from 4000 to 400 cm^-1^. Small-angle X-ray scattering (SAXS) patterns were measured using the Xeuss system (Xenocs 3.0, France). The microphase structure of the samples was probed using an atomic force microscope (AFM) (Dimension Icon). The structure and morphology were characterized by scanning electron microscopy (SEM, Hitachi SU8020) and energy dispersive X-ray spectroscopy (EDS). The thermogravimetric profiles were carried out on a Differential Thermo-Thermogravimetric Analyzer (DTG-60H) with a temperature range of 30-600 °C and a heating rate of 10 min^-1^. Dynamic Mechanical Analysis (DMA) was performed in tensile mode on a Dynamic Mechanical Analyzer (DMA850) with a temperature range of -80 to 100 °C and a heating rate of 5 °C min^-1^.

### 1.4.2. Mechanical properties tests

The mechanical properties of the samples were tested using a BZ2.5/pN1S material mechanics testing system (ZWICK, Germany).

(1)Tensile test: The sample was cut into a dumbbell-shaped strip, the middle strip size was 14.0 mm × 2.0 mm × 0.6 mm, the two ends were fixed to the fixture so that the initial clamping distance was 14 mm, and the tensile speed was set to 50 mm/min. The tensile stress was calculated as follows：

$$\begin{aligned} \sigma=\frac{F}{b*d} \left( S1 \right) \end{aligned}$$

$$\begin{aligned} \varepsilon=\frac{L_{max}-L_{0}}{L_{0}} \left( S2 \right) \end{aligned}$$

Where the *σ* was the tensile stress. The *F* was the maximum tensile force of the specimen at break. The *b* was the width of the effective area of the specimen. The *d* was the thickness of the specimen. The *ε* was the elongation of the specimen at break. The *L*_max_ was the maximum length of the specimen at break, and the L_0_ was the standard length of the sample (14 mm).

In the cyclic tensile test experiments, loading and unloading were performed at a rate of 50 mm min^-1^. The samples were stretched to 500% for five consecutive times without rest in between, and the last lap was heated on a 90 ℃ hot plate for 15 min and loaded/unloaded again. In addition, the samples were loaded/unloaded at 100% strain and sequentially increased to 800%. In cyclic tensile stress-strain diagrams, the hysteresis loop area represents energy dissipation. The calculation formula was as follows：

$$\begin{aligned} Dissipated energy=\left( S_{s}-S_{r} \right)*100\% \#\#\left( S3 \right) \end{aligned}$$

$$\begin{aligned} Hysteresis ratios=\frac{S_{s}-S_{r}}{S_{s}}*100\% \left( S4 \right) \end{aligned}$$

Where *S*_s_ was the area of the boundary between the stretch curve and the X-axis, and *S*_r_ was the area of the boundary between the rebound curve and the X-axis.

In addition, in continuous incremental strain, from 100% strain increment to 800%, the tensile speed remained at 50 mm min^-1^ without any break in between. The elastic recovery (*ER*) values were calculated according to equation：

$$\begin{aligned} ER=\frac{\varepsilon_{max}-\varepsilon_{b}}{\varepsilon_{a}}\times100\% \# \left( S5 \right) \end{aligned}$$

Where *ε*_max_ was the maximum strain in the loading curve for a given cycle, *ε*_b_ was the strain at zero stress in the unloading curve for a given cycle, and *ε*_a_ was the strain at zero stress in the loading curve for a given cycle.^[1]^

(2) Fracture energy test method

The fracture energy was evaluated by tensile testing of unnotched and notched specimens. The dimensions of the specimens were 10.0 mm × 5.0 mm × 0.9 mm, and the notched specimen had a notch length of 1 mm, and both of them were tensile tested at a speed of 3 mm min^-1^. The fracture energy (*G*_c_) can be determined by the following equation：

$$\begin{aligned} G_{c}=\frac{6wc}{\sqrt{\lambda_{c}}} \left( S6 \right) \end{aligned}$$

Where *c* represented the notched length (1 mm), *λ*_c_ represented the elongation at the break of the notched sample, and *w* represented the strain energy, calculated by integrating the stress-strain curve of the unnotched sample to 𝜀_c_ ( *ε*_c_=*λ*_c_-1).^[2-3]^

### 1.4.3. Ionic conductivity tests

The electrochemical impedance spectra (EIS) of FLICE-x% were measured by an electrochemical workstation (CHI660E). Firstly, the sample to be tested was cut into a circular specimen with a diameter of 16 mm and an area of 64 mm^2^, and its thickness was recorded. Measurements can be initiated by placing the sample to be measured between two stainless steel electrodes and connecting it to the electrochemical workstation (CHI660E). Measurements were made at room temperature, at frequencies from 0.001 Hz to 1.0 MHz, and with an amplitude of 1.0 V. The formula for calculating the ionic conductivity of FLICE-x% liquid-free ionic conductive elastomers was as follows：

$$\begin{aligned} \sigma=\frac{L}{R*S} \left( S7 \right) \end{aligned}$$

Where the *L* was the thickness of the sample to be measured. The *R* was the volume resistance of the sample to be measured (derived from EIS). The *S* was the contact area between the sample and the steel electrode.

### 1.4.4. All-atom molecular dynamics

Using all-atom molecular dynamics (MD) simulations to explore the adsorption effect of FLICE-110% liquid-free ion-conductive elastomer on Li^+^. In the MD simulation, three simulation systems were created. Initial configuration of a simulated system built using Materials Studio's Amorphous Cell module by randomly distributing FLICE-110% liquid-free ion-conductive elastomer preparation materials in a cube, where the initial density of the polymer was set to 0.1 g cm^-3^. Prior to MD simulations, all feedstocks were optimized to optimize the energy and force tolerances of the two systems to 0.001 Kcal mol^-1^ and 0.5 Kcal (mol^-1^ ·Å^-1^), respectively. In order to eliminate potential surface effects, periodic boundary conditions (PBC) were applied in three orthogonal directions using the COMPASS (Condensed Phase Optimized Molecular Potential for Atomistic Simulation Studies) III force field, while partial atomic charges were assigned using the forcefield assignment method. For non-bonding interactions in the system, 9-6 Lennard-Jones used a Jones potential with a cutoff distance of 12.5 Å to describe the van der Waals (vdW) forces between the atoms, while the standard Coulomb potential was used to model the electrostatic interactions of the atoms as evaluated by the Ewad algorithm. This was followed by a polyurethane reaction simulation using the official Dassault script, with the final cross-linking degree set to 95%. After the calculations were completed, all models were optimized to optimize the energy and force tolerances of the two systems to 0.001 Kcal mol^-1^ and 0.5 Kcal (mol^-1^ ·Å^-1^), respectively. The optimized structure was annealed within 5 ns by an annealing cycle involving a linear heating process from 303.15 to 553.15 K and a linear cooling process from 553.15 to 303.15 K. The annealed structure was then annealed by an annealing cycle involving a linear heating process from 303.15 to 553.15 K and a linear cooling process from 553.15 to 303.15 K. Subsequently, an additional 5 ns of MD simulations were performed at temperatures of 303.15 or 353.15 K and an enclosing pressure of 1 e^-4^ GPa in the NPT set to further relax the system. Finally, production simulations were performed to calculate the system's adsorption of Li^+^. The motion of the atoms in the system follows classical Newtonian motion, where a velocity-Verlet algorithm with a time step of 1.0 fs was used to integrate the classical Newtonian equations. All MD simulations were implemented using the Forcite module of Materials Studio.

### 1.4.5. Sensitivity testing

Gauge factor (GF) is an important metric for evaluating the sensitivity of a sensor. This was calculated by first performing a tensile test with a 10.0 mm × 3.0 mm × 0.5 mm resistive transducer in a stepper machine, with the ends of the transducer connected to a Keithley multimeter (Keithley 7510) to monitor the real-time resistance change at different strains. The *GF* formula was as follows：

$$\begin{aligned} GF=\frac{\left[ \frac{\left( R-R_{0} \right)}{R_{0}} \right]}{\varepsilon} \left( S8 \right) \end{aligned}$$

where the *R*_0_ was the initial resistance of the elastomer before stretching. The *R* was the real-time resistance of the elastomer after stretching, and the *ε* was the applied strain.

### 1.4.6. Finite element simulation for tear resistance testing

Mechanical simulations of the tear resistance model were created using ANSYS Workbench software. Ansys workbench 2022 was used to build the finite element and geometric model of FLICE-110% liquid-free ion-conductive elastomer. The model size was 10.0 mm × 5.0 mm × 0.6 mm, the crack length was c = 1 mm, and the left end boundary was fixed. The displacement load was applied to the right end boundary, and the contact cohesion model was defined using the insert command. The mesh was divided by several calculations in order to select the appropriate mesh size, and 0.6 mm was chosen for this simulation. The mesh was refined at the top of the crack with a ball radius of 2.0 mm and a cell size of 0.1 mm. The model dimensions corresponded to the dimensions of the specimens prepared in the laboratory.

### 1.4.7. Sensor device testing

Relative resistance changes of wearable strain sensors based on FLICE-110% liquid-free ion-conductive elastomer by Keithley multimeter (Keithley 7510). All experiments conducted in this work involving human volunteers were in full compliance with all local laws and institutional ethical guidelines. Wearable sensors were mounted on the skin of human subjects after receiving their approval to monitor a range of body activities and physiological electrical signals. Importantly, these procedures did not adversely affect participants physically or psychologically during or after the experiment. The relative resistance change (*ΔR/R*_0_) was calculated according to the following equation：

$$\begin{aligned} \frac{\Delta R}{R_{0}}=\frac{R-R_{0}}{R_{0}}\times100\% \left( S9 \right) \end{aligned}$$

Where *R*_0_ and *R* were the initial and real-time resistance of the FLICE-110% based wearable sensor, and *ΔR* was the difference between R and R_0_.

### 1.4.8. Antimicrobial test

Resuscitation of single colonies of Escherichia coli and Staphylococcus aureus on agar medium. The bacteria obtained were diluted to 10^6^ CFU/mL with 0.3 ml of phosphate-buffered saline (PBS) (pH 6.8), and 100 μL of the diluted bacterial solution was evenly applied to the surface of the medium and placed flat using an applicator stick and pipette gun. Next, a film of the sample to be tested, cut to a diameter of 8 mm, was placed in the middle of the medium, and the size of the ring of inhibition on the medium was observed after incubation at 37 °C for 24 h.

### 1.4.9. In vitro cytotoxicity assays

Cytotoxicity testing was conducted using mouse fibroblasts (L929) cells, with the CCK-8 assay method employed. The test method was as follows, FLICE-110% liquid-free ionic conductive elastomer samples were sterilized by irradiation under UV light for 0.5 h and immersed in Dulbecco's modified eagle's medium for 48 h under ambient conditions of 37 ℃ in order to obtain the extracts. L929 cells were cultured in a complete growth medium consisting of DMEM, 10% fetal bovine serum, and 1% penicillin/streptomycin solution in a humidified environment and 5% CO_2_. The cell suspension (100 μL) was then seeded into 96-well plates with 1 × 10^4^ cells per well. Once the cells were attached to the plate, the medium was removed and refilled with 100 μL of FLICE-110% liquid-free ionic conductive elastomer sample extract. Cells incubated in a complete medium without FLICE-110% liquid-free ionic conductive elastomer extract served as controls. Cell viability was assessed with CCK-8 reagent at 450 nm according to the manufacturer's instructions at 3 h, 6 h, 12 h, 24 h, 36 h, and 48 h, respectively.

### 1.4.10 Puncture resistance tests

The puncture tests were performed with a sample−holding apparatus and needle on an Exceed E42 electronic universal testing machine at a 50 mm min^−1^ crosshead speed. The FLICE-110% liquid-free ion-conductive elastomer thickness was about 677 μm.

### 1.4.11. Healing efficiency test

The samples were repaired by cutting a slit in the sample with a scalpel, applying a drop of DMAC to the sample, and placing it on a hot plate at 130 °C.After healing, the self-healing samples were subjected to tensile test and conductivity test.

The healing efficiency was calculated by the following formula:

$$\begin{aligned} H=\frac{\sigma_{h}}{\sigma_{0}}\times100\% \left( S10 \right) \end{aligned}$$

where *σ*_0_ is the tensile stress of the original sample, and *σ*_h_ is the tensile stress of the self–healed sample.

# 2. Statements

All human study participants provided informed written consent for the experiments.

# 3. Supplementary Figures


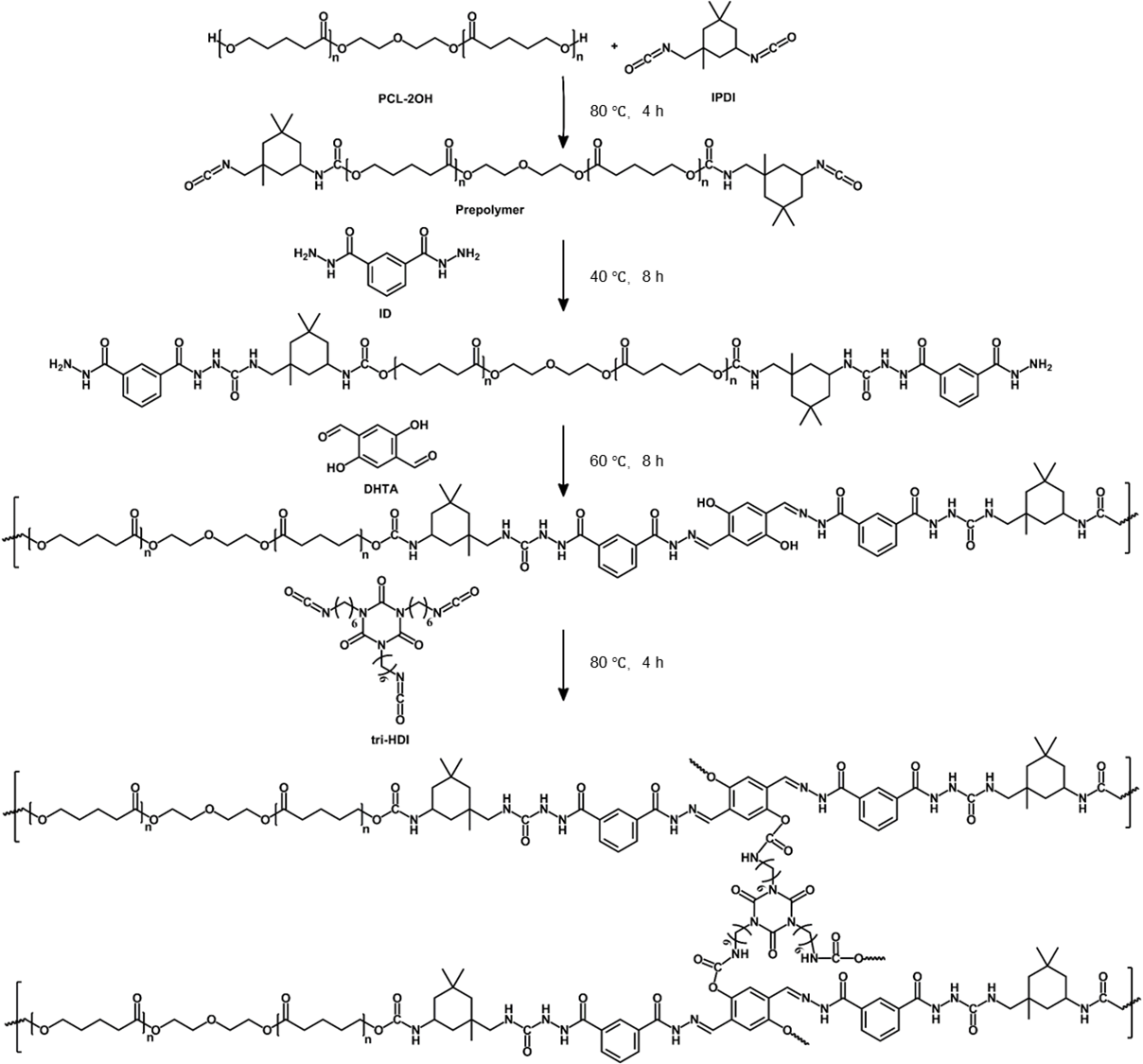


Figure S1. Synthesis route for FLICE-x% polyurethane elastomers.


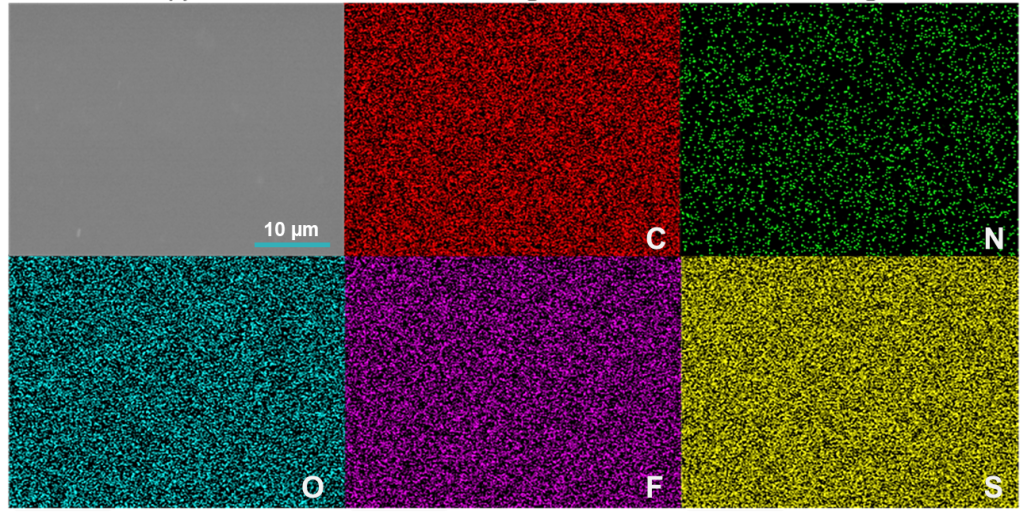


Figure S2. Scanning electron microscopy (SEM) and energy dispersive X-ray spectroscopy (EDS) patterns of FLICE-110% liquid-free ion-conductive elastomer.


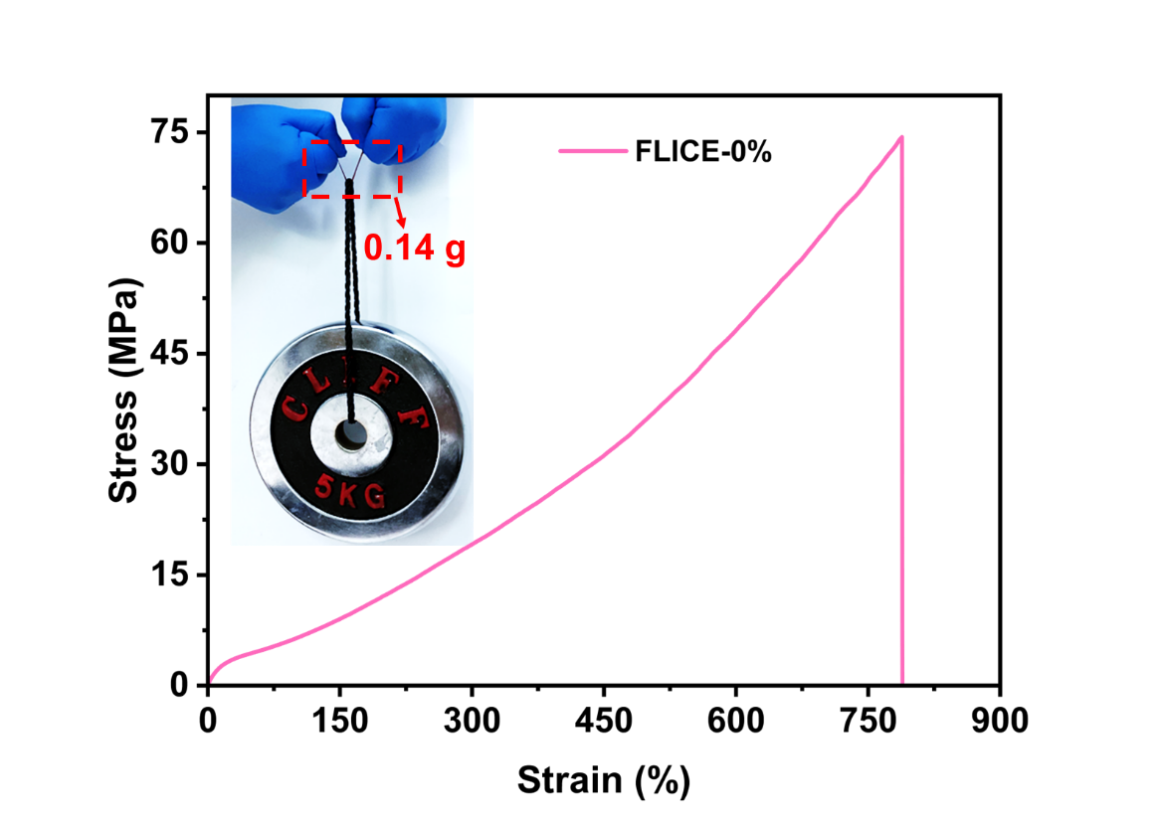


Figure S3. Typical stress-strain curve for FLICE-0% polyurethane elastomer. The inset showed a photograph of a sample of FLICE-0% polyurethane elastomer (0.14 g) that can pull up a 5 kg weight.


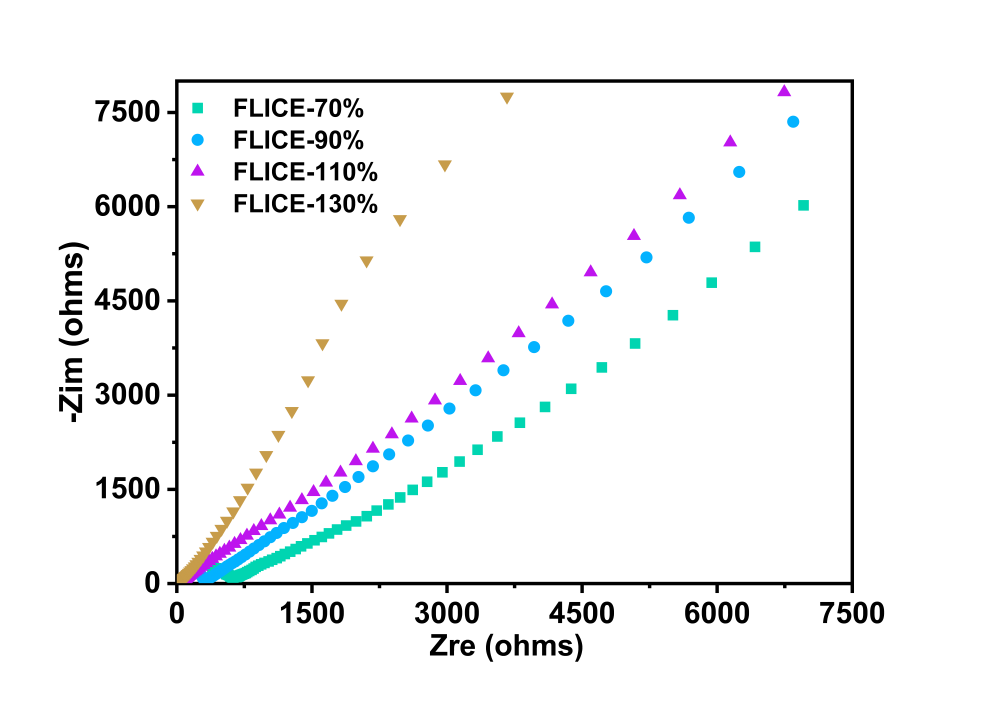


Figure S4. Electrochemical impedance spectra of FLICE-x% polyurethane elastomers at room temperature.


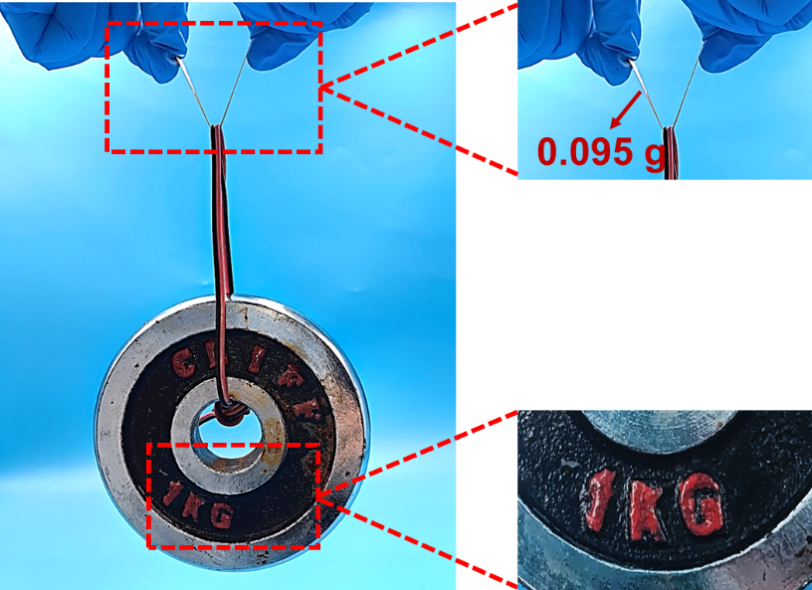


Figure S5. The photos showed that a sample of FLICE-110% liquid-free ion-conductive elastomer (0.095 g) can pull up a 1 kg weight.


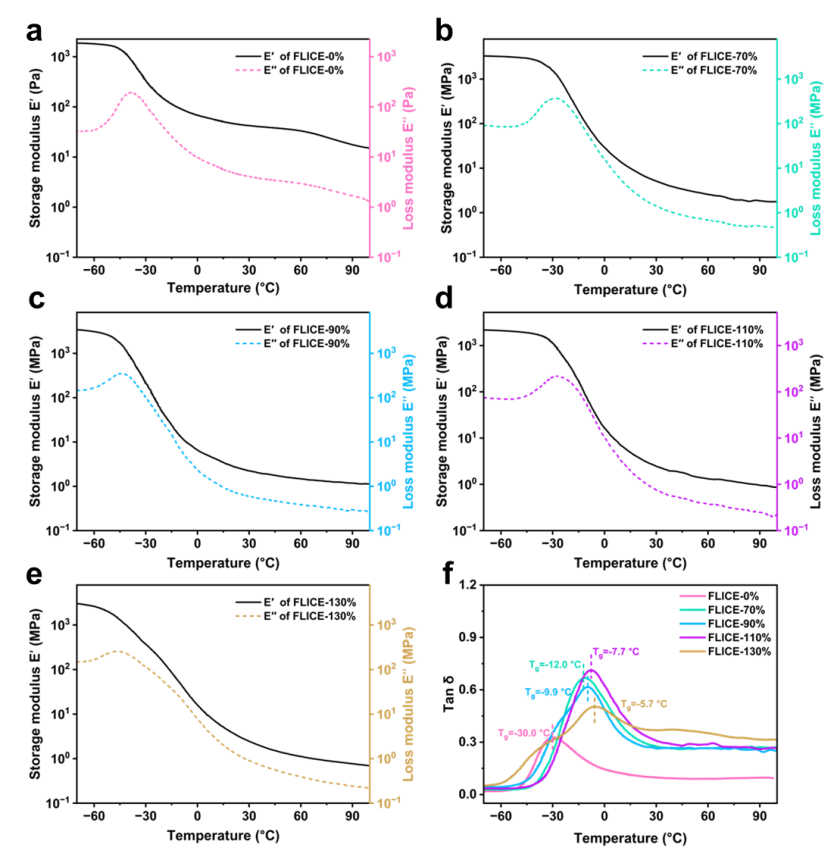


Figure S6. DMA test results for FLICE-x% polyurethane elastomers. (a) Storage modulus and loss modulus of FLICE-0% polyurethane elastomer. (b) Storage modulus and loss modulus of FLICE-70% liquid-free ion-conductive elastomer. (c) Storage modulus and loss modulus of FLICE-90% liquid-free ion-conductive elastomer. (d) Storage modulus and loss modulus of FLICE-110% liquid-free ion-conductive elastomer. (e) Storage modulus and loss modulus of FLICE-130% liquid-free ion-conductive elastomer. (f) The tan δ of FLICE-x% polyurethane elastomers.


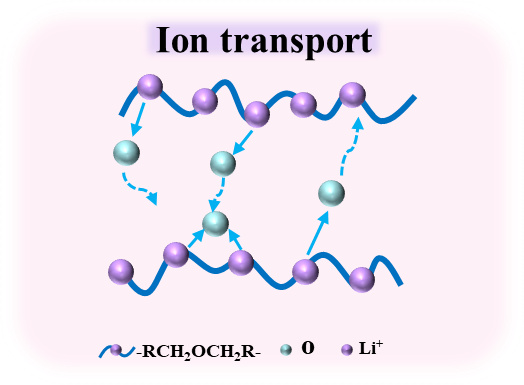


Figure S7. Ion transport mechanism of FLICE-110% liquid-free ion-conductive elastomer.


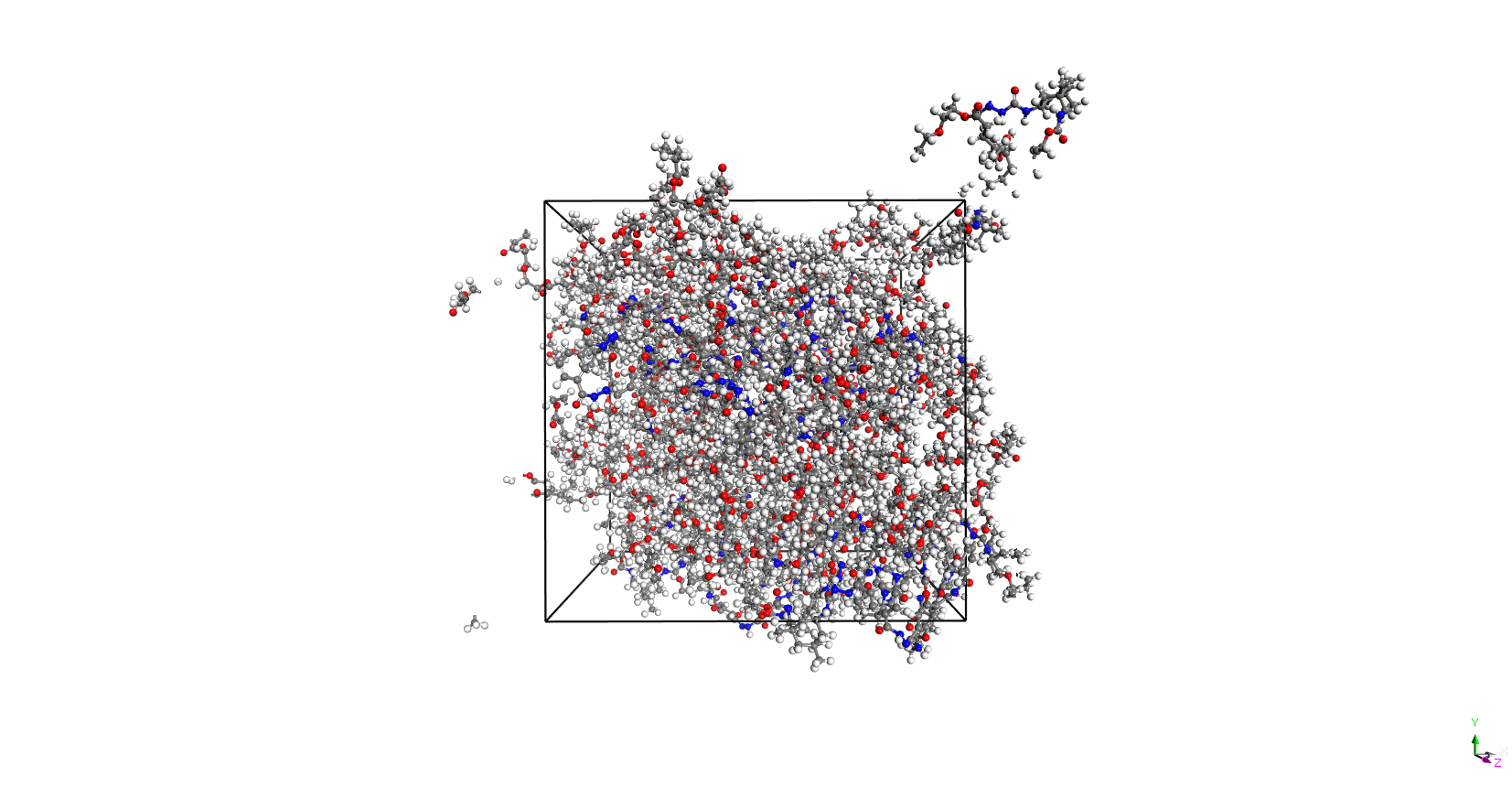


Figure S8. Snapshots of MD simulations of FLICE-110% liquid-free ion-conductive elastomer structures.


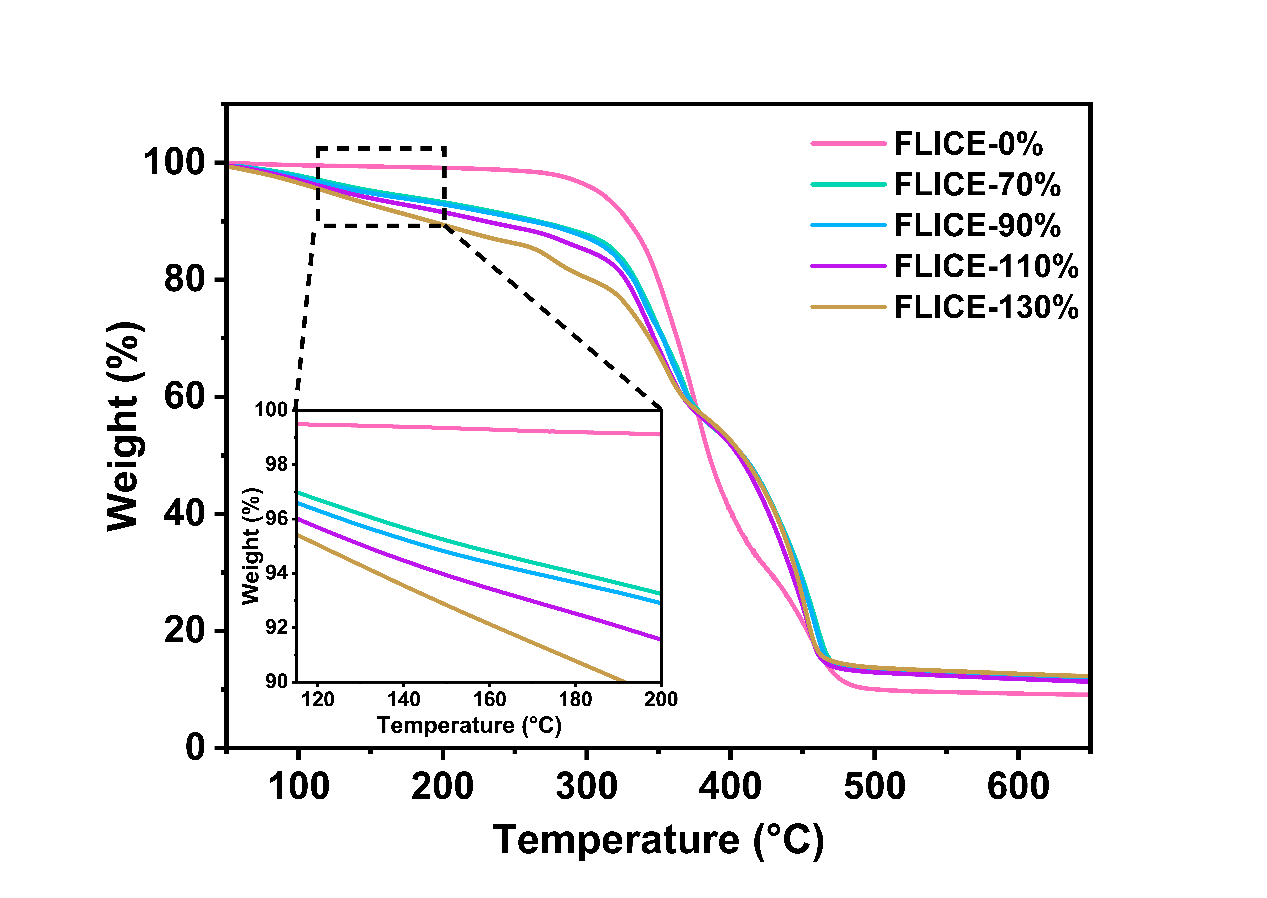


Figure S9. Thermogravimetric analysis of FLICE-x% polyurethane elastomers.


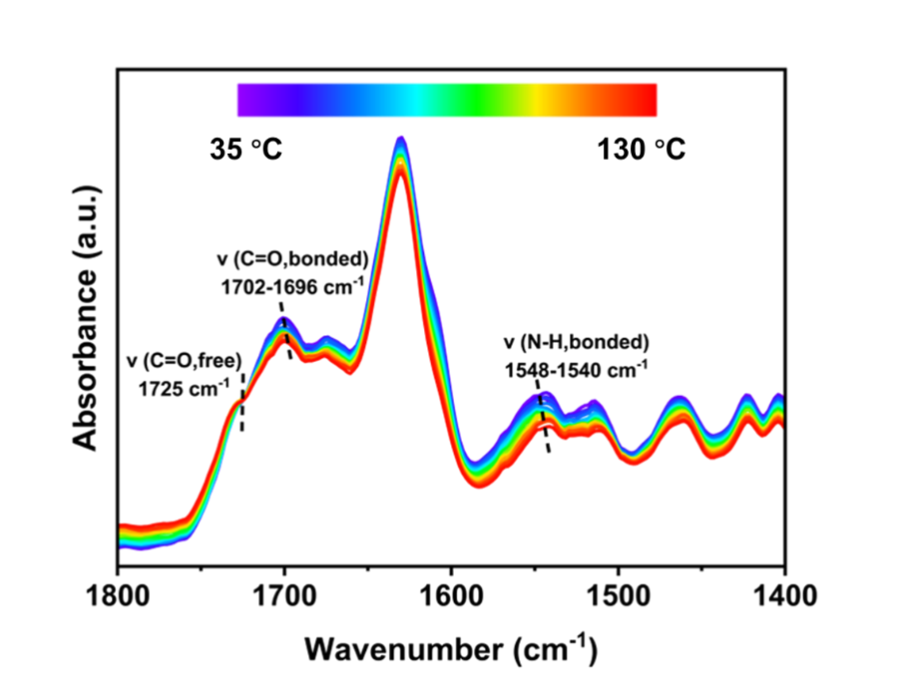


Figure S10. The temperature-variable IR spectra of FLICE-110% liquid-free ion-conductive elastomer.


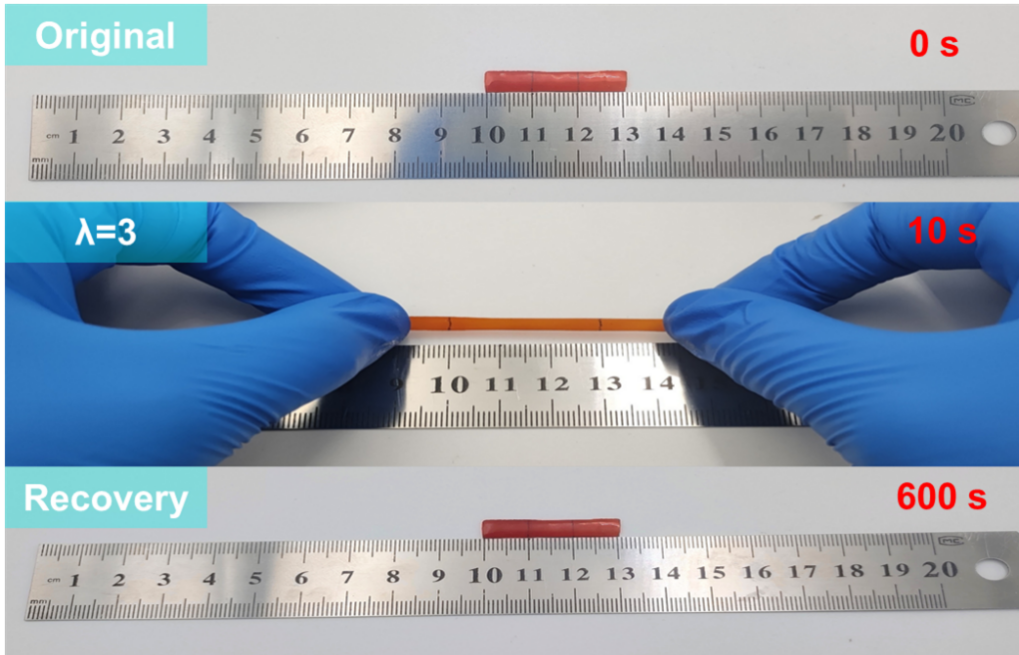


Figure S11. Photos of FLICE-110% liquid-free ion-conductive elastomer sample from 0% stretch to 300% strain. When stretched to 3 times its original length, hold it for 10 s. After releasing the stress and resting for 600 s, it basically returned to its original size.


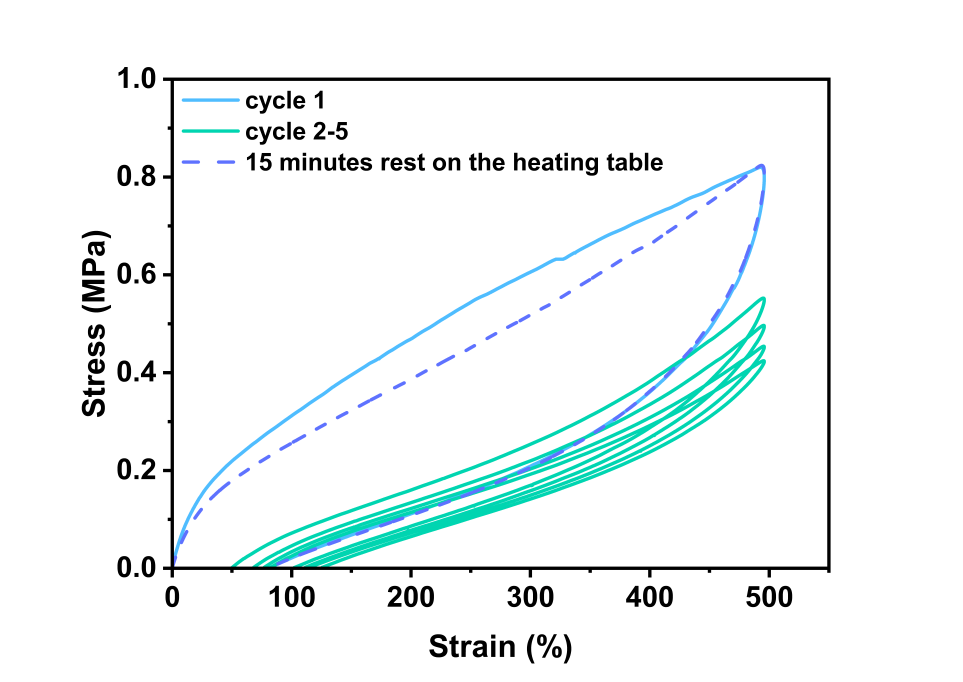


Figure S12. Continuous cyclic tensile stress-strain curve of FLICE-110% liquid-free ion-conductive elastomer from 0% stretch to 500%. The cycling curve almost overlapped with the first cycling curve after 15 min of standing on a heated bench at 90 °C.


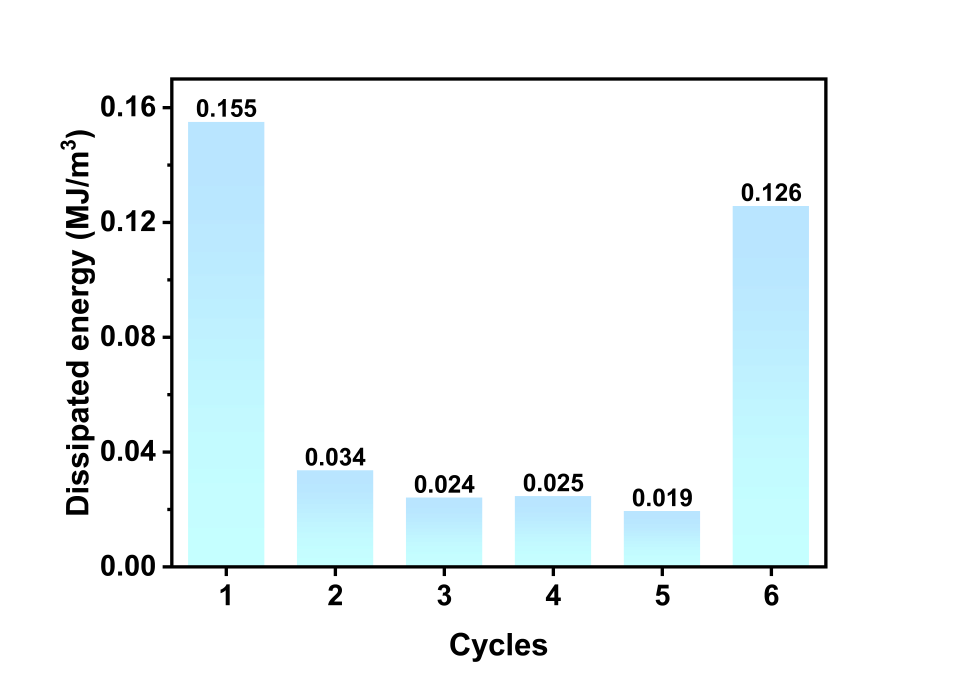


Figure S13. The dissipated energy of FLICE-110% liquid-free ion-conductive elastomer of each repeating cyclic tensile cycle.


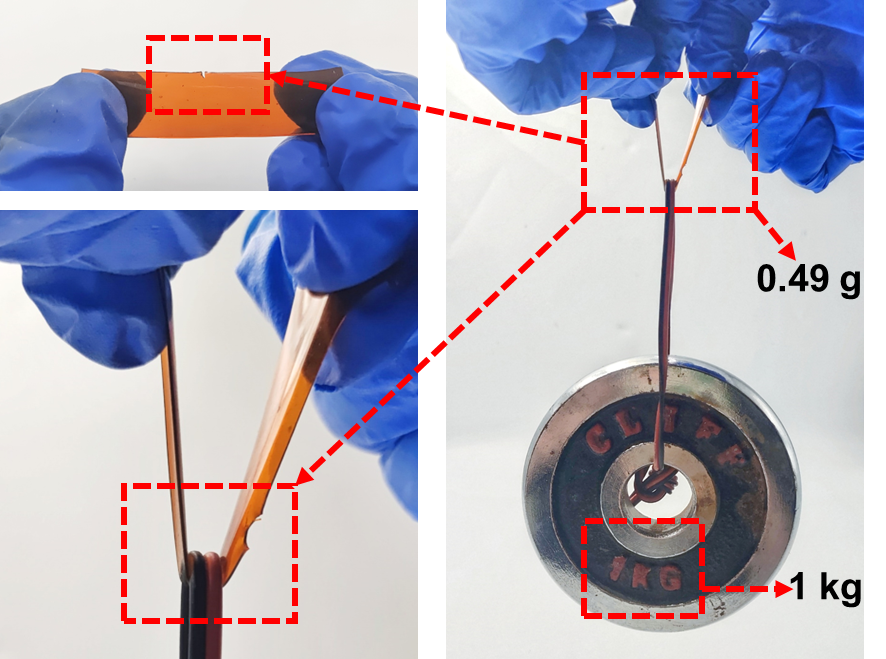


Figure S14. The photos showed that FLICE-110% liquid-free ion-conductive elastomer (0.49 g) with a 1 mm notch was able to pull up a 1 kg weight.


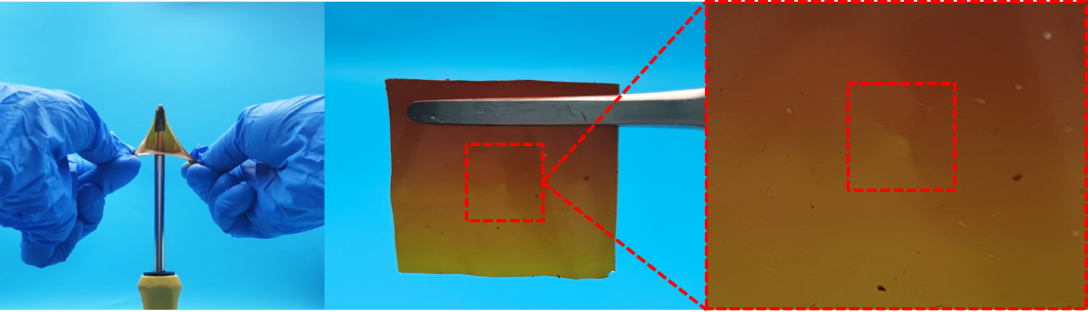


Figure S15. The photos showed the performance of FLICE-110% liquid-free ion-conductive elastomer film punctured by a sharp needle.

**
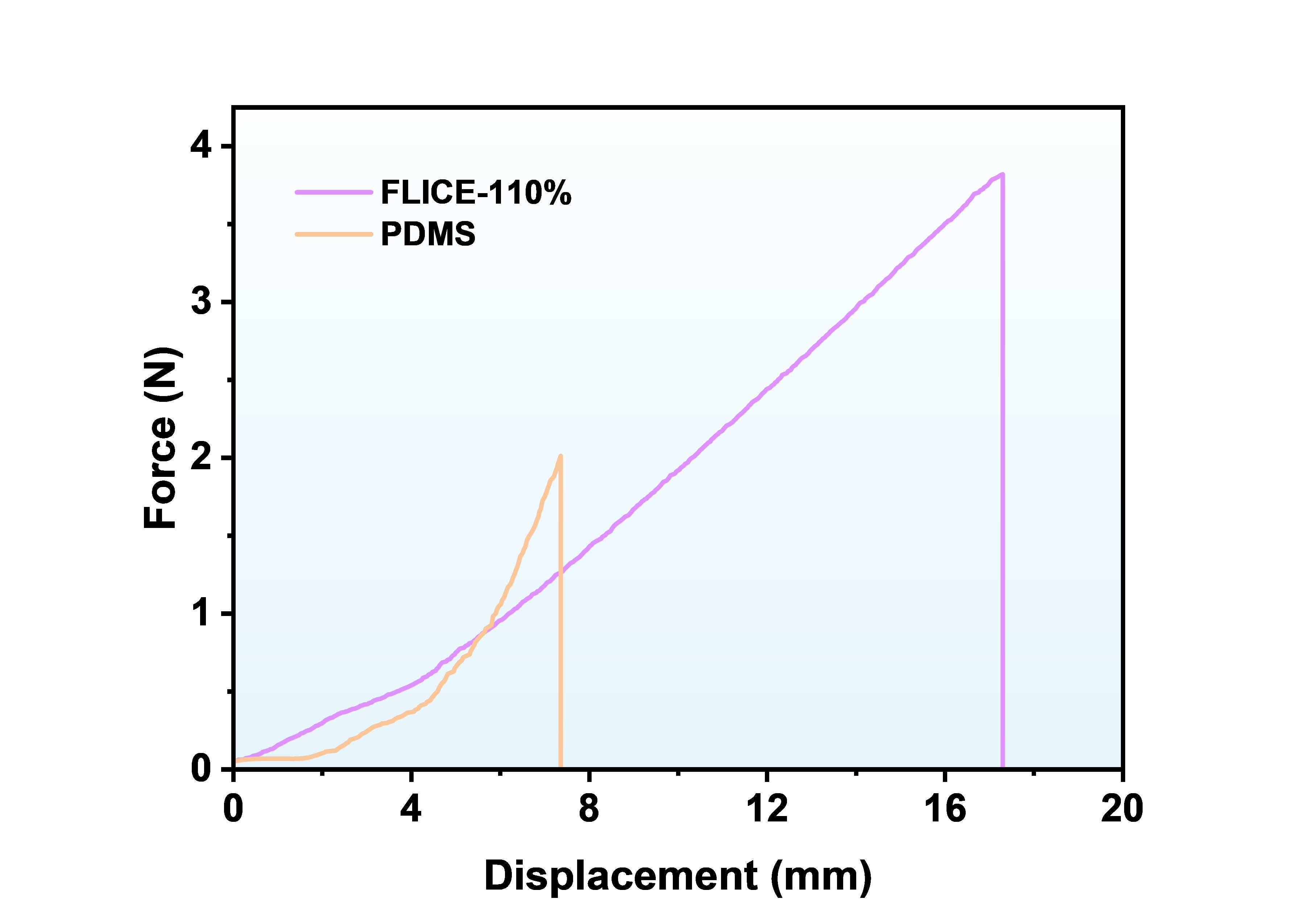
**

Figure S16. Force-displacement diagram of FLICE-110% liquid-free ion-conductive elastomer and PDMS.


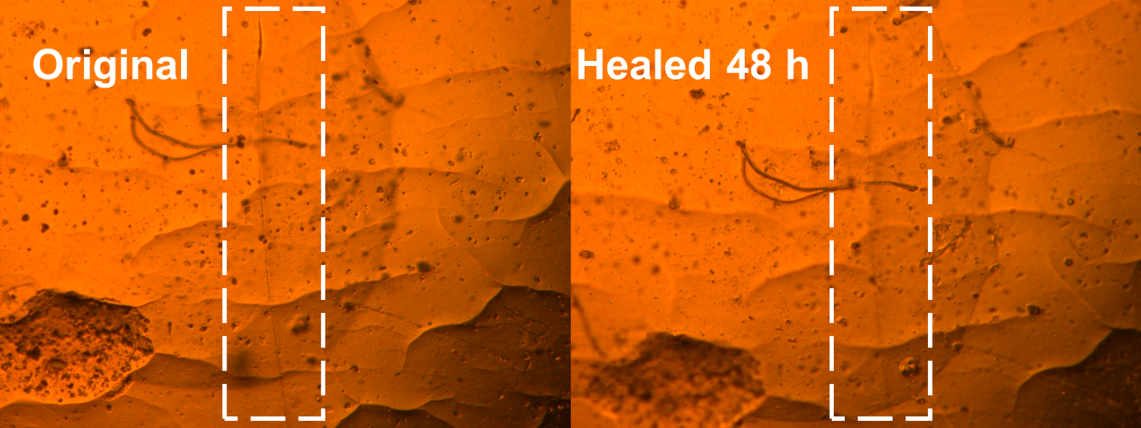


Figure S17. Optical microscope photographs of FLICE-110% liquid-free ion-conductive elastomer before and after scratch healing. Healing condition: heating at 130 °C for 48 h.

**
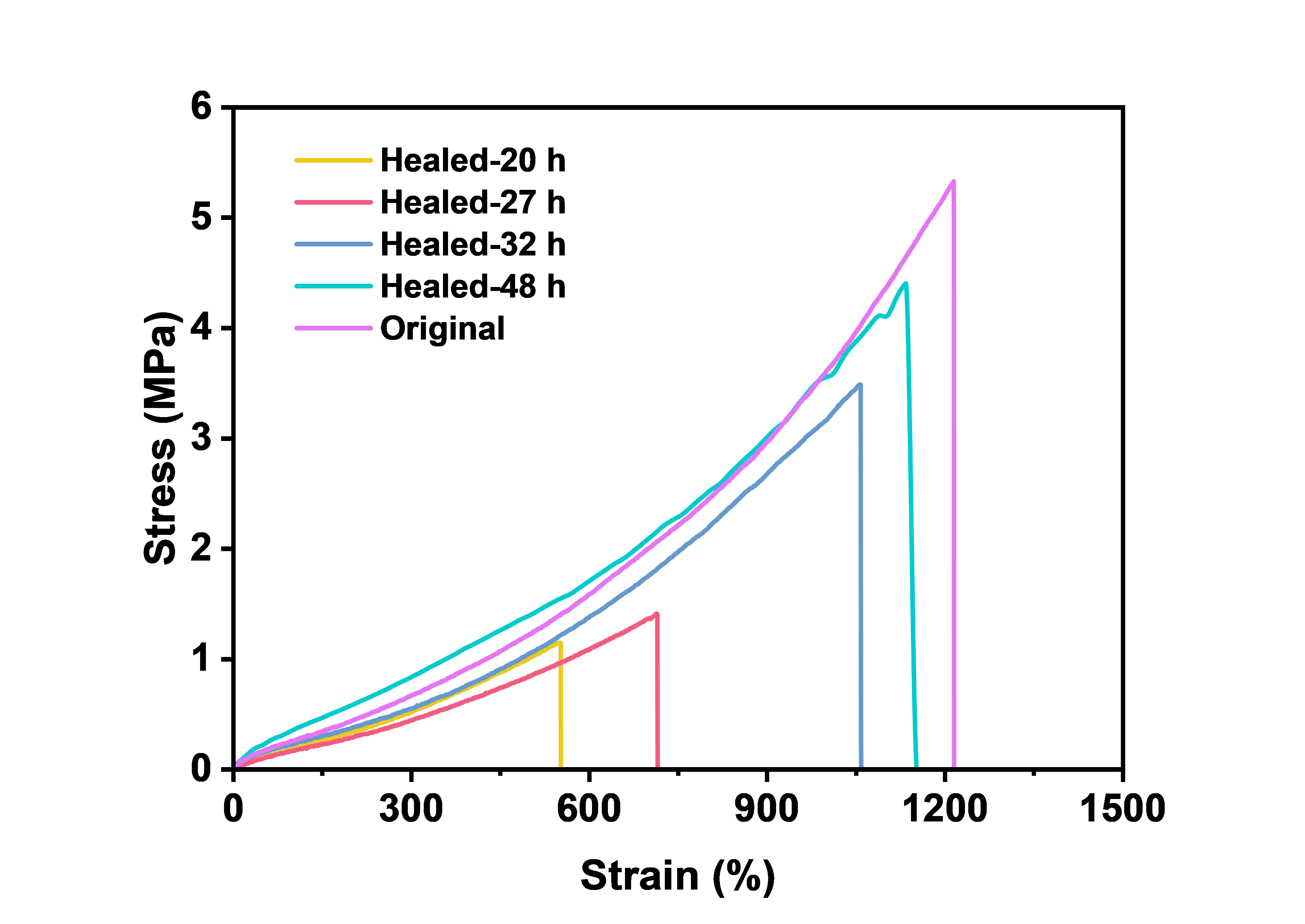
**

Figure S18. Mechanical properties of FLICE-110% liquid-free ion-conductive elastomers self-healing for different times.


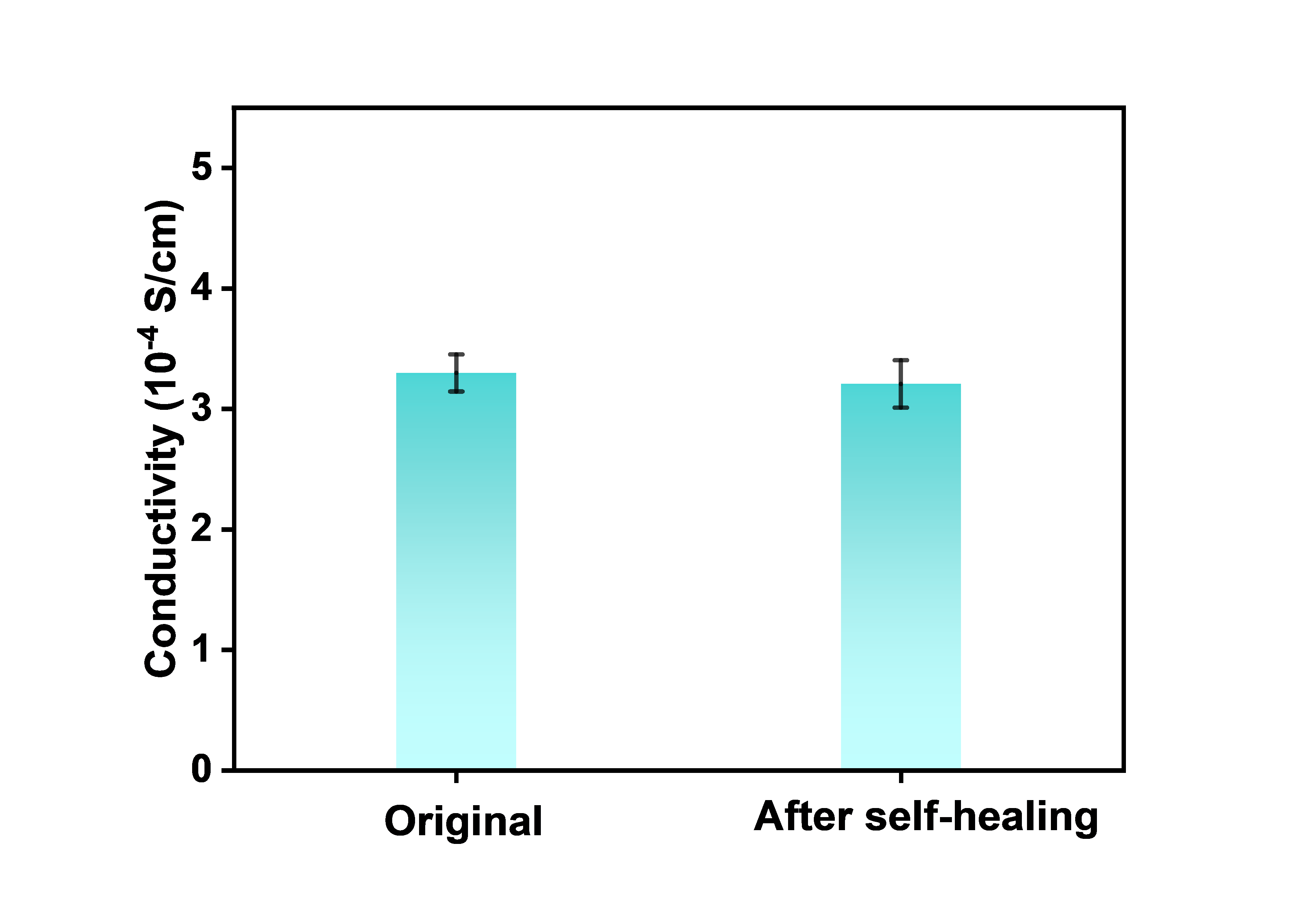


Figure S19. Plot of conductivity comparison before and after self-healing of FLICE-110% liquid-free ionic conductive elastomer.


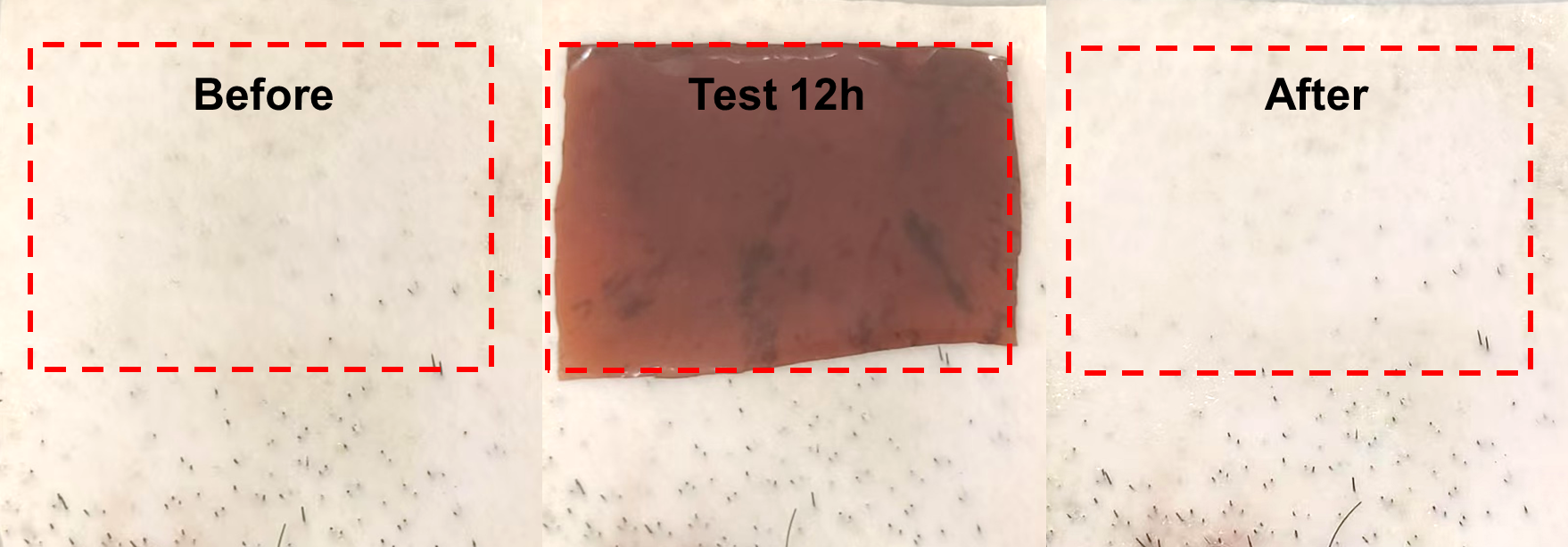


Figure S20. Demonstration of simulated sweating environment on pig skin to observe the skin condition of pig skin.


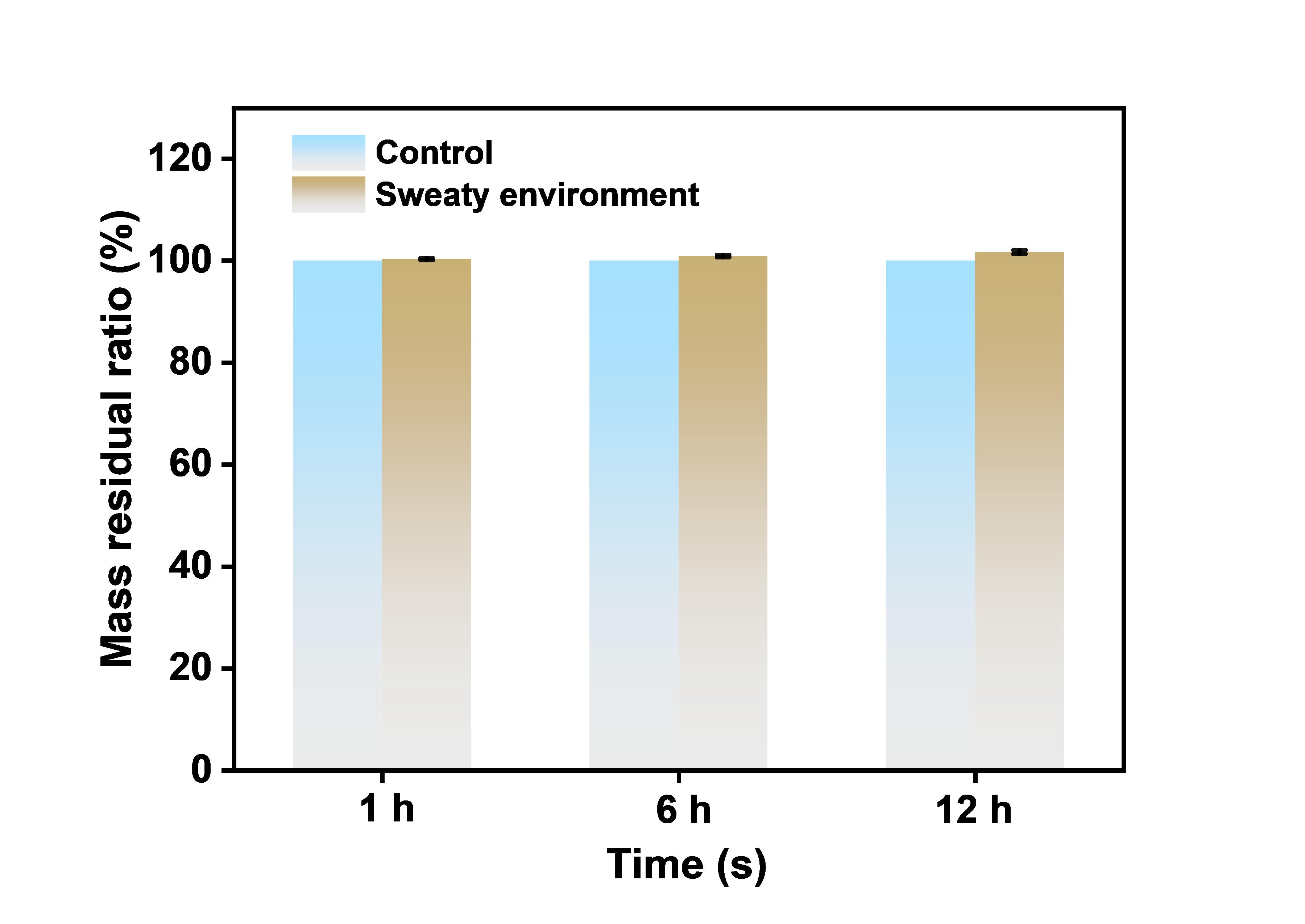


Figure S21. Mass change of FLICE-110% liquid-free ionic conductive elastomer in sweat environment.


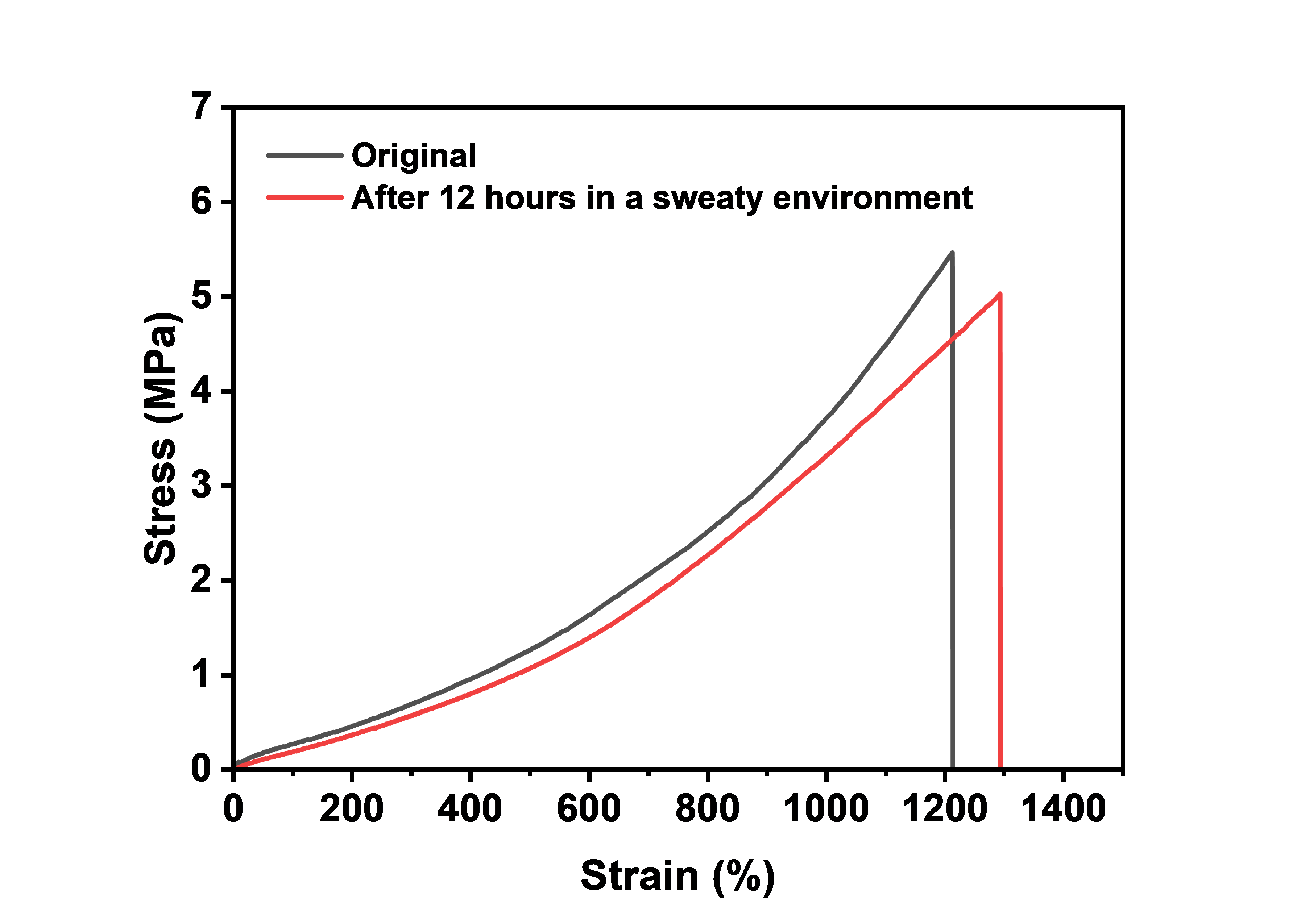


Figure S22. Mechanical properties of FLICE-110% liquid-free ion-conductive elastomer after 12 h in a sweaty environment compared to the original mechanical properties.


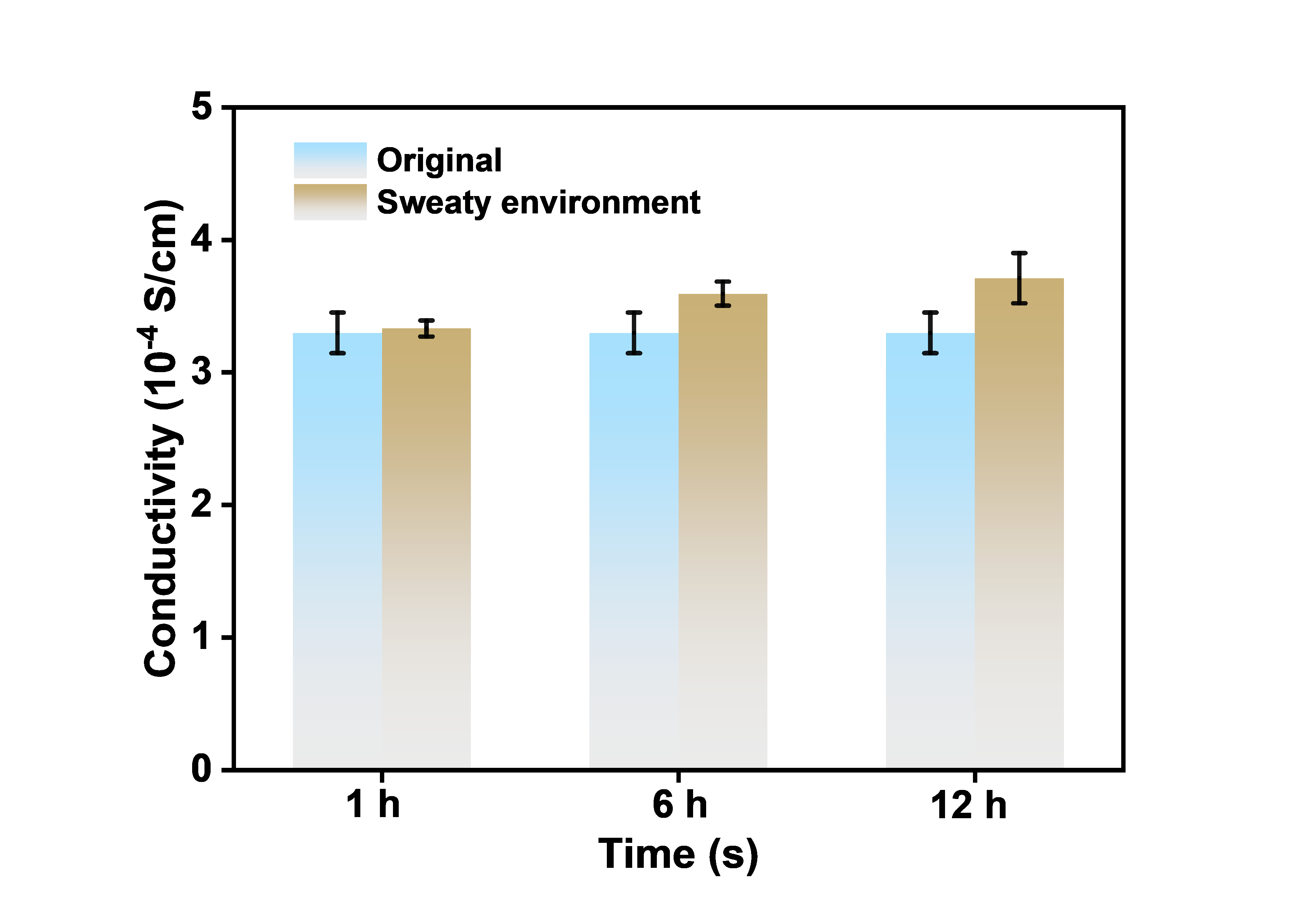


Figure S23. FLICE-110% Liquid-free ionic conductive elastomer conductivity in a sweat environment compared to the original conductivity.


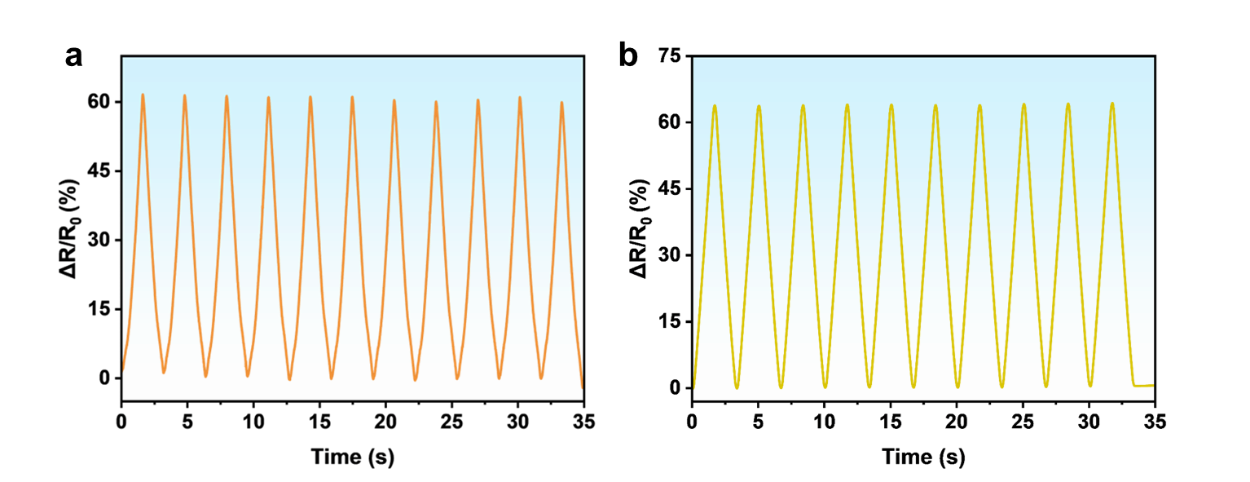


Figure S24. (a) Sensing performance of FLICE-110% liquid-free ion-conductive elastomers in dry environments. (b) Sensing performance of FLICE-110% liquid-free ion-conductive elastomers after 12 h in a sweat environment.


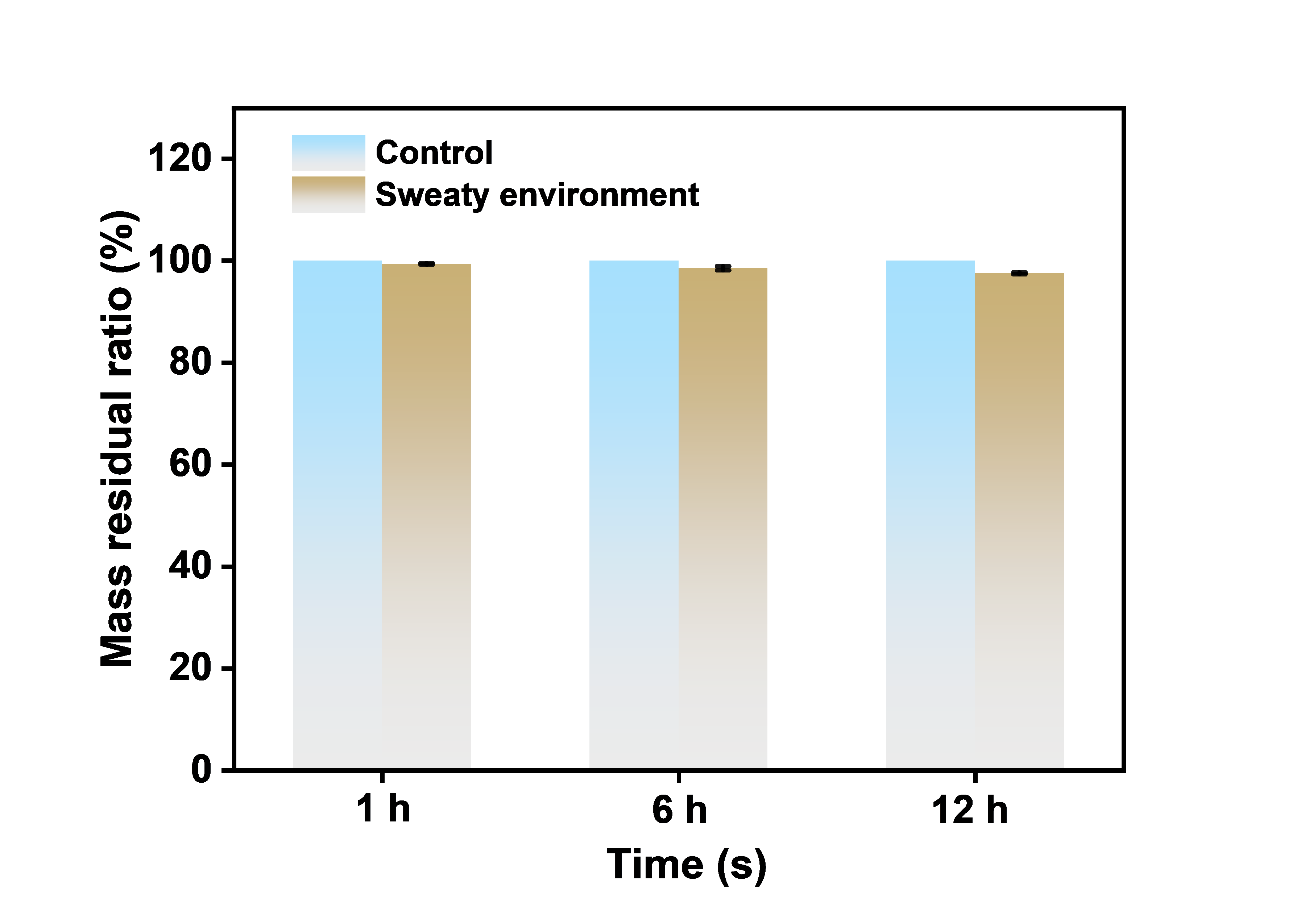


Figure S25. FLICE-110% Changes in the quality of liquid-free ion-conductive elastomers after removal from the sweat environment and drying.


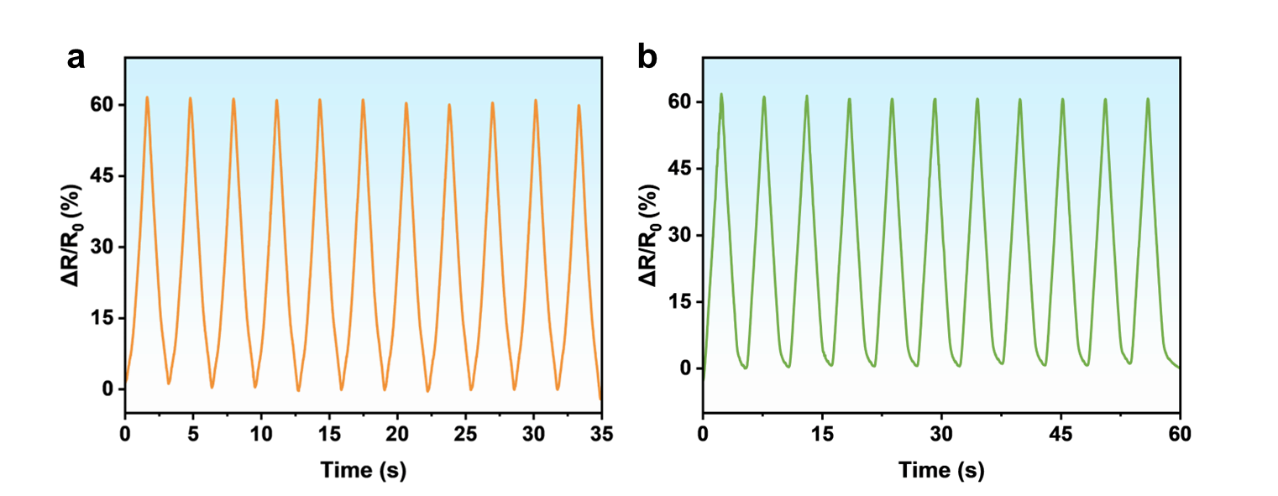


Figure S26. (a)Sensing performance of FLICE-110% liquid-free ion-conductive elastomers in dry environments. (b) FLICE-110% Liquid-free ion-conductive elastomer encapsulated sensing signal.


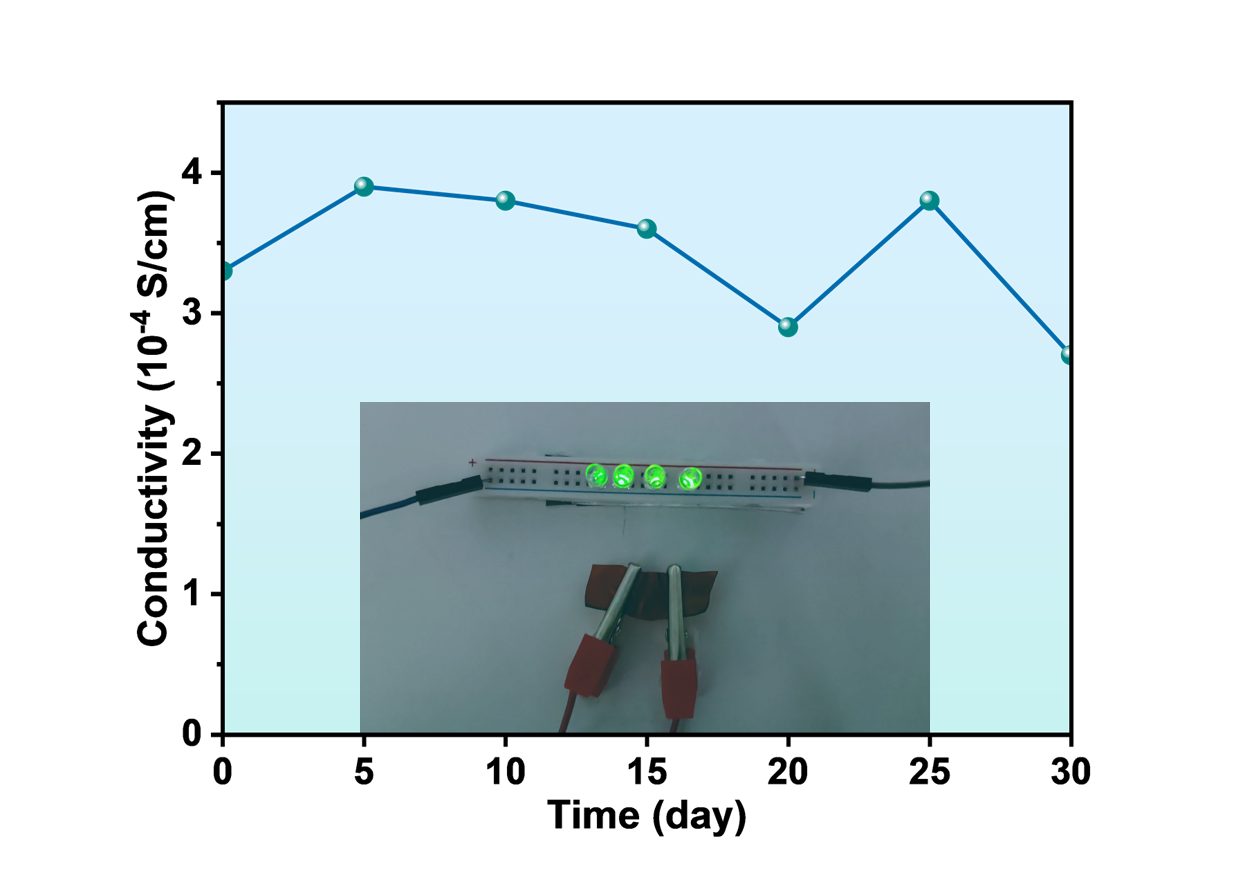


Figure S27. The conductivity of FLICE-110% liquid-free ion-conductive elastomer as a function of time at room temperature. Insert: FLICE -110% liquid-free ion-conductive elastomer can light up four small bulbs.


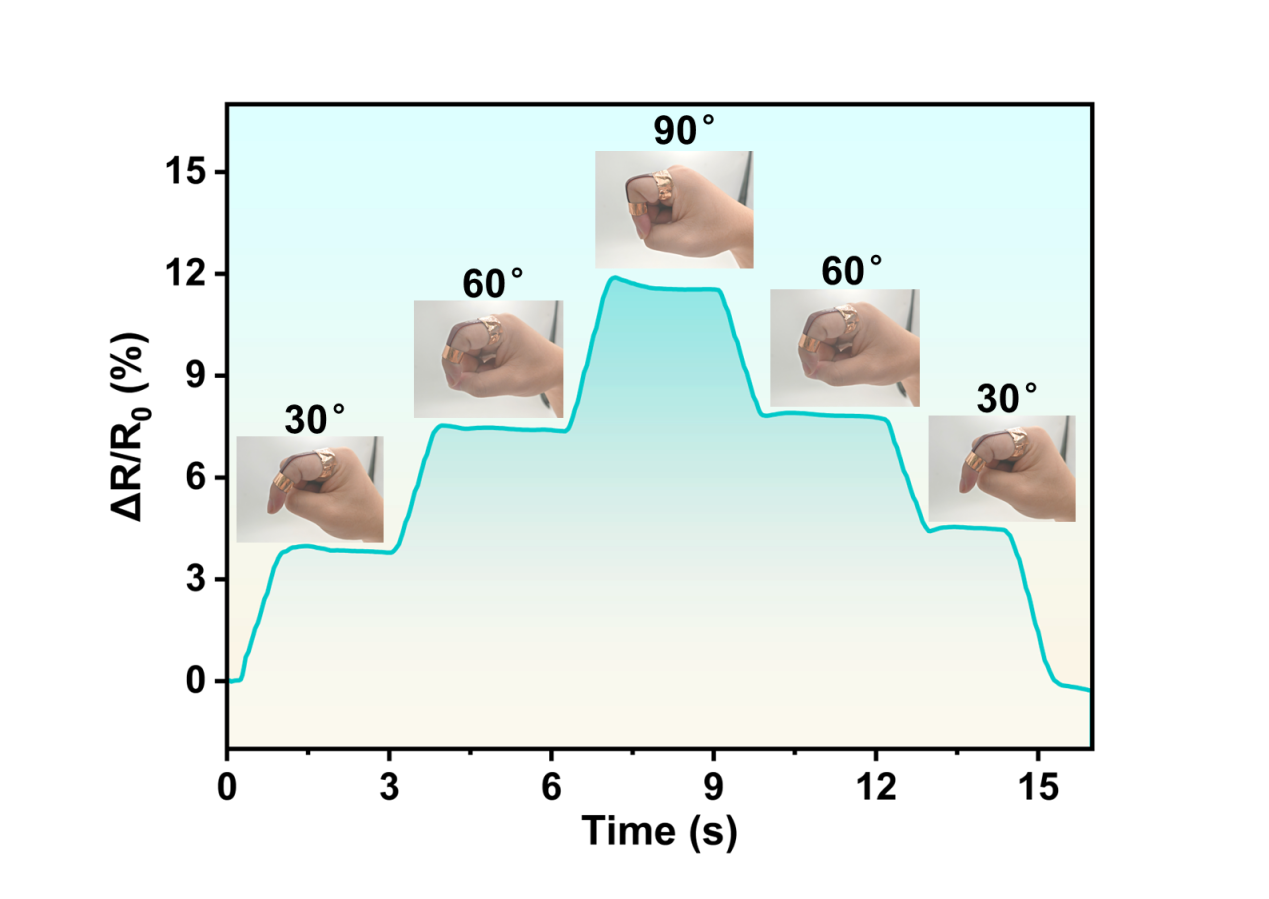


Figure S28. Response of FLICE-110% liquid-free ion-conductive elastomer-based sensors to different bending angles of the finger.


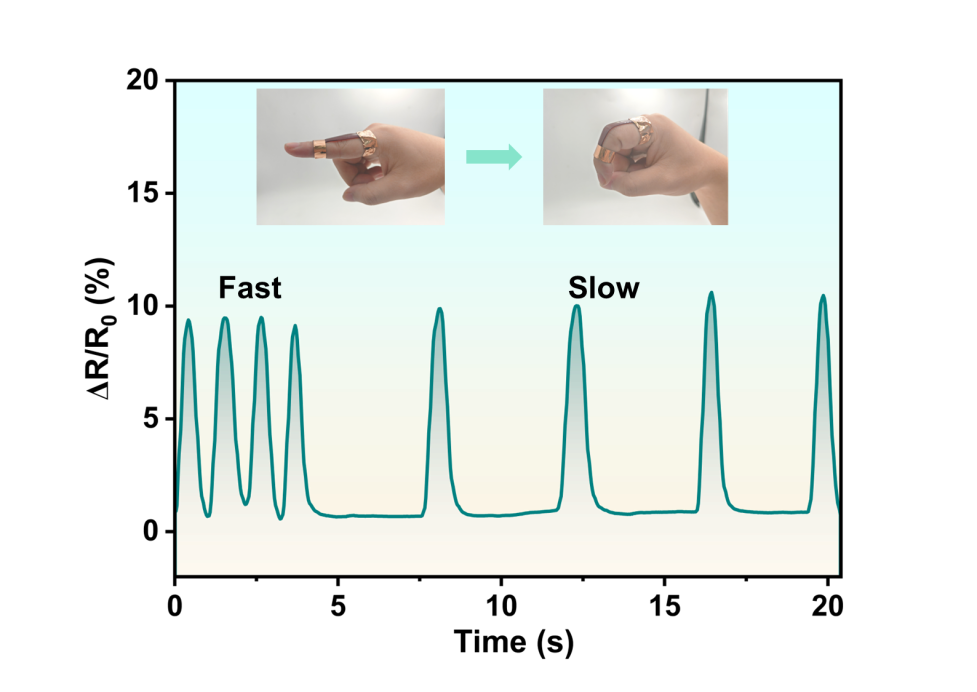


Figure S29. Response of FLICE-110% liquid-free ion-conductive elastomer-based sensors to different bending speeds of the finger.


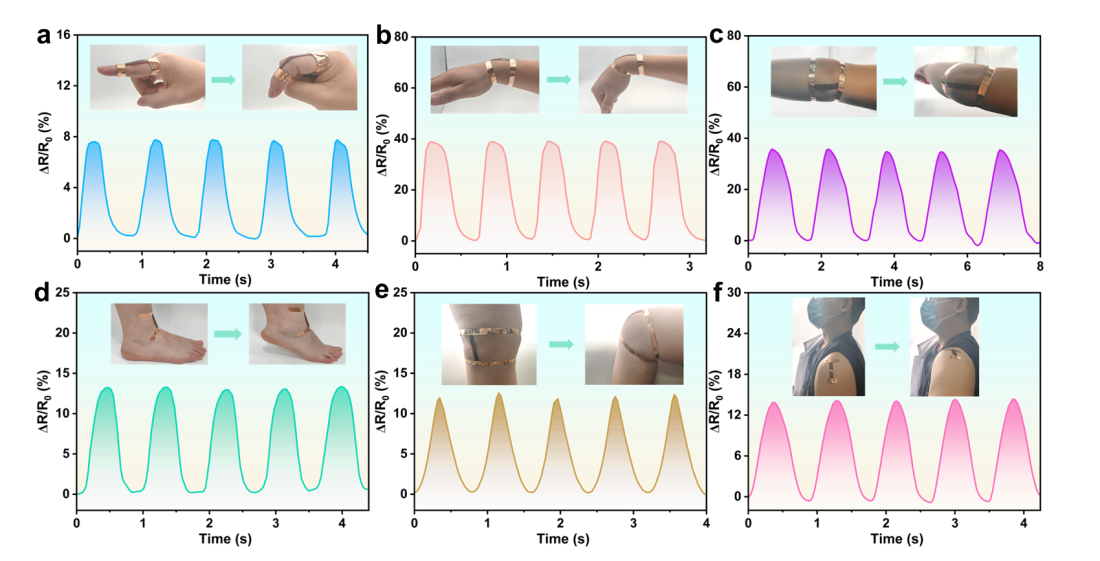


Figure S30. Motion monitoring of different joint parts of the human body such as fingers (a), wrists (b), elbows (c), ankles (d), knees (e) and shoulders (f) by sensors based on FLICE-110% liquid-free ion-conductive elastomer.


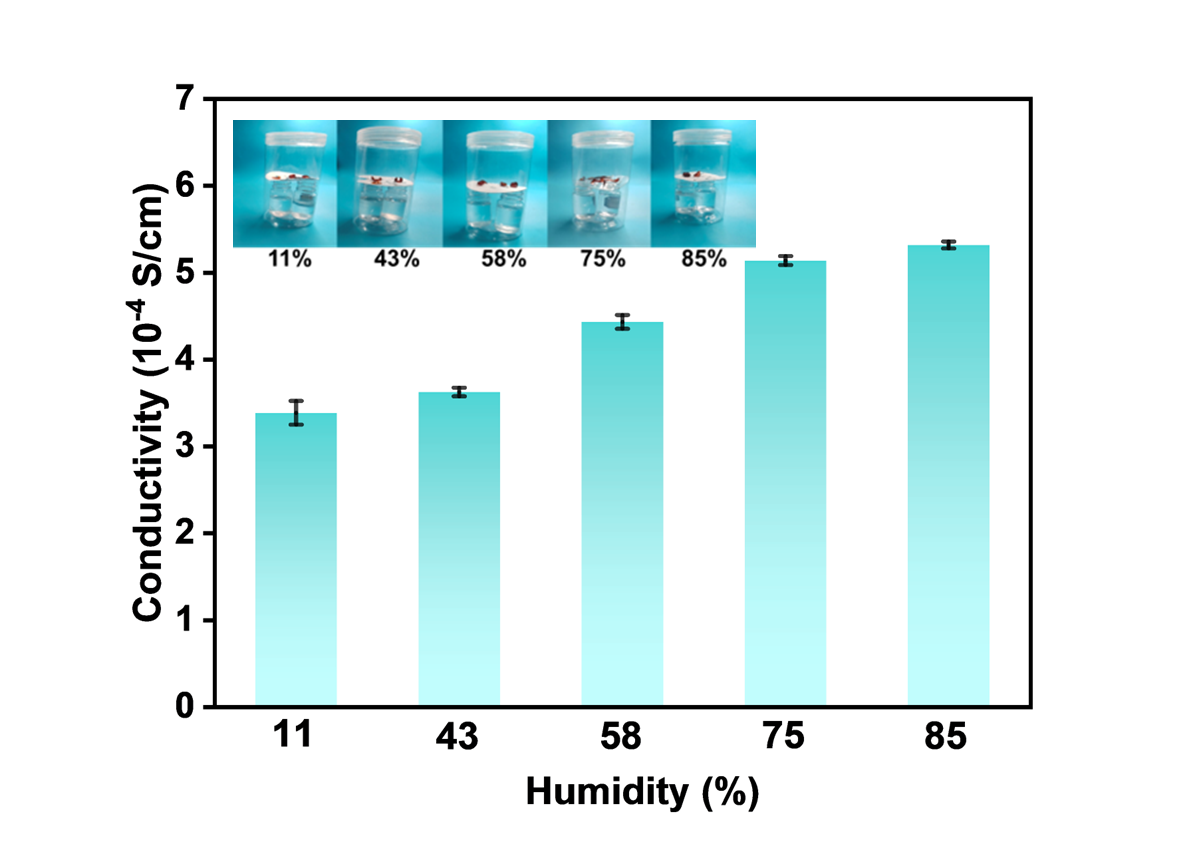


Figure S31. The conductivity of FLICE-110% liquid-free ion-conductive elastomers after 24 hours in different humidity environments. Insert: The inset is a demonstration of environments simulating FLICE-110% liquid-free ion-conductive elastomers at different humidity levels.


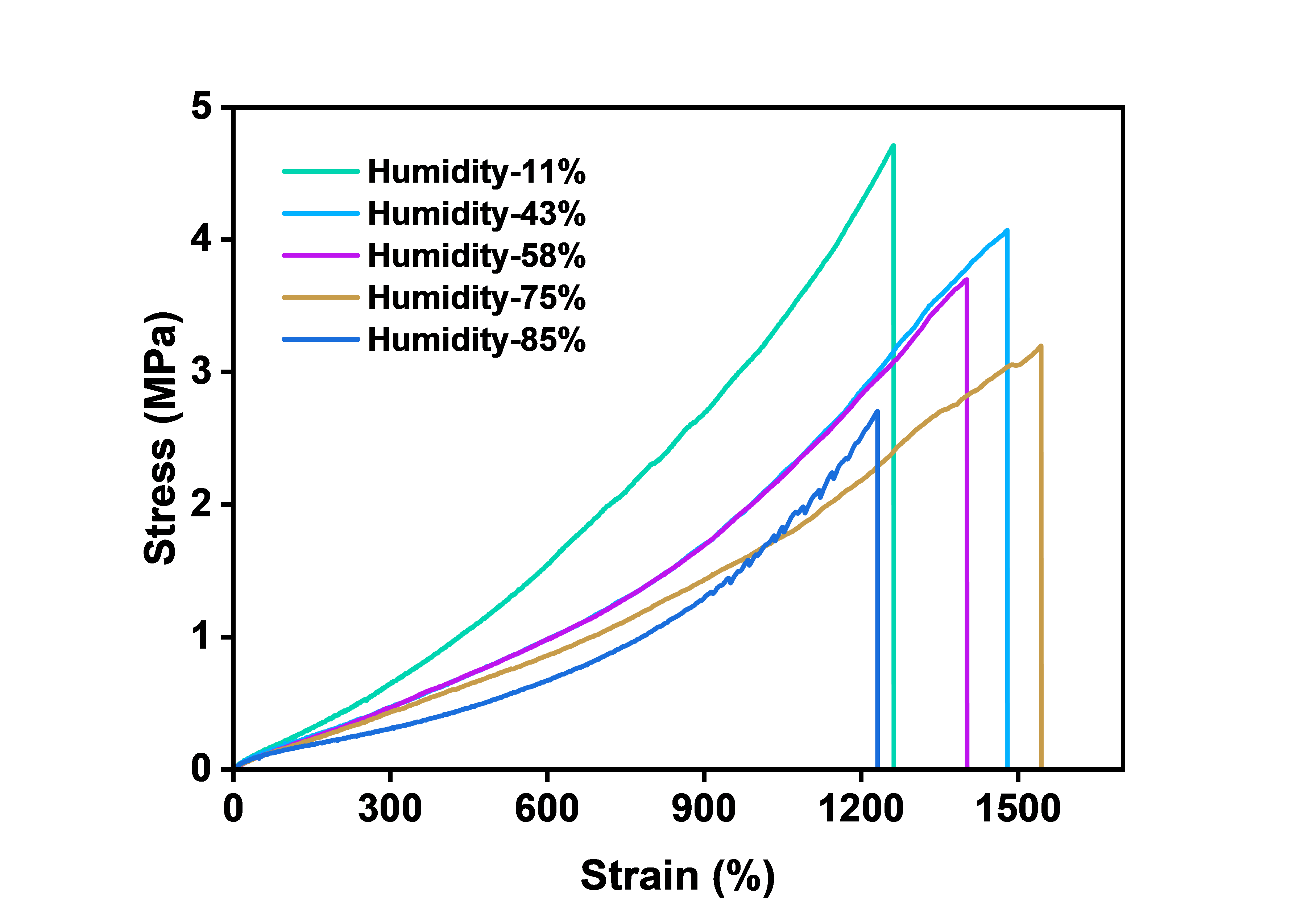


Figure S32. Mechanical properties of FLICE-110% liquid-free ion-conductive elastomer after 24 hours of exposure to different humidity environment.


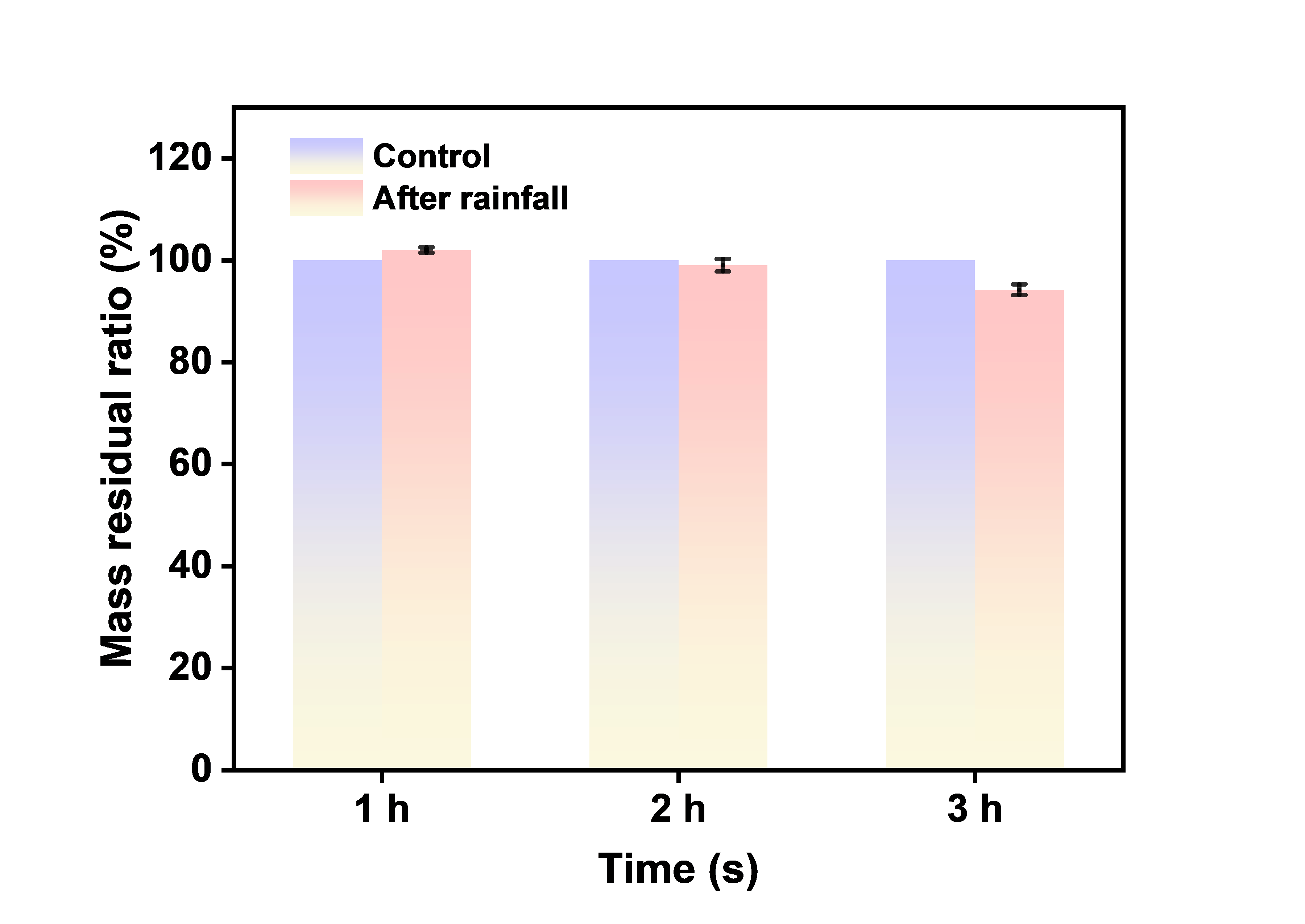


Figure S33. Changes in the mass of FLICE-110% liquid-free ionic conductive elastomer after spraying rainwater for different durations.


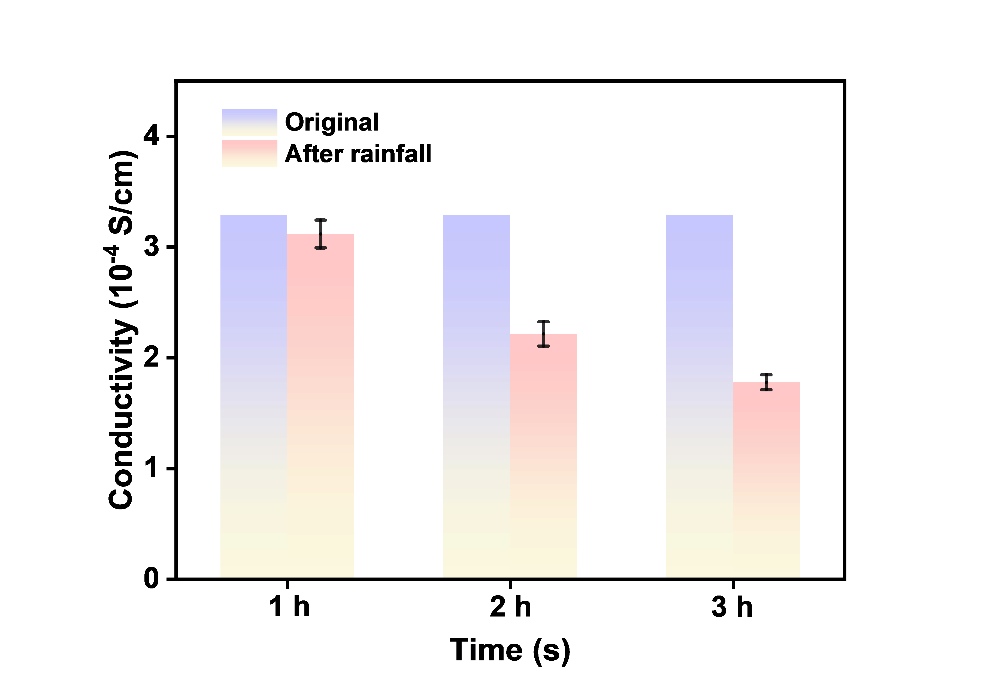


Figure S34. Changes in conductivity of FLICE-110% liquid-free ionic conductive elastomer after spraying rainwater for different durations.


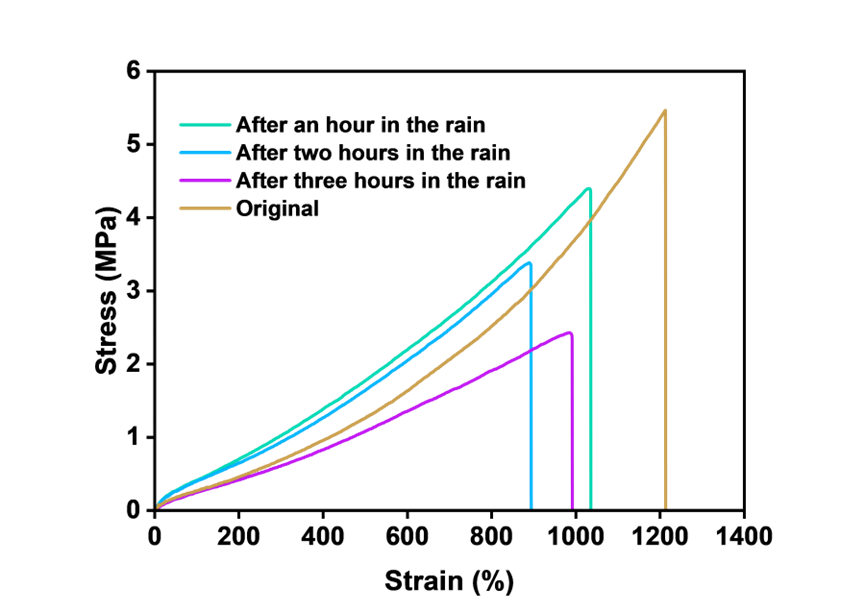


Figure S35. Changes in mechanical properties of FLICE-110% liquid-free ionic conductive elastomer after spraying rainwater for different durations.


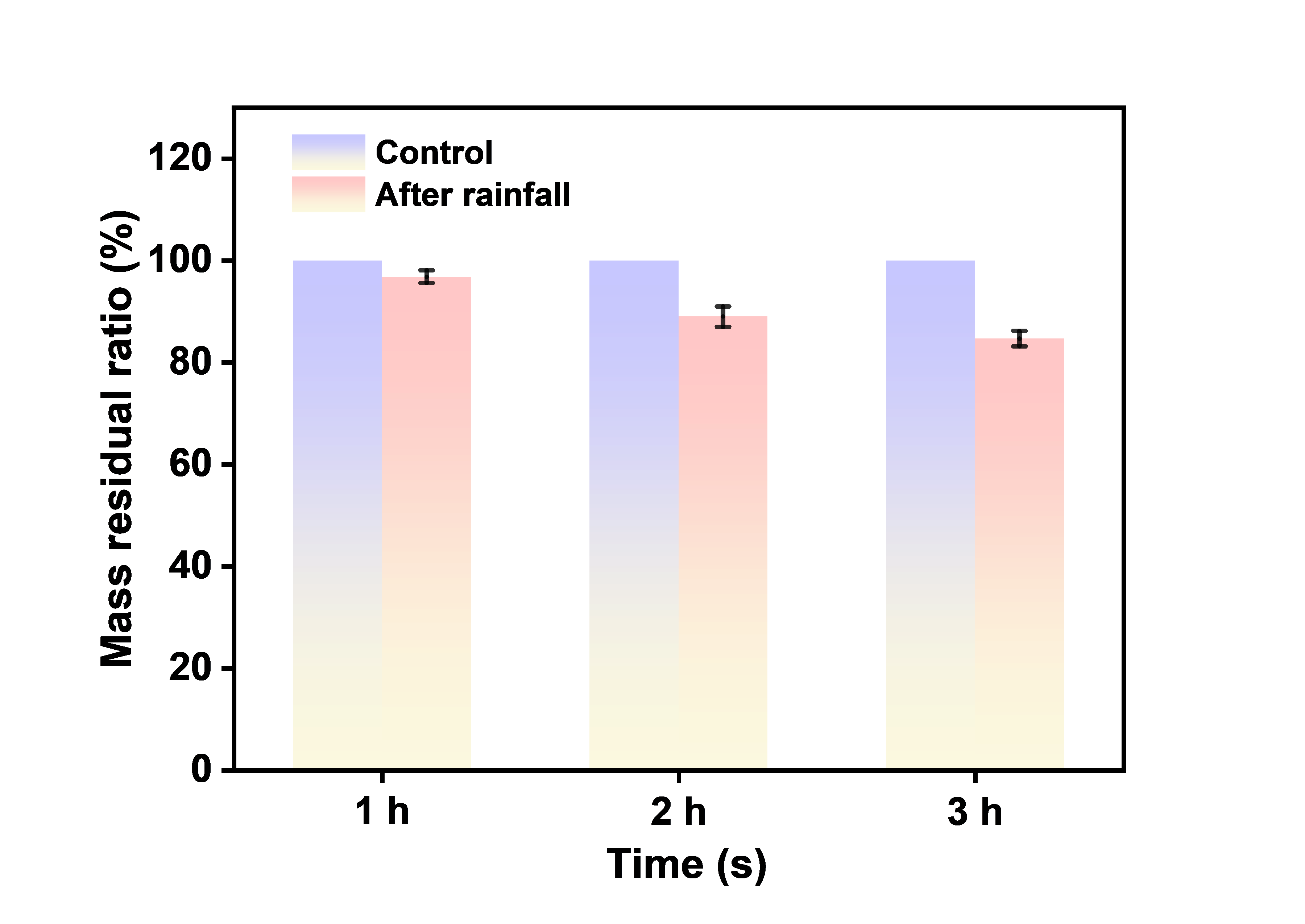


Figure S36. FLICE-110% Liquid-free ionic conductive elastomer mass change after rain spraying and drying.


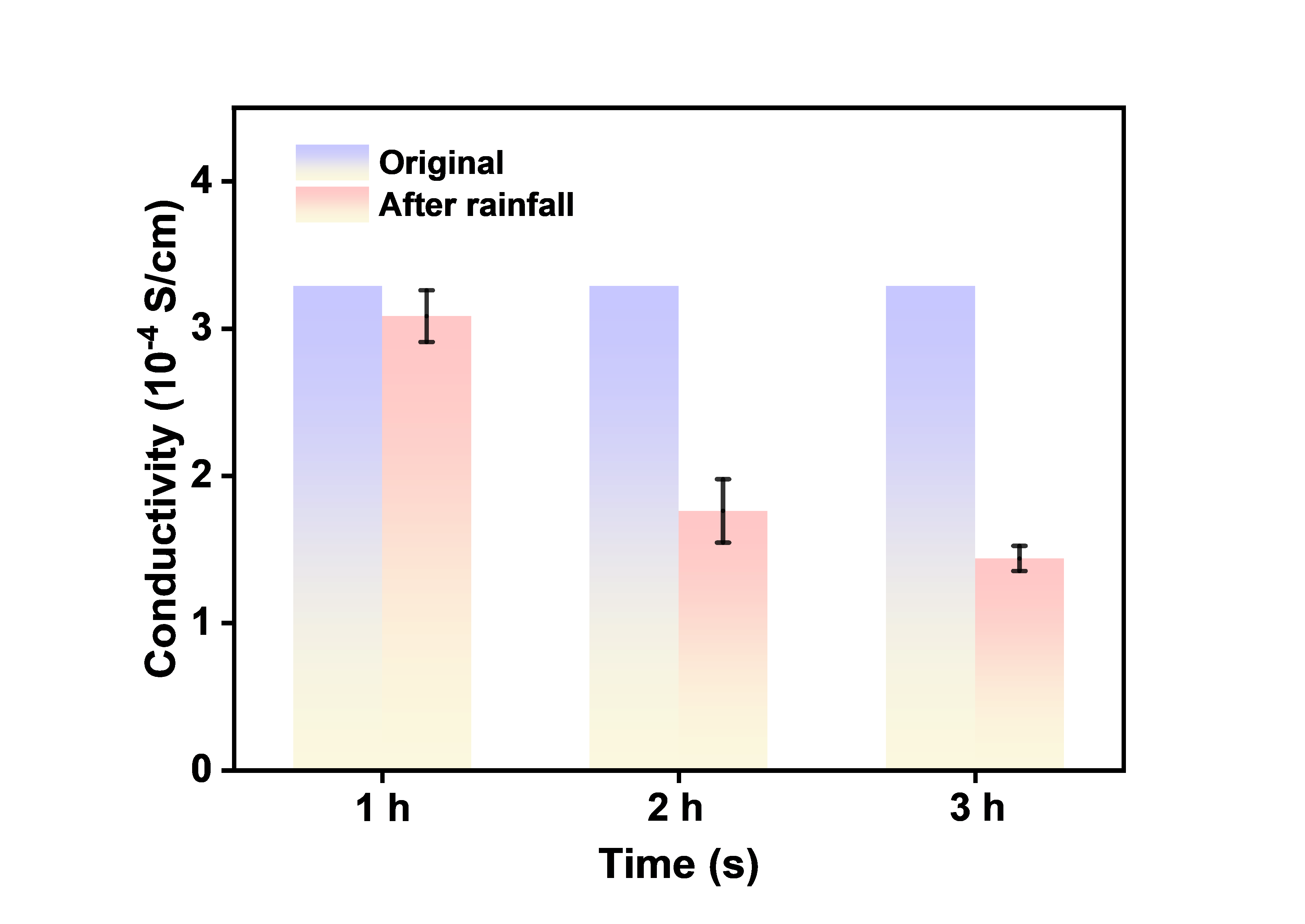


Figure S37. FLICE-110% conductivity change of liquid-free ionic conductive elastomer after rain spraying and drying.


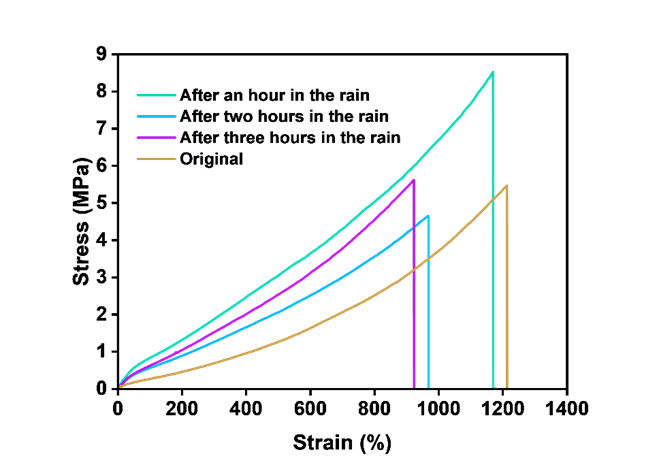


Figure S38. Mechanical changes of FLICE-110% liquid-free ionic conductive elastomer drying after rain spraying.


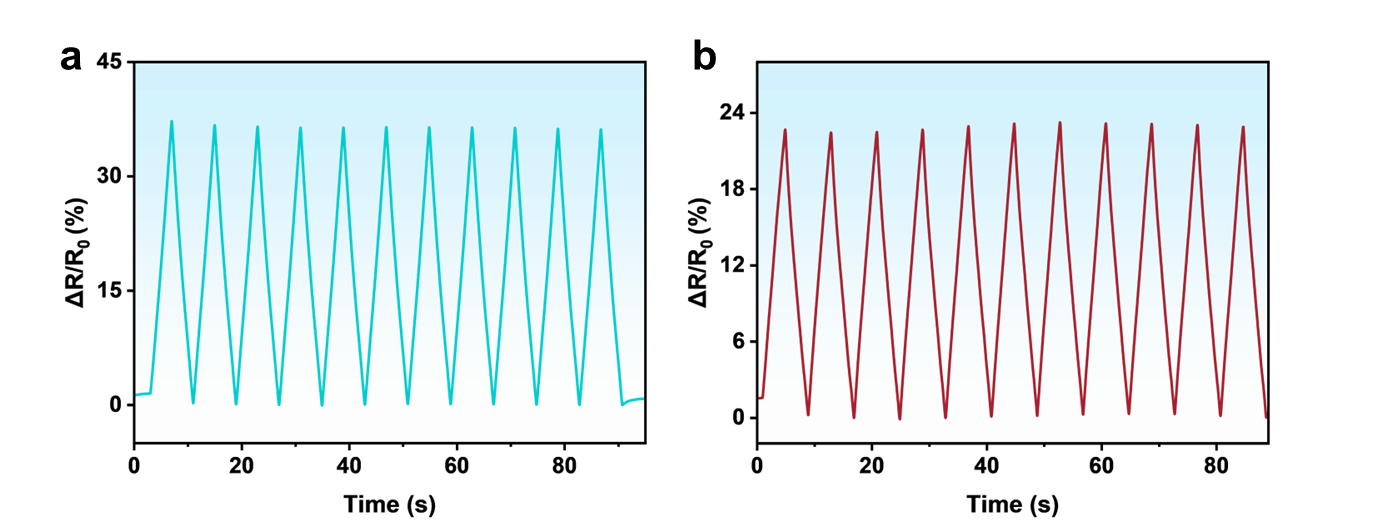


Figure S39. (a) Sensing performance of FLICE-110% liquid-free ion-conductive elastomer in wet weather. (b) Sensing signal of FLICE-110% liquid-free ion-conductive elastomer after rainy weather.


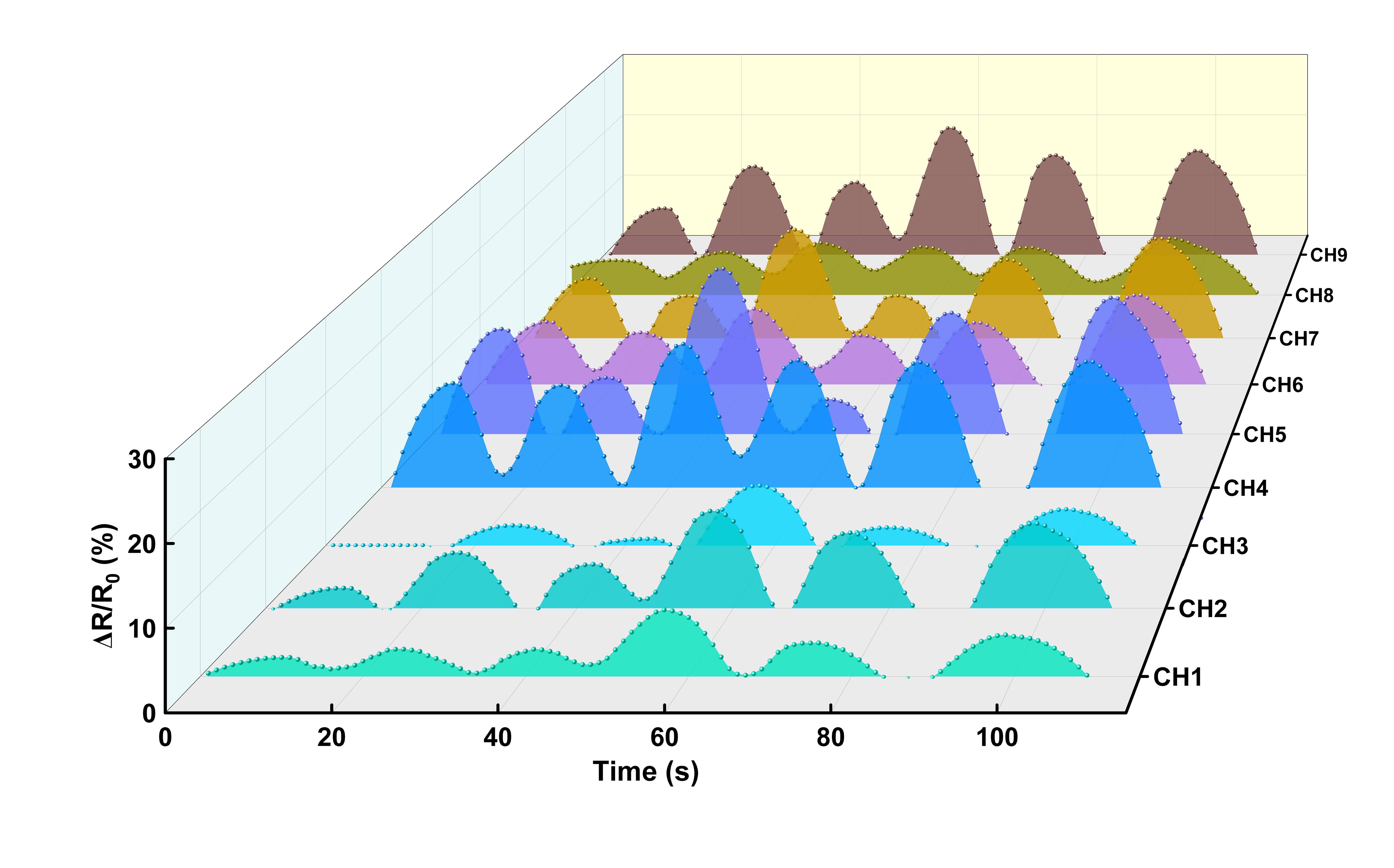


Figure S40. Relative resistance changes were monitored by nine encapsulated strain sensors during the bending of the wing at different positions.


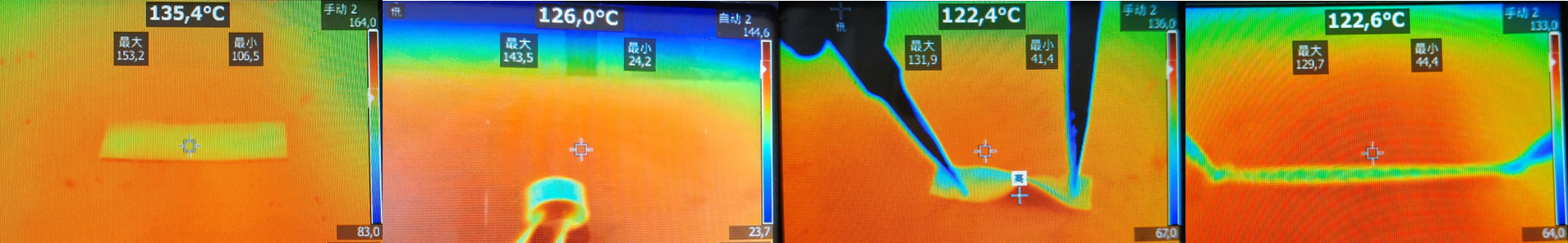


Figure S41. The good bending, twisting, and tensile states of FLICE-110% liquid-free ion-conductive elastomers were demonstrated after being stored at high temperatures for 24 h.


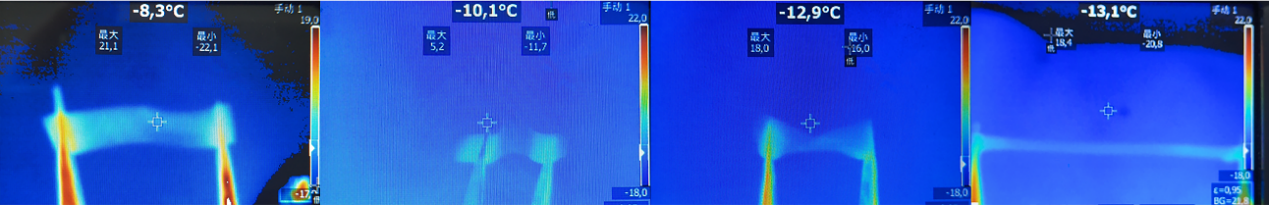


Figure S42. The good bending, twisting, and tensile states of FLICE-110% liquid-free ion-conductive elastomers were demonstrated after being stored at low-temperature environments for 24 h.


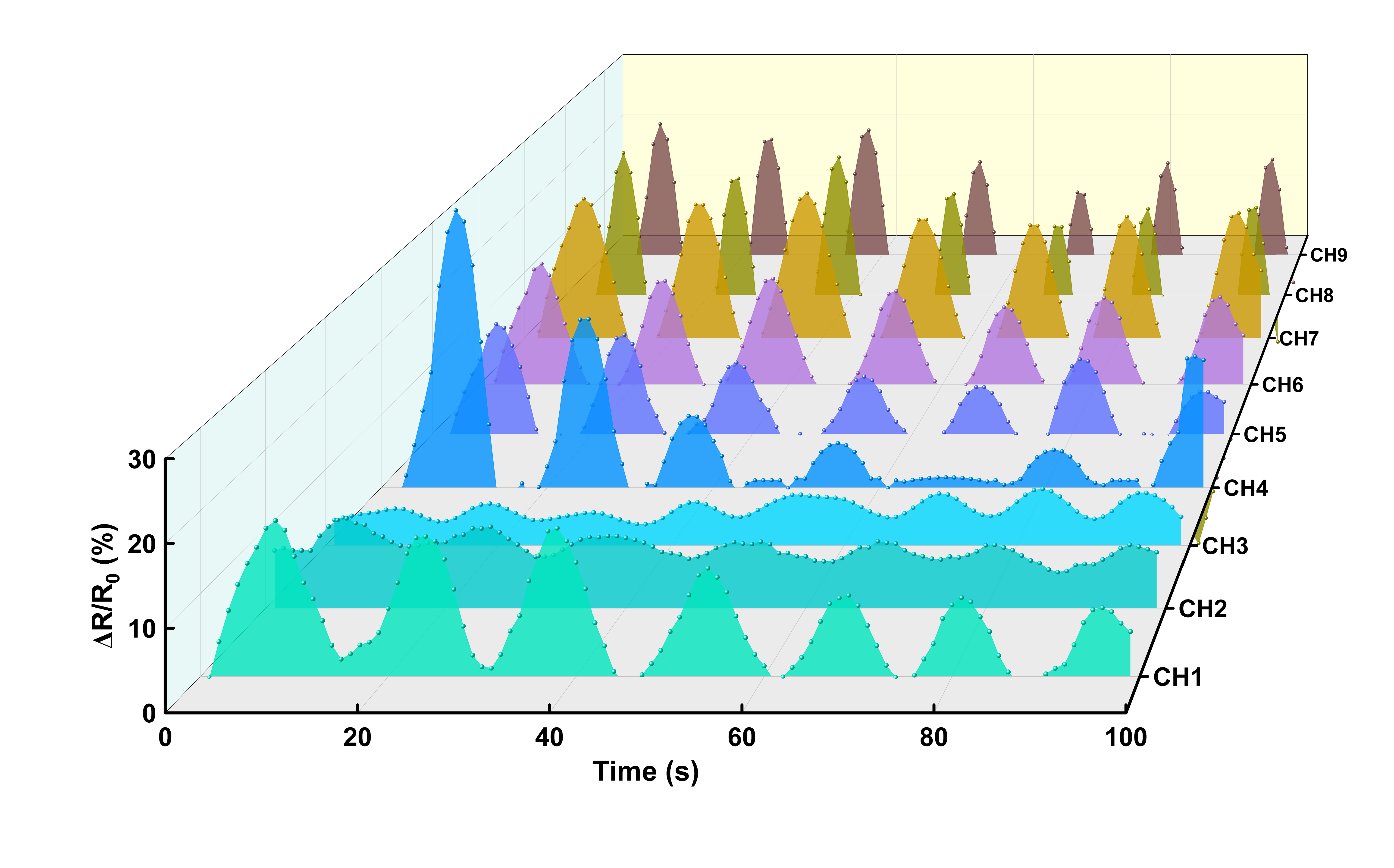


Figure S43. The sensing stability of FLICE-110% liquid-free ion-conductive elastomers was tested by placing them on a high-temperature hot bench for 24 h.


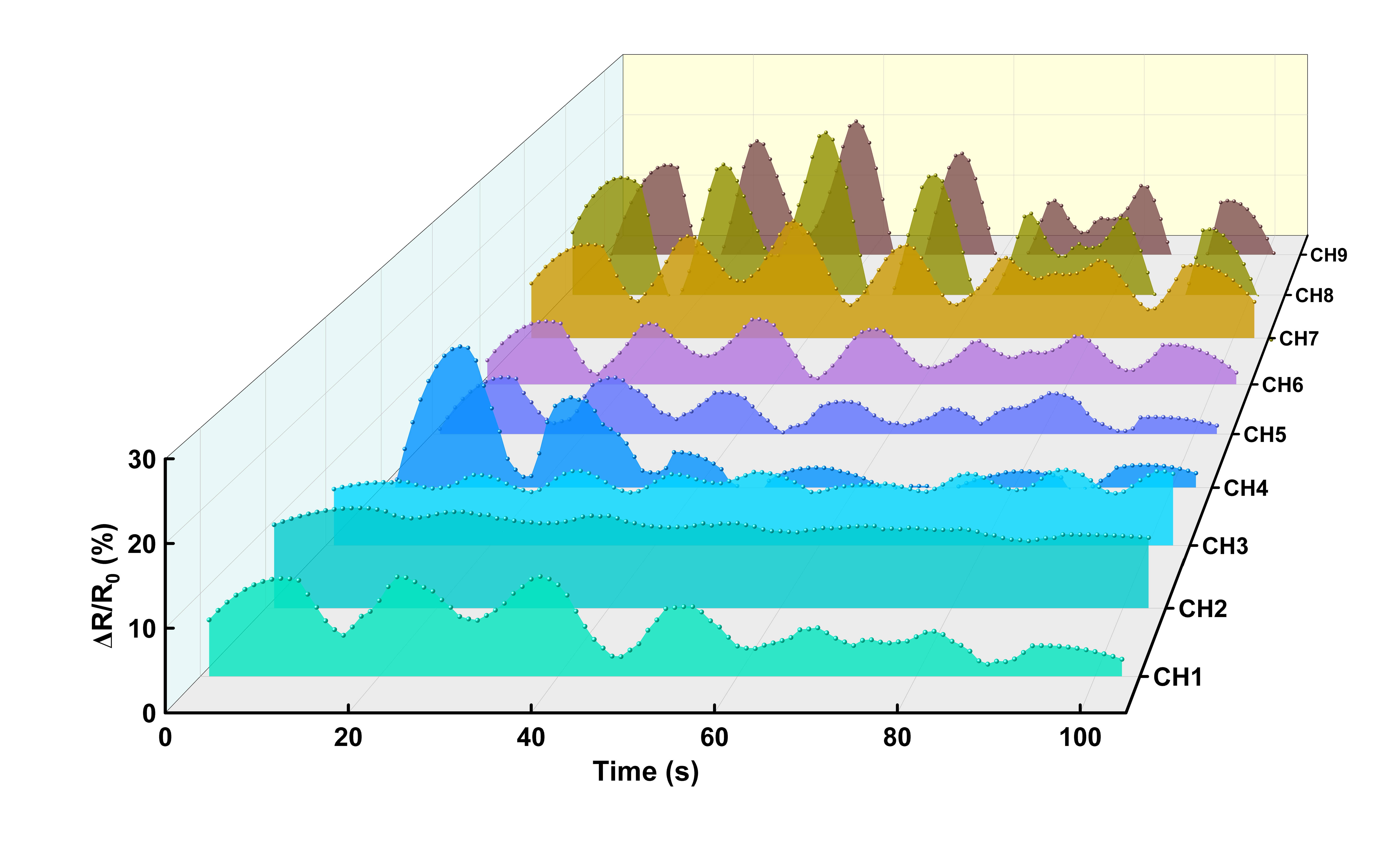


Figure S44. The sensing stability test results of FLICE-110% liquid-free ion-conductive elastomers after being stored in a low-temperature environment for 24 h.


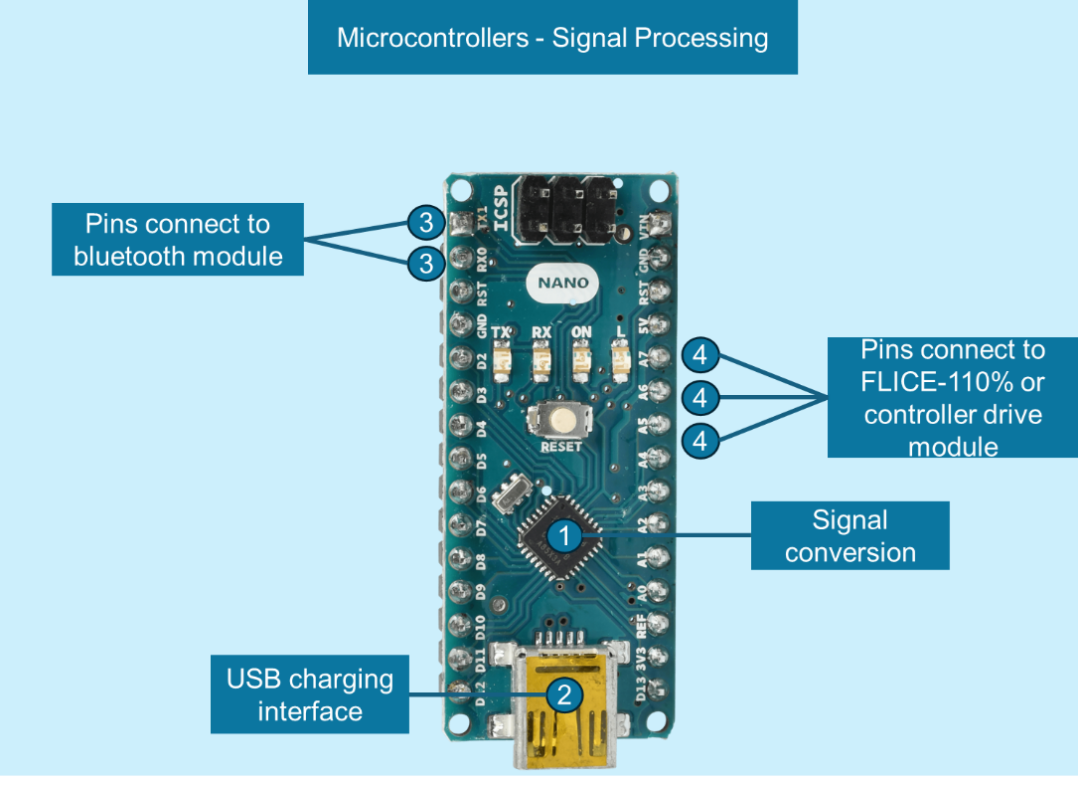


Figure S45. Signal processing and transmission module used in the process of sensor-driven manipulators based on FLICE-110% liquid-free ion-conductive elastomer.


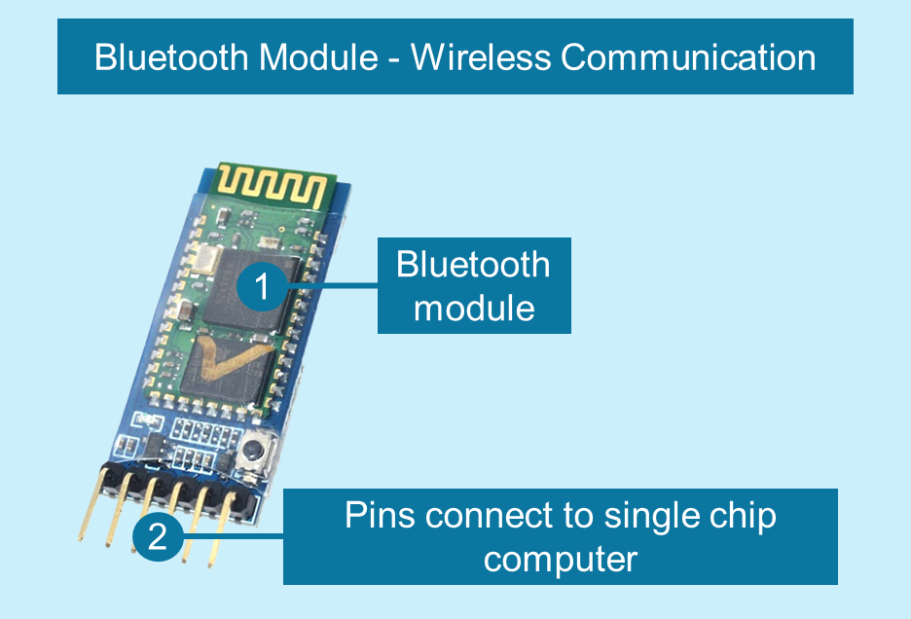


Figure S46. Bluetooth module used in the process of sensor-driven manipulators based on FLICE-110% liquid-free ion-conductive elastomer.


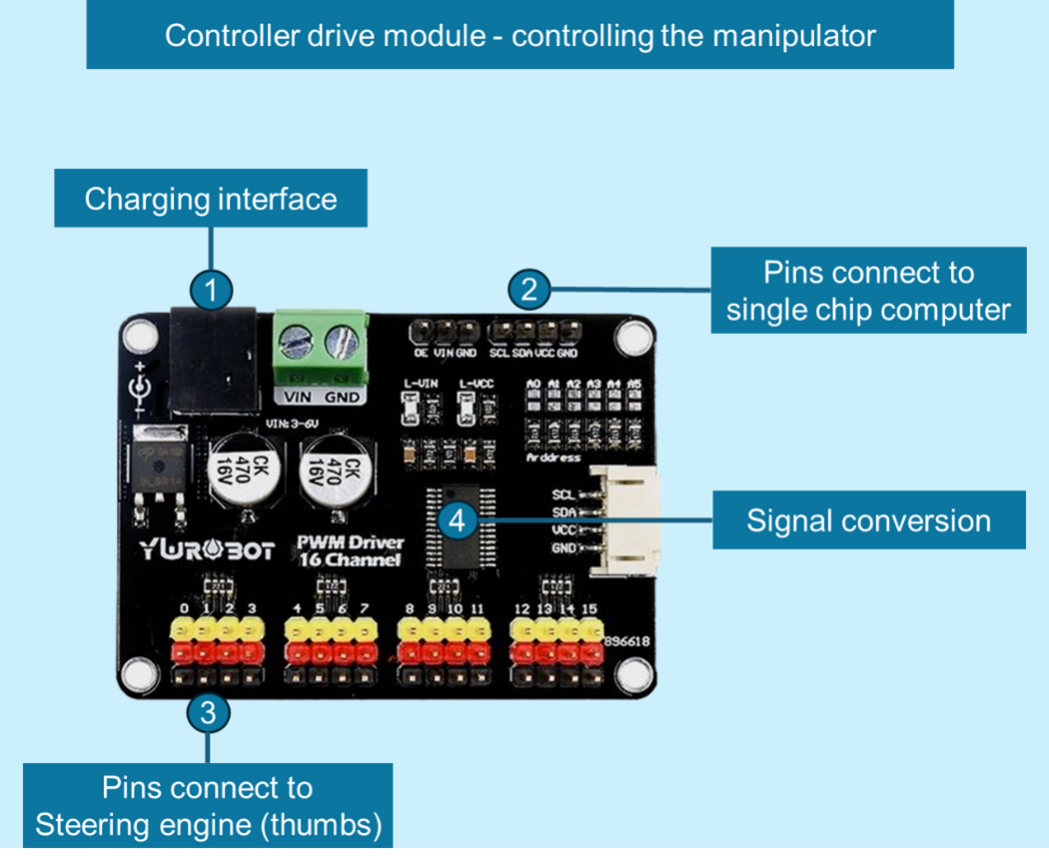


Figure S47. Signal processing and transmission module in the robot.


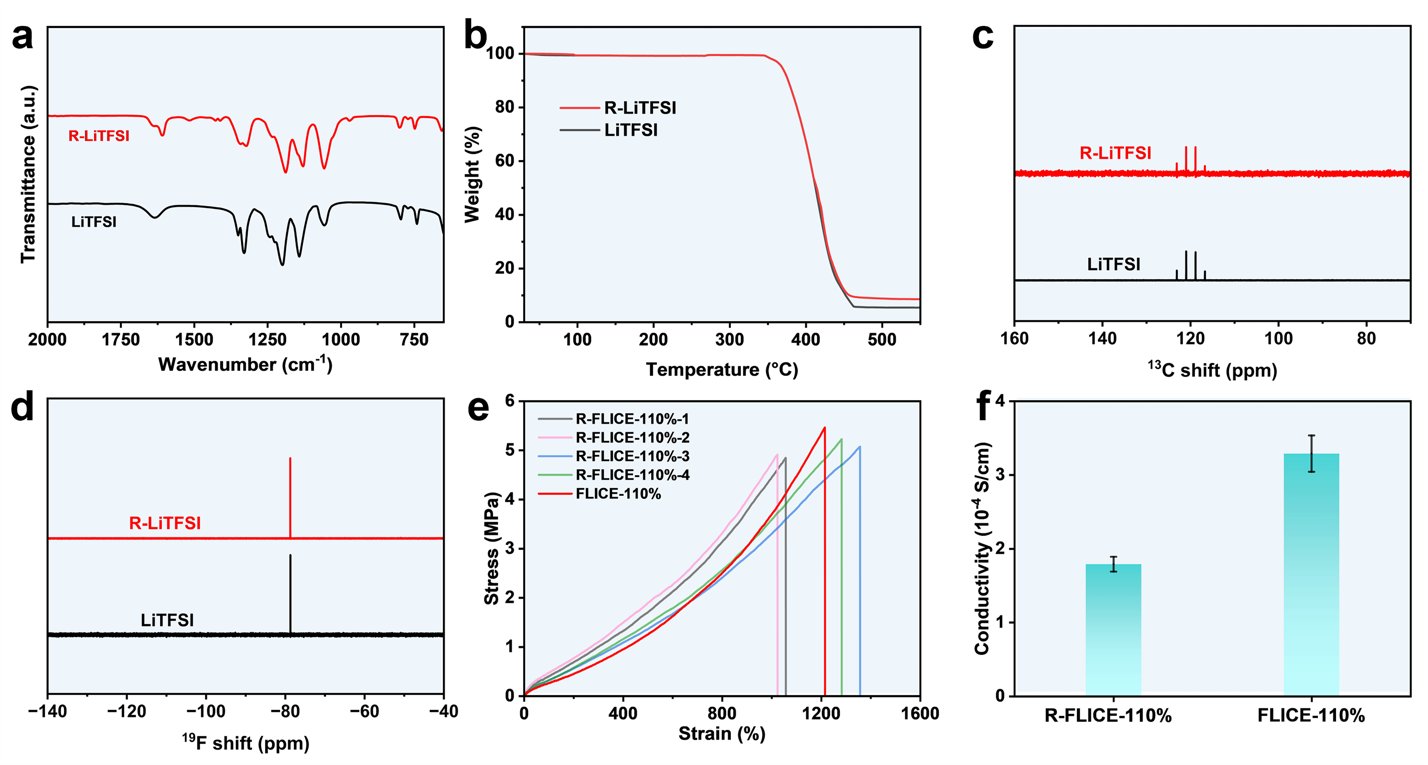


Figure S48. Characterizations of LiTFSI recovered from FLICE-110% liquid-free ion-conductive elastomer. a) FIRT plot of LiTFSI before and after recovery. b) TGA plot of LiTFSI before and after recycling. c) ^13^C NMR plot of LiTFSI before and after recovery. d) ^19^F NMR plot of LiTFSI before and after recovery. e) Comparison of mechanical properties of R-FLICE-110% and FLICE-110% liquid-free ion-conductive elastomer. f) Comparison of electrical conductivity of R-FLICE-110% and FLICE-110% liquid-free ion-conductive elastomer.


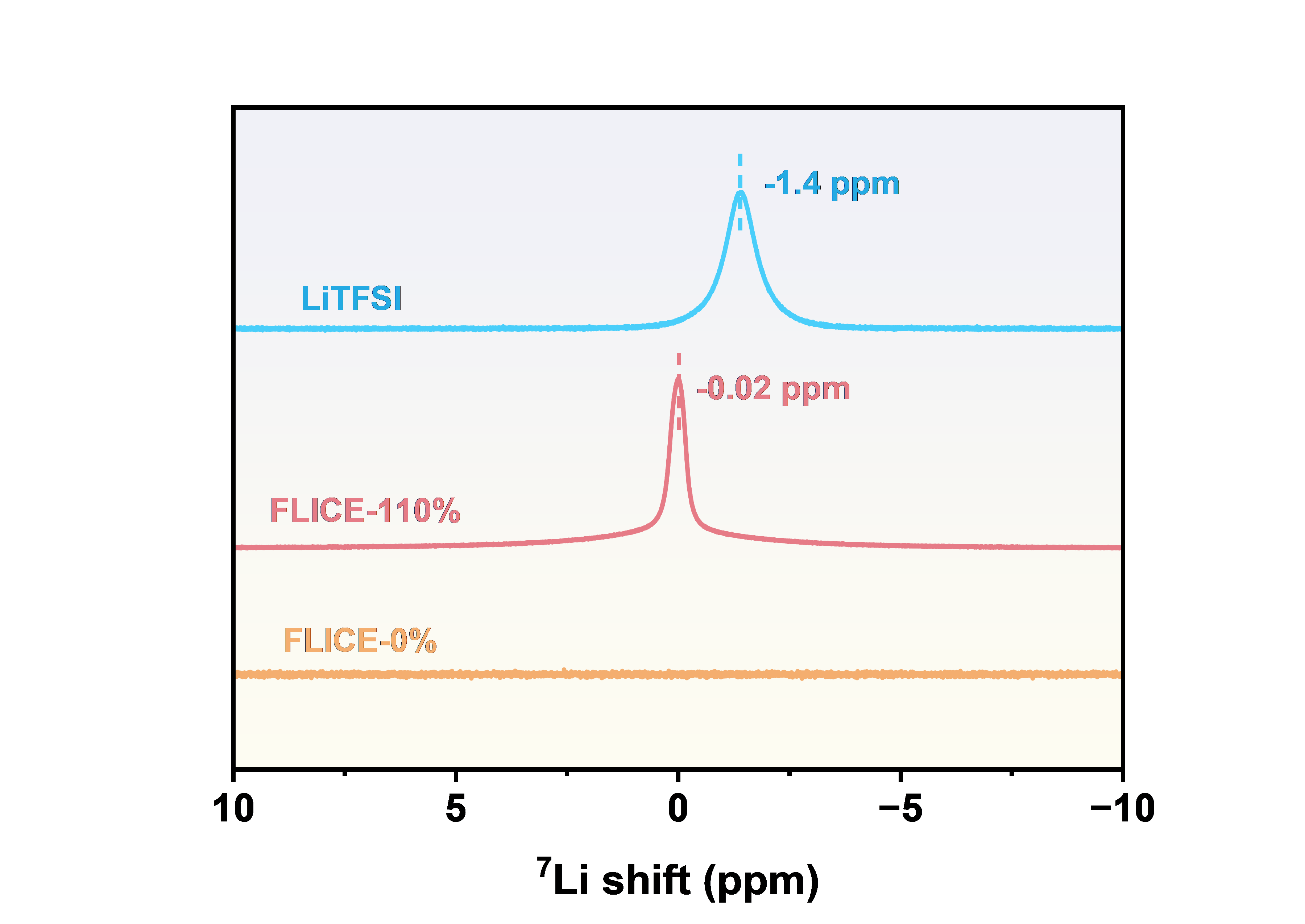


Figure S49． ^7^Li NMR spectra of LiTFSI、FLICE-110% and FLICE-110%.


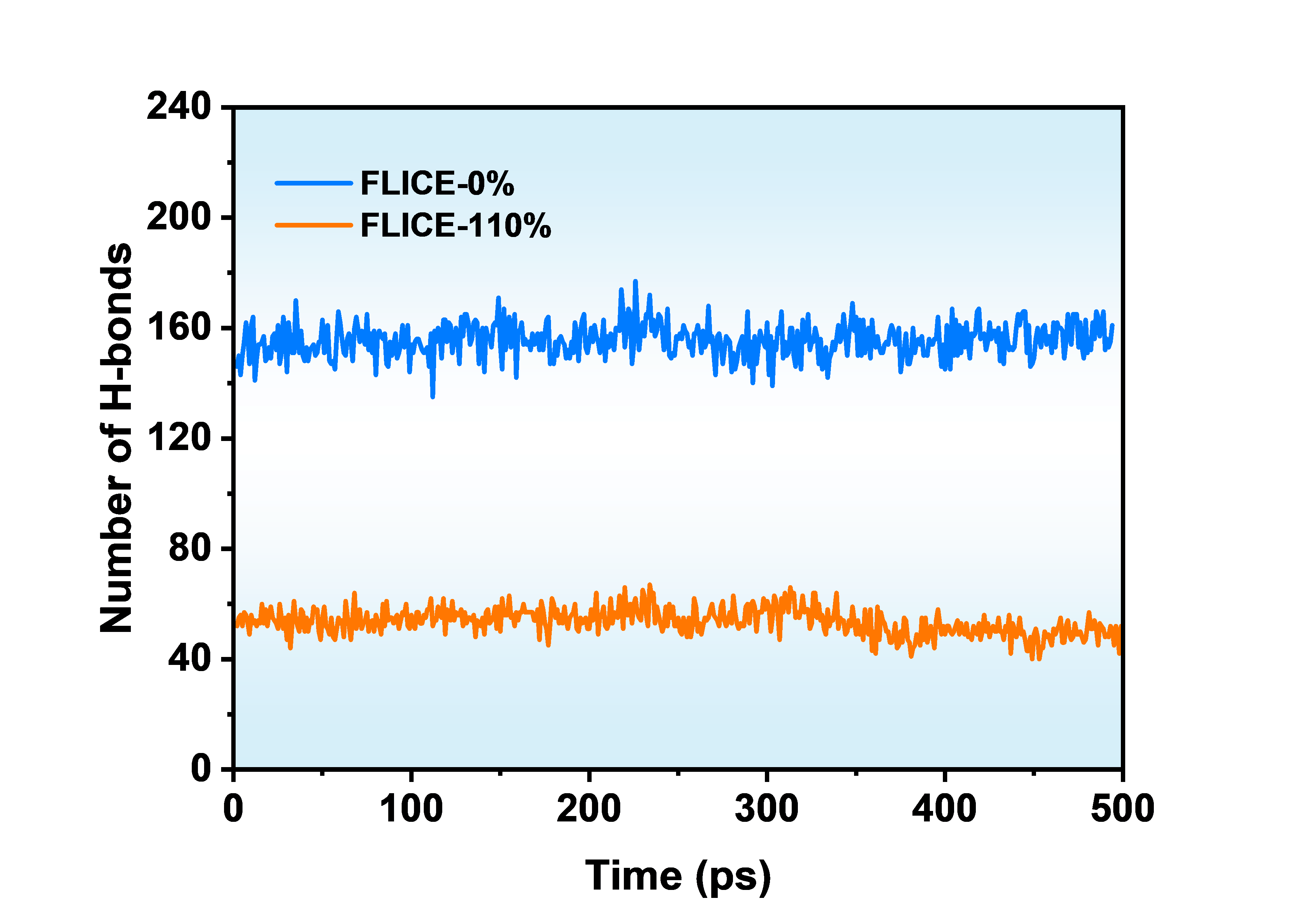


Figure S50． Number of hydrogen bonds in FLICE-0% elastomers and FLICE-110% liquid-free ion-conductive elastomers.


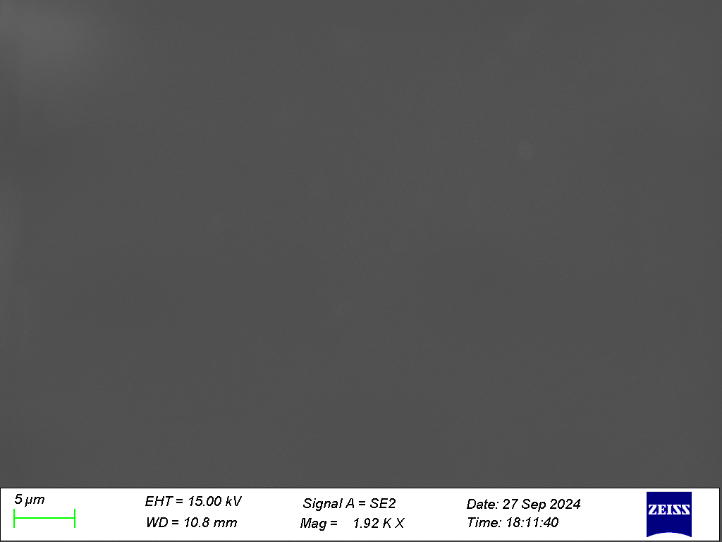


Figure S51. SEM of FLICE-110% liquid-free ion-conductive elastomer.


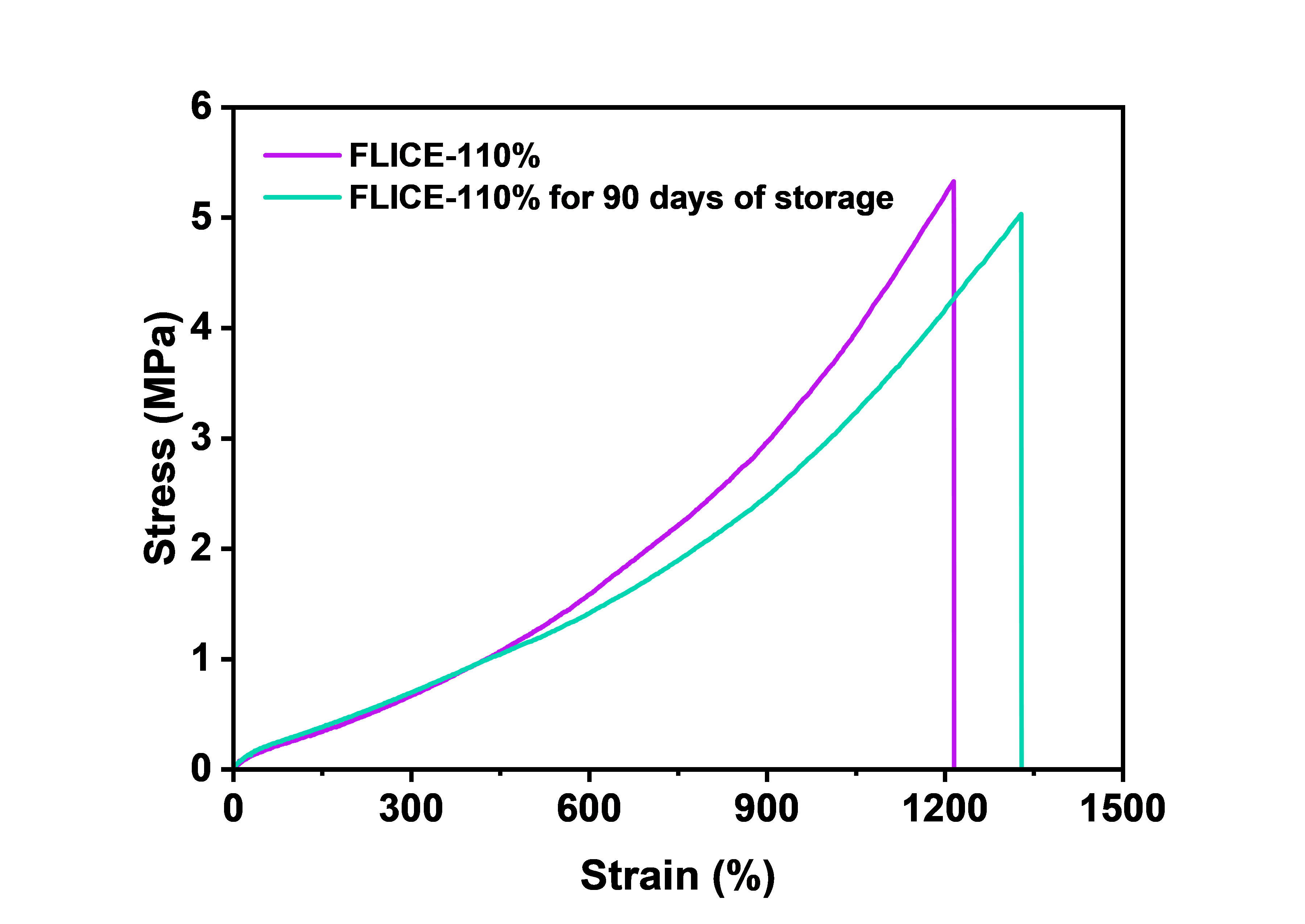


Figure S52. The plot of mechanical properties of FLICE-110% liquid-free ion-conductive elastomer after 90 days of storage compared to initial FLICE-110%.


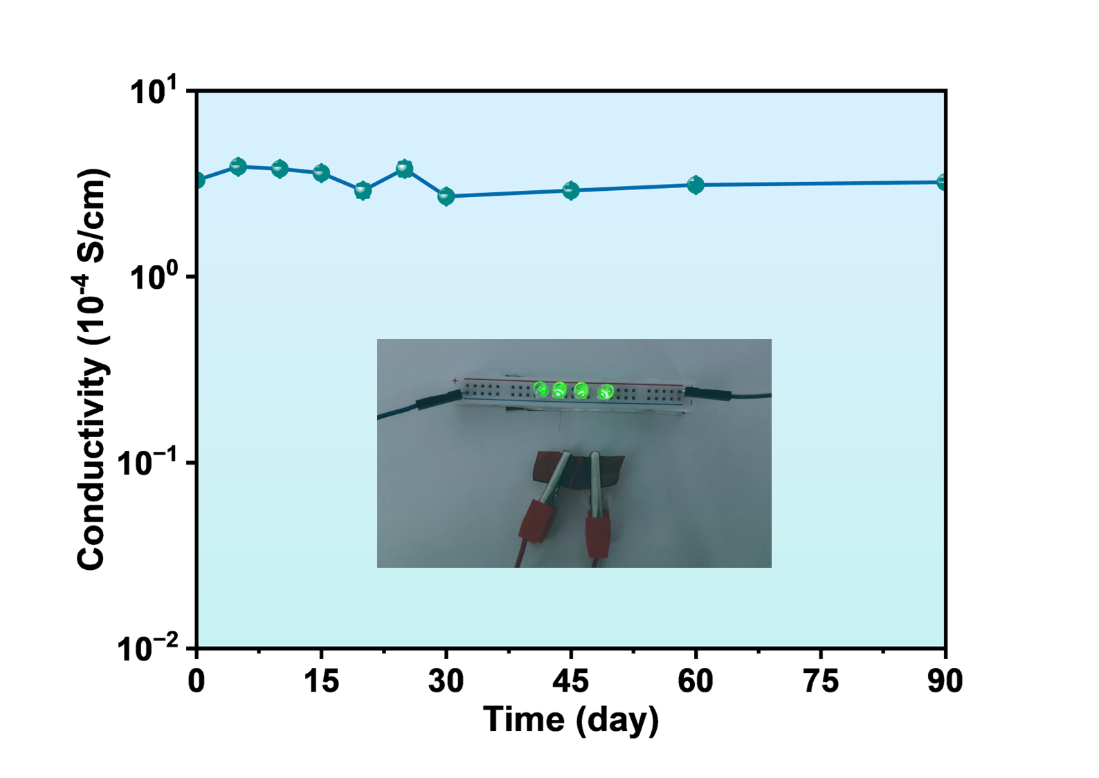


Figure S53. The conductivity of FLICE-110% liquid-free ion-conductive elastomers as a function of time at room temperature.

# Supplementary Movie

**Movie S1.** Movie demonstration of a sensor-driven manipulator based on FLICE-110% liquid-free ion-conductive elastomer.

# 5. **References**

1. C. W. Lu, X. Y. Wang, Q. Q. Jia, S. J. Xu, C. P. Wang, S. Du, J. F. Wang, Q. Yong, F. X. Chu, *Carbohydr. Polym.* **2024**, 324, 121496.
2. Y. Xu, S. Zhou, Z. Wu, X. Yang, N. Li, Z. Qin, T. Jiao, *Chem. Eng. J.* **2023**, 466, 143179.
3. Z. Li, Y. L. Zhu, W. Niu, X. Yang, Z. Jiang, Z. Y. Lu, X. Liu, J. Sun, *Adv. Mater.* **2021**, 33, 2101498.
